# Inducible Forward Programming of Human Pluripotent Stem Cells to Hemato-endothelial Progenitor Cells with Hematopoietic Progenitor Potential

**DOI:** 10.1016/j.stemcr.2020.05.019

**Published:** 2020-07-14

**Authors:** Lucas Lange, Dirk Hoffmann, Adrian Schwarzer, Teng-Cheong Ha, Friederike Philipp, Daniela Lenz, Michael Morgan, Axel Schambach

## Main Text

(Stem Cell Reports *14*, 122–137; January 14, 2020)

The authors wish to apologize for errors in the Supplemental Experimental Procedures. The units of the antibiotic concentrations were accidentally misrepresented as being in ng and g instead of μg. The units have now been corrected as follows:

“Long term iPSC cultivation was performed as ML in CM in the presence of puromycin (0.3 μg mL^−1^) and/or zeocin (0.5 μg mL^−1^) (both InvivoGen, Toulouse, France) to prevent vector-silenced SLGE-iPSC throughout the cultivation.”

“Puromycin (0.3 μg mL^−1^) and zeocin (0.5 μg mL^−1^) (both InvivoGen) were applied 24 h post-transduction.”

These corrections do not influence the validity of the results or conclusions of this paper.

