# Supplementary material for: Inducible Forward Programming of Human Pluripotent Stem Cells to Hemato-endothelial Progenitor Cells with Hematopoietic Progenitor Potential
Source: Stem Cell Reports. 2019 Dec 12;14(1):122–37. doi: 10.1016/j.stemcr.2019.11.005 (PMC6962646; doi:10.1016/j.stemcr.2019.11.005)
Supplement: Document S1. Supplemental Experimental Procedures, Figures S1–S6, and Tables S1 and S2 [file mmc1.pdf]

Stem Cell Reports, Volume *14*

Supplemental Information

Inducible Forward Programming of Human Pluripotent Stem Cells to Hemato-endothelial Progenitor Cells with Hematopoietic Progenitor Potential

Lucas Lange, Dirk Hoffmann, Adrian Schwarzer, Teng-Cheong Ha, Friederike Philipp, Daniela Lenz, Michael Morgan, and Axel Schambach

Inventory of Supplemental Information

- Figure S1 Characterization of the Dox-inducible vector-system and SLGE-iPSC lines. Related to Figure 1
- Figure S2 Kinetic analysis of immunophenotype during Phase I of hemato-endothelial forward programming. Related to Figure 2
- Figure S3 Vascular endothelial potential and reproducibility of the forward programming protocol. Related to Figure 3
- Figure S4 Characterization of the HPC potential and function. Related to Figure 5
- Figure S5 Sorting strategy and gating scheme for the RNA-sequencing approach. Related to Figure 6
- Figure S6 RNA-sequencing analysis of SLGE-iPSC derived HEP/HPC and cord blood HSC. Related to Figure 6
- Supplemental experimental procedures
    - Table S1: Flow Cytometry Antibody
    - Table S2: Primer/Oligonucleotide
- Supplemental References
- Table S3: Gene Sets for Gene Set Enrichment Analysis (separate file)

Figure S1 Characterization of the Dox-inducible vector-system and SLGE-iPSC lines. Related to Figure 1

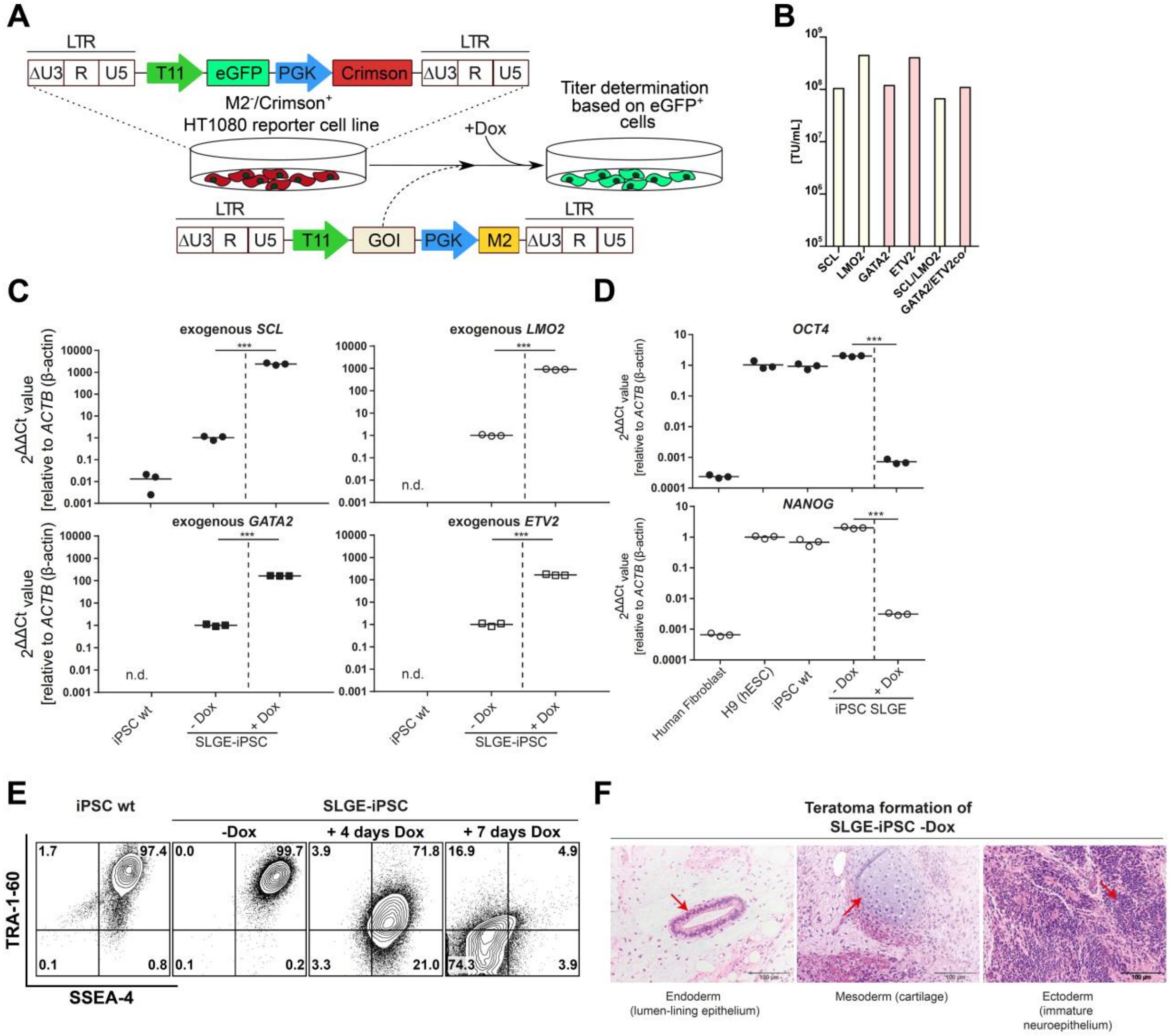

Figure S1 Characterization of the Dox-inducible vector-system and SLGE-iPSC lines. Related to Figure 1. (A) HT1080 reporter cell line for titration of all-in-one Dox-inducible vectors. Reporter contains an eGFP cassette under the control of a Dox-inducible T11 promoter and detects the biological activity of the M2 transactivator of the all-in-one vectors upon successful transduction and transgene expression. **(B)** Titer (Transducing units (TU) per mL) of all-in-one TF expressing vectors. **(C)** Inducibility of ectopic SLGE expression upon Dox stimulation, determined by quantitative RT-PCR. Cells were stimulated for 7 days with Dox (+Dox). Expression was normalized to –Dox control (basal expression), n.d. = not detected; samples were measured in triplicates. P-values were calculated using one-way ANOVA with Bonferroni's Multiple Comparison Test. **(D)** Comparison of endogenous expression level of human pluripotency genes *OCT4* and *NANOG* (determined by quantitative RT PCR) between wild type iPSC (wt) and SLGE-iPSC (-/+ Dox). Human *ACTB* (β-Actin) was used as housekeeping gene, and expression levels were normalized to H9 hESC as a pluripotency control in triplicate samples (mean of replicates). P-values were calculated using one-way ANOVA with Tukey's multiple comparisons test. **(E)** Expression of pluripotency surface markers TRA-1-60 and SSEA-4 on H2E6C wild type iPSC and H2E6C SLGE iPSC (-/+ Dox) in iPSC-maintenance culture (gated on unstained control). **(F)** Representative H&E staining of teratoma showing cells derived from all three germ-layers after injection of SLGE iPSC (Dox) into immunocompromised NSGS mice (scale bar 100 µm).

Figure S2 Kinetic analysis of immunophenotype during Phase I of hemato-endothelial forward programming. Related to Figure 2

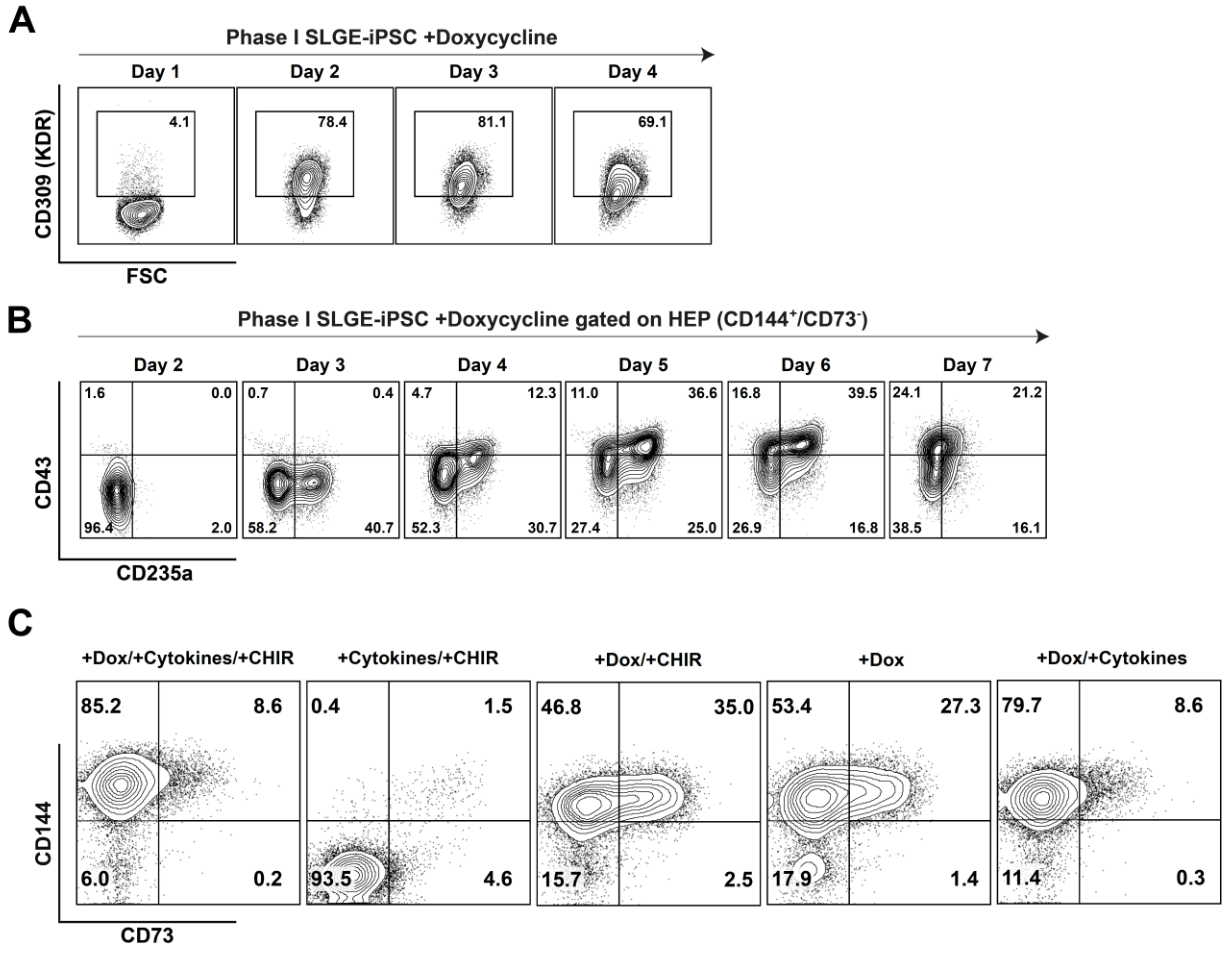

Figure S2 Kinetic analysis of immunophenotype during Phase I of hemato-endothelial forward programming. Related to Figure 2 (A) Kinetic FCM analysis of the mesodermal priming based on the expression of the vascular endothelial growth factor receptor 2 (KDR) during the first four days of Phase I. **(B)** Kinetic analysis of the immunophenotype of CD43/CD235a expression during the seven days of Phase I in $CD144^{+}/CD73^{-}$ SLGE-HEP (+Dox). **(C)** Influence of SLGE induction (+Dox), Cytokines and small molecule (CHIR) on the hemato-endothelial specification (Phase I day 7, $CD144^{+}/CD73^{-}$). Cytokines (SCF, FGF2, TPO, VEGF and IL-3) and/or small molecule (CHIR) were removed during Phase I to evaluate the effects of the components on hemato-endothelial specification. Gates were set based upon FMO controls.

Figure S3 Vascular endothelial potential and reproducibility of the forward programming protocol. Related to Figure 3

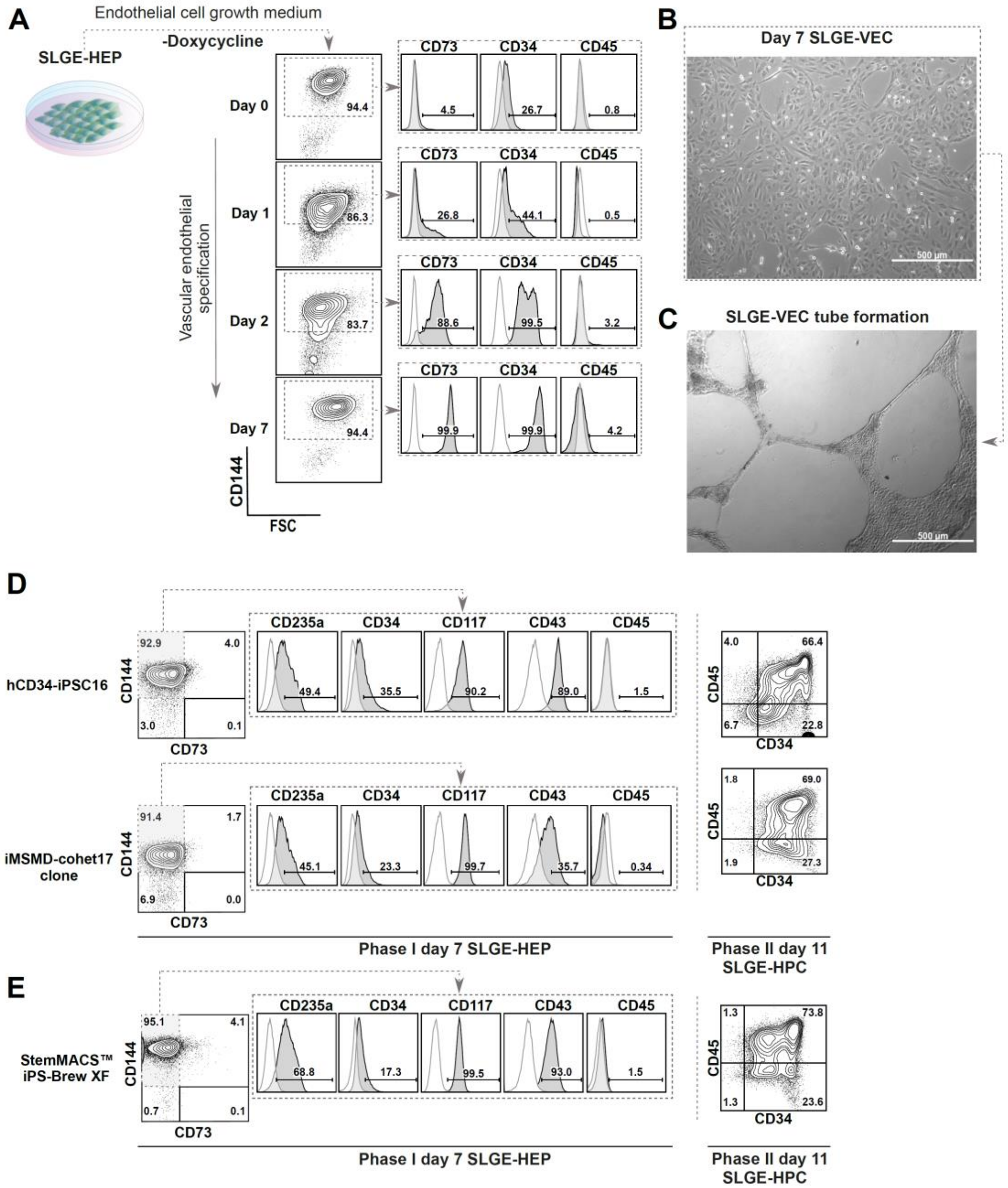

Figure S3 Vascular endothelial potential and reproducibility of the forward programming protocol. Related to Figure 3. (A) Vascular endothelial potential of Phase I day 7 SLGE-HEP. Cells were cultivated for 7 days in EGM2 (endothelial growth medium) without Dox. Kinetic of vascular endothelial specification with upregulation of endothelial-specific surface marker ($CD144^+/CD73^+/CD34^+$) within 7 days (SLGE-VEC) without upregulation of hematopoietic marker CD45. **(B)** Representative microscopy image of SLGE-VEC (7 days in EGM2) (Scale bar 500 µm). **(C)** Tubular-like structures of SLGE-VEC in an *in vitro* angiogenesis assay on gelled basement matrix (Scale bar 500 µm). **(D)** Hemato-endothelial differentiation of different SLGE-iPSC lines. hCD34-iPSC16 iPSC ($CD34^+$ cell-derived iPSC) and iMSMD-cohet 17 iPSC (peripheral blood-derived iPSC). Both lines exhibited similar hemato-endothelial differentiation potential and produced SLGE-HEP (Phase I day 7) as well as SLGE-HPC (Phase II day 11) **(E)** Hemato-endothelial differentiation potential of SLGE-iPSC Phase I day 7 SLGE-HEP and Phase II day 11 SLGE-iPSC cultivated in fully defined xeno-free StemMACS iPS-Brew medium.

Figure S4 Characterization of the HPC potential and function. Related to Figure 5

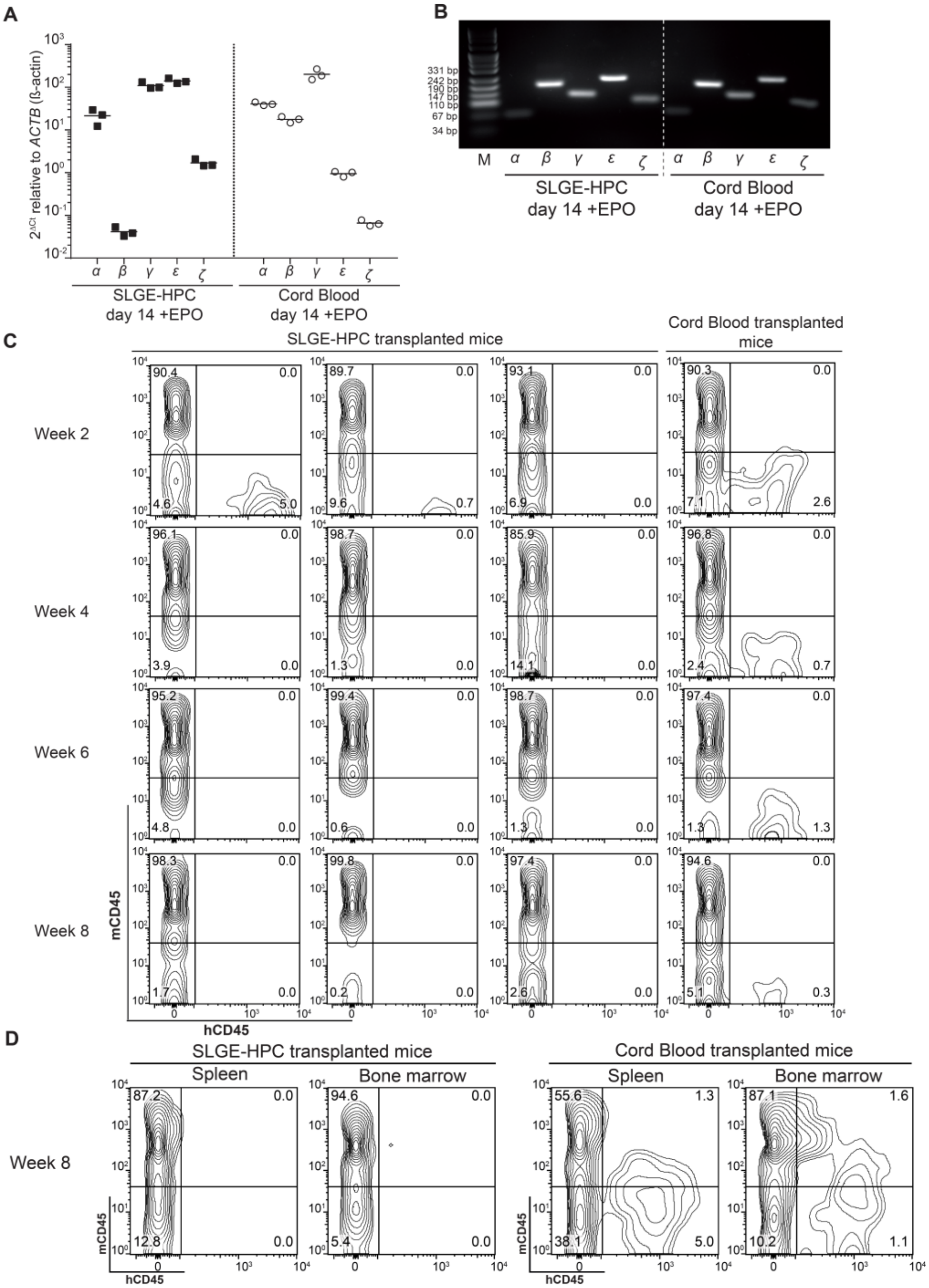

Figure S4 Characterization of the HPC potential and function. Related to Figure 5 (A) Globin Expression profile (embryonic isoform ζ- (*HBZ*) and ε- (*HBE*), the fetal isoform γ- (*HBG*) and α-globin (*HBA*) and the adult type β- (*HBB*)) of SLGE-HPC and cord blood cells (differentiated for 14 day in EPO containing medium) determined by quantitative RT-PCR in triplicate samples. Human *ACTB* (β-actin) was used as housekeeping control and $2^{\Delta CT}$ calculation. **(B)** Gel electrophoresis of the RT-qPCR products of the globin expression profile demonstrated the specificity of the RT-qPCR reactions. **(C)** Analysis of peripheral blood of either cord blood or SLGE-HPC (n=3) transplanted immunodeficient NSGS mice at 2, 4, 6 and 8 weeks post-transplantation for the presence of $hCD45^+$ hematopoietic cells. **(D)** Representative analysis of the spleen or the bone marrow of either cord blood or SLGE-HPC (n=3) transplanted immunodeficient NSGS mice 8 weeks post-transplantation for the presence of $hCD45^+$ cells.

Figure S5 Sorting strategy and gating scheme for the RNA-sequencing approach. Related to Figure 6

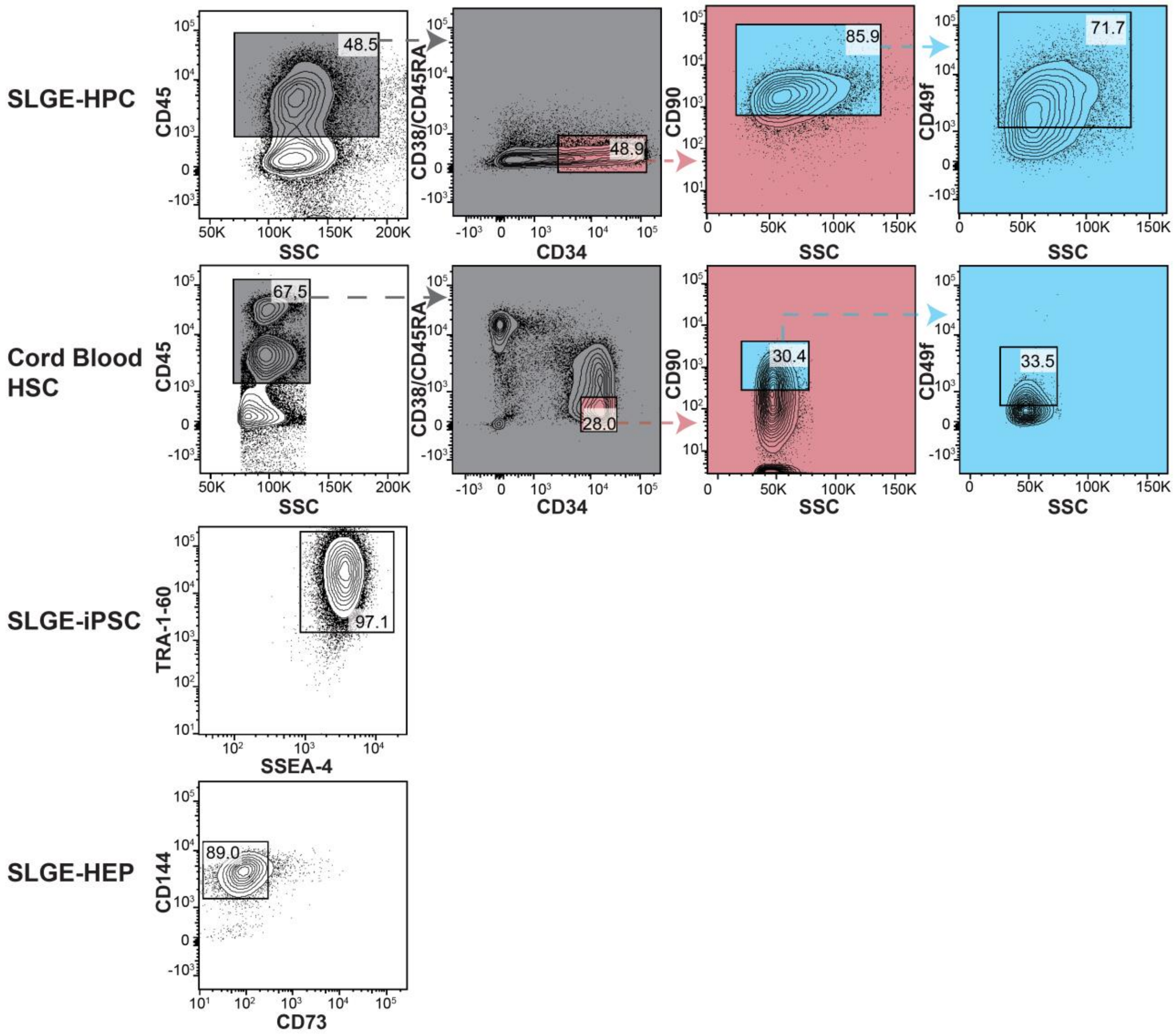

Figure S5 Sorting strategy and gating scheme for the RNA-sequencing approach. Related to Figure 6
Fluorescence activated cell sorting strategy for the RNA-sequencing comparison of SLGE-HPC ($CD45^{+}/CD38^{-}/CD45RA^{-}/CD34^{high}/CD90^{high}/CD49f^{high}$), human umbilical cord blood HSC ($CD45^{+}/CD38^{-}/CD45RA^{-}/CD34^{high}/CD90^{high}/CD49f^{high}$), SLGE-iPSC ($TRA\text{-}1\text{-}60^{+}/SSEA\text{-}4^{+}$) and SLGE-HEP ($CD144^{+}/CD73^{-}$).

Figure S6 RNA-sequencing analysis of SLGE-iPSC derived HEP/HPC and cord blood HSC. Related to Figure 6

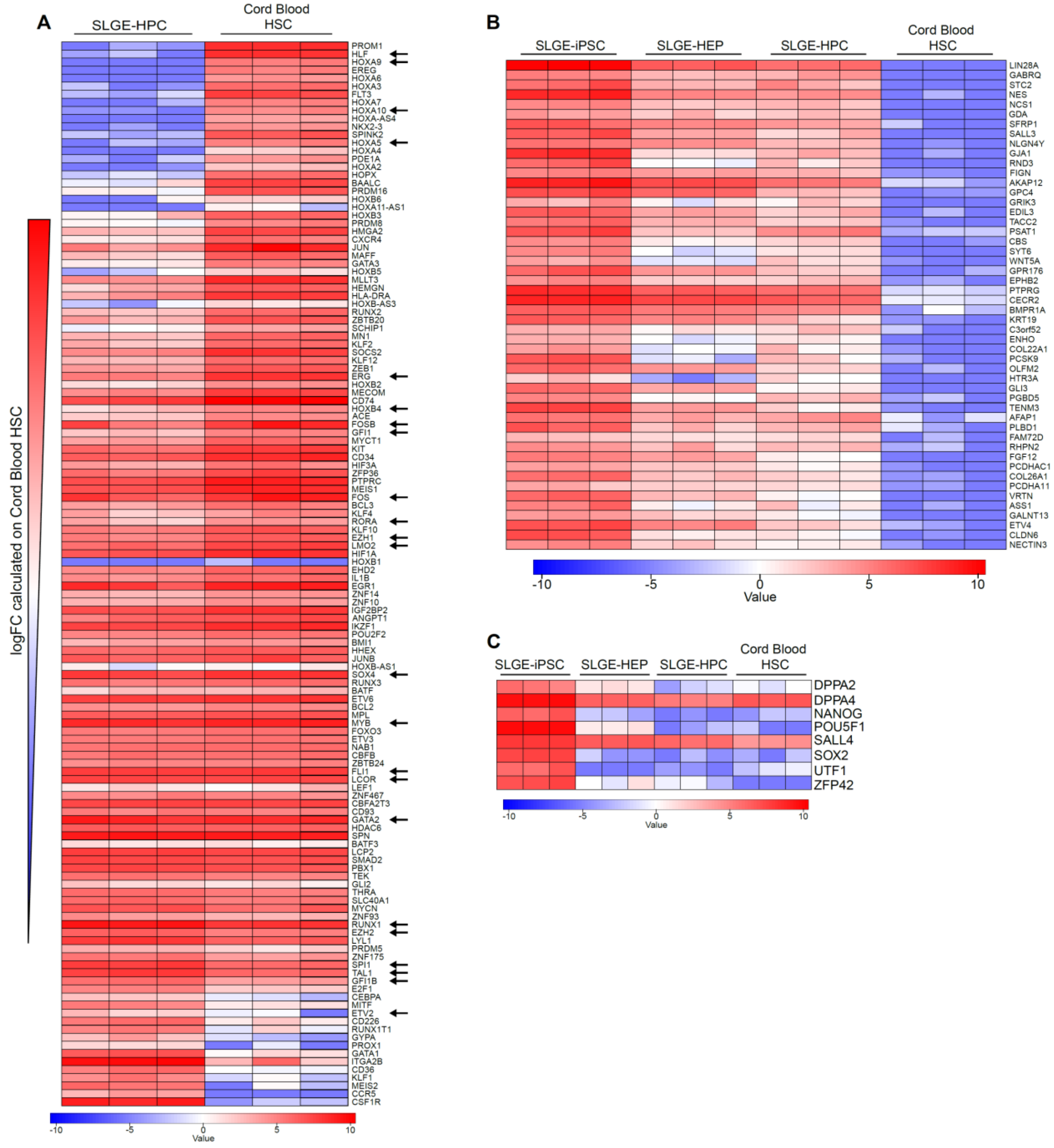

Figure S6 RNA-sequencing analysis of SLGE-iPSC derived HEP/HPC and cord blood HSC. Related to Figure 6 (A) Analysis of differentially or similarly expressed genes associated with hematopoietic development between cord blood HSC and SLGE-HPC. The genes were ranked over the highest logarithmic fold change (logFC) calculated on cord blood HSC. Gene ontology was done using DAVID. Black arrows indicate transcription factors that were previously used in transdifferentiation (Batta et al., 2014; Doulatov et al., 2013; Gomes et al., 2018; Pereira et al., 2013; Riddell et al., 2014; Sandler et al., 2014; Sugimura et al., 2017) and forward programming (Elcheva et al., 2014) approaches. **(B)** Heatmap of log-CPM values for the top 50 of not silenced genes of SLGE-iPSC, SLGE-HEP and SLGE-HPC *(mean* $log_2FC=7.6$*,* $P_{adj} < 0.03$*)* during hemato-endothelial differentiation compared to cord blood HSC. **(C)** Heatmap of log-CPM values for genes associated with pluripotency and stemness.

Supplemental Experimental Procedures

Cell culture

Human iPSC were cultivated in conditioned iPSC medium, composed of Dulbecco's modified Eagle's medium/F12 GlutaMAX with 20% knockout serum replacement (both Gibco, Karlsruhe, Germany), 100 U mL^{-1} penicillin, 100 µg mL^{-1} streptomycin (PAN Biotech), 2 mM L-glutamine (Merck Millipore, Darmstadt, Germany), 1% non-essential amino acids (Gibco), 100 µM β-mercaptoethanol (Sigma-Aldrich) (CM; without bFGF cultured for 24h on γ-irradiated C3H mouse embryonic fibroblasts, harvested, filtered and optionally stored at -20°C) as a feeder-free monolayer (ML) using Geltrex (Gibco) coated cell culture dishes. Fresh FGF2 (30 ng mL^{-1}) was supplemented before usage. Long term iPSC cultivation was performed as ML in CM in the presence of puromycin (0.3 ng mL^{-1}) and/or zeocin (0.5 ng mL^{-1}) (both InvivoGen, Toulouse, France) to prevent vector-silenced SLGE-iPSC throughout the cultivation. Alternatively, iPSC were cultivated and maintained in fully defined StemMACS™ iPS-Brew XF stem cell medium (Miltenyi Biotec, Bergisch Gladbach, Germany). Human SLGE-iPSC were harvested with Accutase (Thermo Fisher, Darmstadt, Germany) and split twice a week at a density of 1×10^6 cells per 9 cm² onto new Geltrex-coated plates. ROCK-inhibitor (Y-27632, 10 µM) (kindly provided by T. Scheper, Leibniz University Hannover) was applied to the SLGE-iPSC culture at least 1h before Accutase treatment and into the fresh iPSC medium.

Lentiviral particle production

Lentiviral particles were produced by calcium phosphate transfection, as previously described (Schambach et al., 2006) and vesicular stomatitis virus envelope glycoprotein (VSV-G) pseudotyped. Lentiviral supernatants were harvested 36 and 48 hours post-transfection, filtered, pooled and concentrated (100-fold) via ultracentrifugation for 2 hours at 82 740 × g (rotor SW32Ti, Beckman Coulter, Krefeld, Germany), 4 °C and stored at – 80°C.

Transduction and antibiotic selection of stable induced pluripotent stem cells

Transductions with the two vectors SL and GE were performed sequentially in suspension to establish double-positive pluripotent stem cell lines (SLGE). Single-cell suspensions of 2.5×10^4 cells were transduced with MOI=0.5 for SL and MOI=10 for GE in 100 µL iPSC-medium for 1h at 37°C and 5% CO_2 in the presence of 4 µg mL^{-1} protamine sulfate (Sigma-Aldrich) and 10 µM Y-27632. Cells were kept in suspension by flicking the tube every 10 minutes and seeded in iPSC medium containing Y-27632 on Geltrex-coated cell culture dishes afterward. Medium was exchanged the following day and every other day. Puromycin (0.3 g mL^{-1}) and zeocin (0.5 µg mL^{-1}) (both InvivoGen) were applied 24 h post-transduction.

Vascular endothelial differentiation of SLGE-HEP

Phase I day 7 SLGE-HEP were cultivated for 7 days in endothelial growth medium 2 (EGM2) (Lonza, Visp, Switzerland) on 0.1% gelatin (Sigma Aldrich) coated cell culture dishes. Flow cytometry was used to analyze the maturation stage over time. Flow cytometry was performed on CytoFLEX S Flow Cytometer (Beckman Coulter) using antibodies against CD144, CD73, CD34 and CD45 (Supplementary flow cytometry antibodies list). Tube-formation assay was done on undiluted Matrigel (Corning) according to previously described method (Arnaoutova and Kleinman, 2010).

Terminal differentiation of hematopoietic progenitor cells

Multipotency and proliferative potential of SLGE-HPC was assessed by colony-forming assay and directed lineage differentiation. For colony-forming assays, 5000 SLGE-HPC were seeded in Methocult H4034 (STEMCELL Technologies) according to the manufacturer's protocol. Colonies were counted and determined using standard morphological criteria after 12 to 14 days. Lineage-specific differentiation of SLGE-HPC into granulocytes was performed in IMDM (Biochrom, Berlin, Germany) supplemented with 10% heat-inactivated fetal bovine serum (FBS) (Pan Biotech), 1% bovine serum albumin (PAA, Coelbe, Germany), 0.5% non-essential amino acids, 2 mM L-glutamine, 100 U mL^{-1} penicillin, 100 µg mL^{-1} streptomycin, 100 µM β-mercaptoethanol, 100 ng mL^{-1} SCF, 25 ng mL^{-1} IL-3 and 50 ng mL^{-1} GM-CSF and 100 ng mL^{-1} G-CSF (all Peprotech). RPMI with 10% heat-inactivated FBS, 100 U mL^{-1} penicillin, 100 µg mL^{-1} streptomycin was either supplemented with 50 ng mL^{-1} M-CSF (Peprotech) for monocyte/macrophage differentiation, with 20 ng mL^{-1} SCF, 25 ng mL^{-1} IL-3 and 3 U mL^{-1} EPO for erythroid differentiation or with 50 ng mL^{-1} TPO (all Peprotech) for megakaryocytic differentiation. T- and NK-cell differentiation was performed in co-cultivation on semi-confluent OP9-Dll1 cells in RPMI supplemented with 20%

FBS, 100 U mL^{-1} penicillin, 100 µg mL^{-1} streptomycin, 10 ng mL^{-1} SCF, 5 ng mL^{-1} Flt3L, 5 ng mL^{-1} IL-7 for T-cell progenitor differentiation and additionally 10 ng mL^{-1} IL-15 (all Peprotech) for NK-specification. To assess morphology of SLGE-iPSC-derived hematopoietic lineages, 5×10^4 cells were spun onto microscope slides using the Cytospin 4 (Thermo Fisher) and stained with May-Grünwald and Giemsa (both Sigma Aldrich), following manufacturer's instructions.

Flow Cytometry and cell sorting

Flow cytometry was performed on BD FACSCalibur (Becton Dickenson, Heidelberg, Germany) or CytoFLEX S Flow Cytometer (Beckman Coulter), sorting was performed on FACSAria Fusion (Becton Dickenson). DAPI (Sigma-Aldrich) was used at a final concentration of 200 ng mL^{-1} as viability dye. Voltage and compensation were adjusted by single stain controls or UltraComp eBeads microspheres (Thermo Fisher). Gating was performed on unstained controls or fluorescence minus one (FMO) as indicated. . All used antibodies can be found in Table S1: Flow Cytometry Antibodies.

Quantitative Reverse-Transcriptase PCR analysis

Total RNA was extracted using Qiagen RNeasy Mini or Micro Kit (Qiagen, Hilden, Germany) according to the manufacturer's protocol. RNase was inhibited using 50 U RiboLock (Thermo Fisher Scientific). cDNA synthesis and removal of genomic DNA were performed using QuantiTect Reverse Transcription Kit (Qiagen) according to the manufacturer's instructions. QuantiTect SYBR® Green RT-PCR Kit (Qiagen) was applied for quantitative PCR using the StepOnePlus Real-Time PCR System (Applied Biosystems, Darmstadt, Germany). To allow the intercomparability of different time points (Figure 4A), the point of maximum gene expression was set to one for each target. All used primers can be found in Table S2: Primer/Oligonucleotides. Expression of target genes was evaluated as ΔΔCt relative to expression of a housekeeper gene (*ACTB* (β-Actin))

Isolation of human CD34$^+$ cells from umbilical cord blood

Human cord blood was obtained from the Department of Gynecology and Obstetrics at Hannover Medical School with written informed consent, according to the local ethics committee. Mononuclear cells were isolated with Leucosep separation tubes (Greiner Bio-One, Frickenhausen, Germany) according to the manufacturer's protocol using biocoll as separation solution (Biochrom). CD34$^+$ selection was done using the CD34 MicroBead Kit (Miltenyi) according to the manufacturer's instructions. Positively selected cells were either directly sorted or cryostored in StemSpan (STEMCELL Technologies) with 10% DMSO.

Gene expression profiling by RNA-Sequencing

Whole transcriptome analysis was done comparing SLGE-iPSC (TRA-1-60$^+$/SSEA4$^+$), SLGE-HEP (CD144$^+$/CD73$^-$, 3 independent differentiations), SLGE-HPC (CD45$^+$/CD34$^+$/CD90$^+$/CD49f$^+$/CD38$^-$/CD45RA$^-$; 3 independent differentiations) with human umbilical cord blood HSC (CD45$^+$/CD34$^+$/CD90$^+$/CD49f$^+$/CD38$^-$/CD45RA$^-$ from 3 individual preparations and sorts). All samples used for RNA-sequencing were FACS-sorted in line with a previously described sorting strategy (Notta et al., 2015) (Figure S5) All used antibodies can be found in the supplemental information in the flow cytometry antibodies list. DAPI (Sigma-Aldrich) was used at a final concentration of 200 ng mL^{-1} as viability dye. Total RNA was isolated using Qiagen RNeasy Micro Kit (Qiagen). RNA quality/integrity was assessed using an Agilent 2100 Bioanalyzer (Agilent, Waldbronn, Germany). Prior to RNA-sequencing, ribosomal RNA was removed using a NEBNext rRNA Depletion Kit (New England BioLabs, Frankfurt am Main, Germany), according to the manufacturer's protocol. 5 ng total RNA were used for library preparation with the 'SMARTer Stranded Total RNA-Seq Kit v2 – Pico Input Mammalian' (#634413; Takara, Saint-Germain-en-Laye, France) according to conditions recommended in user manual #063017. Generated libraries were barcoded by a dual indexing approach and were amplified by 11 PCR cycles. Fragment length distribution of generated libraries was monitored using 'Bioanalyzer High Sensitivity DNA Assay' (5067-4626; Agilent Technologies). Quantification of libraries was performed with the 'Qubit® dsDNA HS Assay Kit' (Q32854; ThermoFisher Scientific). Sequencing runs were performed with equal molar amounts of five (SLGE-HPC and Cord Blood HSC), or six (SLGE-iPSC and SLGE-HEP) pooled libraries for a common sequencing run. Accordingly, each analyzed library constitutes 20% (SLGE-HPC and Cord Blood HSC), or 16.6% (SLGE-iPSC and SLGE-HEP) of overall flowcell capacity. The combined library pools were denatured with sodium hydroxide and were finally diluted to 1.5 pM according to the Denature and Dilute Libraries Guide (Document # 15048776 v02; Illumina, San Diego, USA). 1.3 mL of each denatured pool was loaded on an Illumina NextSeq 550 sequencer using a High Output

Flowcell for 75 bp single reads (#FC-404-2005, Illumina). Raw data were processed by converting the BCL files into FASTQ files using bcl2fastq Conversion Software version v2.20.0.422 (Illumina). The FASTQ files were adapted and quality trimmed using Trim Galore! (Version 0.4.1) with default settings as described in the User Guide except for the setting of the quality cutoff (-q/--quality), which was set to a Phred score of 15. Trim Galore! used Cutadapt (version 1.9.1) as subroutine. Quality control of FASTQ files was performed by FastQC (version 0.11.4) before and after trimming. After trimming, FASTQ files were mapped against a reference genome with the splice-aware aligner STAR (version 2.5.0c) to generate BAM files. The BAM files were built in a 2-pass mapping (--twopassMode Basic) and were finally sorted (--outSAMtype BAM SortedByCoordinate). All other settings were left as default as described in the manual. The genome index files were created by STAR with default settings using *Homo sapiens* sequence and annotation data (UCSC, built hg19) available on Illumina's iGenome site (http://support.illumina.com/sequencing/sequencing_software/igenome.html). The average number of reads entering the mapping process across all analyzed samples was 94.4 million (SLGE-HPC and Cord Blood HSC) or 90.9 million (SLGE-iPSC and SLGE-HEP). The average percentage of uniquely mapped reads was 79.9% (SLGE-HPC and Cord Blood HSC) or 78.7% (SLGE-iPSC and SLGE-HEP). The average percentage of reads mapped to multiple loci was 5.5% (SLGE-HPC and Cord Blood HSC), or 7.1% (SLGE-iPSC and SLGE-HEP).The average percentage of unmapped reads was 14.4% (SLGE-HPC and Cord Blood HSC) or 13.8% (SLGE-iPSC and SLGE-HEP). Read counting was performed by featureCounts as part of the Subread (Liao et al., 2013) software package (version 1.6.1) with default settings except for strandedness (-s), which was set to 2, i.e. reverse, minimum mapping quality per read (-Q) was set to 10, counting primary alignments only (--primary) and counting multi-mapping reads was enabled (-M) together with fractional assignment of reads (--fractional). *Homo sapiens* annotation data (gencode.v28lift37) was used. Raw counts were filtered (counts per million (cpm) >0.6 in more than three samples, 22,214 genes after filtering) and TMM-normalized using edgeR (McCarthy et al., 2012). For visualization (PCA and heatmaps) filtered and normalized counts were transformed into log-cpm values using a prior count of 1. Differential expression analysis was performed using limma-voom (Law et al., 2014). P-values were adjusted for multiple testing using the Benjamini-Hochberg adjustment method in the limma package (Ritchie et al., 2015). Heatmaps were generated with the function heatmap.2 from the gplots package on the log-cpm values on the features/genes indicated in the figure legends. Principal component analysis (PCA, Figure 6A) was performed on log-CPM values for all 22,214 genes. For Gene Set Enrichment analysis gene-lists for the contrasts of interest (CB-HSC vs SLGE-HPC) were generated using the limma "topTable" function. This assigns to each of the 22,214 unique genes a log-FC and a p-value for the given contrast. The gene-lists were sorted by decreasing log-FC, filtered for protein-coding genes using the GenCode V28 annotation and submitted to the Broad GSEA tool (Subramanian et al., 2005) using the GSEA pre-ranked function with the gene set permutations set to 1000. In addition to the gene set collections from MSigDB v6.2 (Subramanian et al., 2005), 130 hematopoiesis-related gene sets were tested (Schwarzer et al., 2017).

Mouse transplantation and teratoma assay

All animal experiments were approved by the animal welfare committee of Lower Saxony and were performed in accordance with institutional guidelines of Hannover Medical School. Mice were bred and housed in pathogen-free environment in the animal facility of Hannover Medical School. Transplantations were conducted by intravenous tail-vein injection into 5 weeks old NSGS mice (NOD.Cg-Prkdcscid Il2rgtm1Wjl Tg(CMV-IL3,CSF2,KITLG)1Eav/MloySzJ). Before transplantation, mice were sublethally irradiated with 1.9 Gy using GammaCell2000 (Cs_{137}) (Nuklear Data, Frankfurt, Germany). Transplantations were conducted either with SLGE-HPC (1.7×10^6 cells/mice in 150 µL total injection volume) or expanded human $CD34^+$ cord blood cells (8×10^5 cells/mice in 150 µL total injection volume) as positive control. Expansion of cord blood-derived $CD34^+$ cells was performed in StemSpan (STEMCELL Technologies), supplemented with 100 ng ml^{-1} SCF, 50 ng ml^{-1} TPO, 100 ng ml^{-1} Flt3L, 35 nM UM171 (ApexBio, Houston, USA), 500 nM StemReginin 1 (STEMCELL Technologies). Blood sampling was performed by puncture of retro-orbital plexus 2, 4, 6 and 8 weeks post-transplantation. Total chimerism was analyzed 8 weeks post-transplantation in peripheral blood, spleen and bone marrow by flow cytometry with antibodies against human CD45-APC (clone: 2D1, Thermo Fisher) and anti-murine-CD45-PE (Clone 30F11, Miltenyi Biotec). For teratoma formation, H2E6C SLGE-iPSC were harvested in the presence of 10 µM Y-27632 by TrypLE Select (Thermo Fisher Scientific) treatment. Cell suspension was mixed 1:1 with undiluted Matrigel (Corning, Kaiserslautern, Germany). 3×10^6 SLGE-iPSC in 200 µL were subcutaneously injected into each flank of adult NSGS mice, as described previously (Philipp et al., 2018). After tumor formation (41 days), teratomas were fixed in 4% formaldehyde (Carl Roth, Karlsruhe, Germany) and embedded in paraffin. 3 µm thick tissue slices were stained with hematoxylin and eosin according to standard protocols. Determination of germ layers was accomplished with an Olympus system (microscope BX51, camera XC50, software Cell^F 3.4).

Statistics

All statistical analyses were done using GraphPad Prism. Two-tailed, unpaired *t*-test (normal distribution assumption) was used for comparison between two groups. One-way ANOVA was used for statistical comparison for more than two groups, as indicated. P-values > 0.05 were considered not significant (ns). P-values ≤ 0.05 (*) were considered significant, p-values ≤ 0.01 (**) were considered very significant, and p-values ≤ 0.001 (***) were considered extremely significant.

Table S1: Flow Cytometry Antibodies

Antibody	Fluorophore	Reactivity	Dilution	Used in Figure	Supplier	Cat.No.
**Anti SSEA-4**	Alexa Fluor 647	Human	1:25	S1E/S5	BD Bioscience	560796
**Anti Tra-1-60**	PE	Human	1:25	S1E/S5	Miltenyi Biotec	130-100-347
**CD117**	PE	Human	1:25	2F/S3D/S3E	Miltenyi Biotec	130-091-734
**CD11b**	PE	Human+Mouse	1:25	5A	Miltenyi Biotec	130-091-240
**CD14**	Alexa Fluor 700	Human	1:25	5A	Biolegend	367114
**CD144**	FITC	Human	1:25	1B/1C/2A/2F/3B/S2C/S3A/S3D/S3E/S5	BD Bioscience	560411
**CD15**	FITC	Human	1:25	5A	Miltenyi Biotec	130-098-013
**CD16**	PE-Vio770	Human	1:25	5A	Miltenyi Biotec	130-096-655
**CD163**	PerCP	Human	1:25	5A	BioLegend	333626
**CD209**	PE-Cy7	Human	1:25	5A	BioLegend	330114
**CD235a**	APC-C7	Human	1:25	2F/S2B/3E/S3D/S3E/5A	Biolegend	349116
**CD309**	PE	Human	1:25	S2A	BD Bioscience	560494
**CD34**	PE-Cy7	Human	1:25	2F/S3A/S3D/S3E	BD Bioscience	348811
**CD34**	APC	Human	1:25	3B/3E/S5	BD Bioscience	345804
**CD38**	FITC	Human	1:25	3E/S5	eBioscience	11-0388-42
**CD41a**	APC	Human	1:25	5A	Miltenyi Biotec	130-105-613
**CD42b**	PE	Human	1:25	5A	Biolegend	303906
**CD43**	PerCp-eFluor710	Human	1:25	2F/S2B/3E/S3D/S3E	eBioscience	46-0438-42
**CD45**	BV650	Human	1:25	2F/5A/3E/S3A/S3D/S3E/S5	BD Bioscience	563717
**CD45**	PE	Human	1:25	3B	Miltenyi Biotec	130-080-201
**CD45**	APC	Human	1:25	S4C	eBioscience	17-9459-42
**CD45**	PE	Mouse	1:25	S4C	Miltenyi Biotec	130-117-348
**CD45RA**	FITC	Human	1:25	3E/S5	Miltenyi Biotec	130-108-713
**CD49f**	PE	Human+Mouse	1:100	3E/S5	BioLegend	313612
**CD56**	PE	Human	1:25	5A	BD Bioscience	555516
**CD61**	PerCP	Human	1:25	5A	BD Bioscience	564173
**CD66b**	PE	Human	1:25	5A	BD Bioscience	561650
**CD73**	APC	Human	1:25	1B/1C/2A/2F/S2C/S3A	Miltenyi Biotec	130-097-945
**CD73**	PE	Human	1:50	S5	Miltenyi Biotec	130-095-182
**CD86**	APC	Human	1:25	5A	Biolegend	305412
**CD90**	PE-Cy7	Human	1:25	3E/S5	Biolegend	328123

Table S2: Primer/Oligonucleotides

Primer ID	Primer sequences 5' → 3'	Amplicon length	Used in Figure	Reference
OCT4 Forward<br>*OCT4* Reverse	CCTCACTTCACTGCACTGTA<br>CAGGTTTTCTTTCCCTAGCT	164 bp	1B/4A	(Schott et al., 2014)
NANOG Forward<br>*NANOG* Reverse	TCACACGGAGACTGTCTCTC<br>GAACACAGTTCTGGTCTTCTG	169 bp	1B	(Schott et al., 2014)
β-ACTIN Forward<br>*β-ACTIN* Reverse	CCTCCCTGGAGAAGAGCTA<br>TCCATGCCCAGGAAGGAAG	109 bp	1B/1D/4A/4B/S4A	(Galla et al., 2011)
exo *SCL* Forward<br>exo *SCL* Reverse	GACAGCTACACGGAGGAGC<br>GCCACAGGTCAGCAGAGAG	126 bp	1D/4B	-
exo *LMO2* Forward<br>exo *LMO2* Reverse	GCTCTCTGCTGACCTGTGG<br>GGGTCCAGGCTCTTCCTTTC	82 bp	1D/4B	-
exo *GATA2* Forward<br>exo *GATA2* Reverse	TCCTCCAGCCTCTCCTTCG<br>GCTTCAACAGGGCGTAGTTTGT	91 bp	1D/4B	-
exo *ETV2co* Forward<br>exo *ETV2co* Reverse	TGGACCGATATGGCCTGTA<br>GATCCAGCGGCAGGAATAG	101 bp	1D/4B	-
TBXT Forward<br>*TBXT* Reverse	TGAGCCTCGAATCCACATAGTG<br>AAGAGCTGTGATCTCCTCGT	121 bp	4A	-
KDR Forward<br>*KDR* Reverse	AGCAGGATGGCAAAGACTAC<br>TACTTCCTCCTCCTCCATACAG	116 bp	4A	(Gao et al., 2017)
RUNX1 Forward<br>*RUNX1* Reverse	ACTCGGCTGAGCTGAGAAATG<br>GACTTGCGGTGGGTTTGTG	140 bp	4A	(Challen and Goodell, 2010)
RUNX1c Forward<br>*RUNX1c* Reverse	TGGTTTTCGCTCCGAAGGT<br>CATGAAGCACTGTGGGTACGA	130 bp	4A	(Challen and Goodell, 2010)
CDH5 Forward<br>*CDH5* Reverse	GCAGCAGCAGGTGCTAACC<br>TTGCCCACATATTCTCCTTTG	203 bp	4A	(Zambidis et al., 2005)
HBB Forward<br>*HBB* Reverse	AGGAGAAGTCTGCCGTTACTG<br>CCGAGCACTTTCTTGCCATGA	190 bp	S4A/B	(Dou et al., 2016)
HBE Forward<br>*HBE* Reverse	CTTTGGAAACCTGTCGTC<br>CTTGCCAAAGTGAGTAGC	227 bp	S4A/B	(Dou et al., 2016)
HBG Forward<br>*HBG* Reverse	GGGAGATGCCATAAAGC<br>ATTGCCAAAACGGTCAC	135 bp	S4A/B	(Dou et al., 2016)
HBZ Forward<br>*HBZ* Reverse	GTGTCCATGTGGGCCAAG<br>GAAGTGCGGGAAGTAGGTCTT	108 bp	S4A/B	(Fujita et al., 2016)
HBA Forward<br>*HBA* Reverse	TCCCCACCACCAAGACCTA<br>CCTTAACCTGGGCAGAGCC	63bp	S4A/B	(Fujita et al., 2016)

Supplemental References

Arnaoutova, I., and Kleinman, H.K. (2010). In vitro angiogenesis: endothelial cell tube formation on gelled basement membrane extract. Nat. Protoc. *5*, 628–635.

Batta, K., Florkowska, M., Kouskoff, V., and Lacaud, G. (2014). Direct Reprogramming of Murine Fibroblasts to Hematopoietic Progenitor Cells. Cell Rep. *9*, 1871–1885.

Challen, G.A., and Goodell, M.A. (2010). Runx1 isoforms show differential expression patterns during hematopoietic development but have similar functional effects in adult hematopoietic stem cells. Exp. Hematol. *38*, 403–416.

Dou, D.R., Calvanese, V., Sierra, M.I., Nguyen, A.T., Minasian, A., Saarikoski, P., Sasidharan, R., Ramirez, C.M., Zack, J.A., Crooks, G.M., et al. (2016). Medial HOXA genes demarcate haematopoietic stem cell fate during human development. Nat. Cell Biol. *18*, 595–606.

Doulatov, S., Vo, L.T.T., Chou, S.S.S., Kim, P.G.G., Arora, N., Li, H., Hadland, B.K.K., Bernstein, I.D.D., Collins, J.J.J., Zon, L.I.I., et al. (2013). Induction of Multipotential Hematopoietic Progenitors from Human Pluripotent Stem Cells via Respecification of Lineage-Restricted Precursors. Cell Stem Cell *13*, 459–470.

Elcheva, I., Brok-Volchanskaya, V., Kumar, A., Liu, P., Lee, J.H., Tong, L., Vodyanik, M., Swanson, S., Stewart, R., Kyba, M., et al. (2014). Direct induction of haematoendothelial programs in human pluripotent stem cells by transcriptional regulators. Nat. Commun. *5*, 1–11.

Fujita, A., Uchida, N., Haro-Mora, J.J., Winkler, T., and Tisdale, J. (2016). β-Globin-Expressing Definitive Erythroid Progenitor Cells Generated from Embryonic and Induced Pluripotent Stem Cell-Derived Sacs. Stem Cells *34*, 1541–1552.

Galla, M., Schambach, A., Falk, C.S., Maetzig, T., Kuehle, J., Lange, K., Zychlinski, D., Heinz, N., Brugman, M.H., Göhring, G., et al. (2011). Avoiding cytotoxicity of transposases by dose-controlled mRNA delivery. Nucleic Acids Res. *39*, 7147–7160.

Gao, S., Li, C., Zhu, Y., Wang, Y., Sui, A., Zhong, Y., Xie, B., and Shen, X. (2017). PEDF mediates pathological neovascularization by regulating macrophage recruitment and polarization in the mouse model of oxygen-induced retinopathy. Sci. Rep. *7*, 42846.

Gomes, A.M., Kurochkin, I., Chang, B., Daniel, M., Law, K., Satija, N., Lachmann, A., Wang, Z., Ferreira, L., Ma'ayan, A., et al. (2018). Cooperative Transcription Factor Induction Mediates Hemogenic Reprogramming. Cell Rep. *25*, 2821-2835.e7.

Law, C.W., Chen, Y., Shi, W., and Smyth, G.K. (2014). voom: precision weights unlock linear model analysis tools for RNA-seq read counts.

Liao, Y., Smyth, G.K., and Shi, W. (2013). The Subread aligner: Fast, accurate and scalable read mapping by seed-and-vote. Nucleic Acids Res. *41*.

McCarthy, D.J., Chen, Y., and Smyth, G.K. (2012). Differential expression analysis of multifactor RNA-Seq experiments with respect to biological variation. Nucleic Acids Res. *40*, 4288–4297.

Notta, F., Zandi, S., Takayama, N., Dobson, S., Gan, O.I., Wilson, G., Kaufmann, K.B., Mcleod, J., Laurenti, E., Dunant, C.F., et al. (2015). Distinct routes of lineage development reshape the human blood hierarchy across ontogeny. Science *351*, 1–16.

Pereira, C.F., Chang, B., Qiu, J., Niu, X., Papatsenko, D., Hendry, C.E., Clark, N.R., Nomura-Kitabayashi, A., Kovacic, J.C., Ma'Ayan, A., et al. (2013). Induction of a hemogenic program in mouse fibroblasts. Cell Stem Cell *13*, 205–218.

Philipp, F., Selich, A., Rothe, M., Hoffmann, D., Rittinghausen, S., Morgan, M.A., Klatt, D., Glage, S., Lienenklaus, S., Neuhaus, V., et al. (2018). Human Teratoma-Derived Hematopoiesis Is a Highly Polyclonal Process Supported by Human Umbilical Vein Endothelial Cells. Stem Cell Reports *11*, 1051–1060.

Riddell, J., Gazit, R., Garrison, B.S., Guo, G., Saadatpour, A., Mandal, P.K., Ebina, W., Volchkov, P., Yuan, G.-C., Orkin, S.H., et al. (2014). Reprogramming committed murine blood cells to induced hematopoietic stem cells with defined factors. Cell *157*, 549–564.

Ritchie, M.E., Phipson, B., Wu, D., Hu, Y., Law, C.W., Shi, W., and Smyth, G.K. (2015). limma powers differential expression analyses for RNA-sequencing and microarray studies. Nucleic Acids Res. *43*.

Sandler, V.M., Lis, R., Liu, Y., Kedem, A., James, D., Elemento, O., Butler, J.M., Scandura, J.M., and Rafii, S. (2014). Reprogramming human endothelial cells to haematopoietic cells requires vascular induction. Nature.

Schambach, A., Bohne, J., Chandra, S., Will, E., Margison, G.P., Williams, D.A., and Baum, C. (2006). Equal potency of gammaretroviral and lentiviral SIN vectors for expression of O6-methylguanine-DNA methyltransferase in hematopoietic cells. Mol. Ther. *13*, 391–400.

Schott, J.W., Hoffmann, D., Maetzig, T., Müller, F.J., Steinemann, D., Zychlinski, D., Cantz, T., Baum, C., and Schambach, A. (2014). Improved retroviral episome transfer of transcription factors enables sustained cell fate modification. Gene Ther. *21*, 938–949.

Schwarzer, A., Emmrich, S., Schmidt, F., Beck, D., Ng, M., Reimer, C., Adams, F.F., Grasedieck, S., Witte, D., Käbler, S., et al. (2017). The non-coding RNA landscape of human hematopoiesis and leukemia. Nat. Commun. *8*, 218.

Subramanian, A., Tamayo, P., Mootha, V.K., Mukherjee, S., Ebert, B.L., Gillette, M.A., Paulovich, A., Pomeroy, S.L., Golub, T.R., Lander, E.S., et al. (2005). Gene set enrichment analysis: a knowledge-based approach for interpreting genome-wide expression profiles. Proc. Natl. Acad. Sci. U. S. A. *102*, 15545–15550.

Sugimura, R., Jha, D.K., Han, A., Soria-Valles, C., da Rocha, E.L., Lu, Y.-F., Goettel, J.A., Serrao, E., Rowe, R.G., Malleshaiah, M., et al. (2017). Haematopoietic stem and progenitor cells from human pluripotent stem cells. Nature.

Zambidis, E.T., Peault, B., Park, T.S., Bunz, F., and Civin, C.I. (2005). Hematopoietic differentiation of human embryonic stem cells progresses through sequential hematoendothelial, primitive, and definitive stages resembling human yolk sac development. Blood *106*, 860–870.
