# Supplementary material for: Inducible Forward Programming of Human Pluripotent Stem Cells to Hemato-endothelial Progenitor Cells with Hematopoietic Progenitor Potential
Source: Stem Cell Reports. 2019 Dec 12;14(1):122–37. doi: 10.1016/j.stemcr.2019.11.005 (PMC6962646; doi:10.1016/j.stemcr.2019.11.005)
Supplement: Document S2. Article plus Supplemental Information [file mmc3.pdf]

Stem Cell Reports
Resource
OPEN ACCESS

Inducible Forward Programming of Human Pluripotent Stem Cells to Hemato-endothelial Progenitor Cells with Hematopoietic Progenitor Potential

Lucas Lange,[1,2] Dirk Hoffmann,[1,2] Adrian Schwarzer,[1,2,3] Teng-Cheong Ha,[1,2] Friederike Philipp,[1,2,4] Daniela Lenz,[1,2] Michael Morgan,[1,2] and Axel Schambach[1,2,5,*]
[1]Institute of Experimental Hematology, Hannover Medical School, Carl-Neuberg-Strasse 1, 30625 Hannover, Germany
[2]REBIRTH Cluster of Excellence, Hannover Medical School, 30625 Hannover, Germany
[3]Department of Hematology, Oncology, Hemostasis and Stem Cell Transplantation, Hannover Medical School, Hannover 30625, Germany
[4]Fraunhofer Institute for Toxicology and Experimental Medicine, 30625 Hannover, Germany
[5]Division of Hematology/Oncology, Boston Children's Hospital, Harvard Medical School, 02115 Boston, MA, USA
*Correspondence: schambach.axel@mh-hannover.de
https://doi.org/10.1016/j.stemcr.2019.11.005

SUMMARY

Induced pluripotent stem cells (iPSCs) offer a promising platform to model early embryonic developmental processes, to create disease models that can be evaluated by drug screens as well as proof-of-concept experiments for regenerative medicine. However, generation of iPSC-derived hemato-endothelial and hematopoietic progenitor cells for these applications is challenging due to variable and limited cell numbers, which necessitates enormous up-scaling or development of demanding protocols. Here, we unravel the function of key transcriptional regulators *SCL*, *LMO2*, *GATA2*, and *ETV2* (SLGE) on early hemato-endothelial specification and establish a fully inducible and stepwise hemato-endothelial forward programming system based on SLGE-regulated overexpression. Regulated induction of SLGE in stable SLGE-iPSC lines drives very efficient generation of large numbers of hemato-endothelial progenitor cells (CD144^{+}/CD73^{-}), which produce hematopoietic progenitor cells (CD45^{+}/CD34^{+}/CD38^{-}/CD45RA^{-}/CD90^{+}/CD49f^{+}) through a gradual process of endothelial-to-hematopoietic transition (EHT).

INTRODUCTION

Primitive hematopoiesis is the first (transient) wave of emerging hematopoietic cells during embryonic development and generates predominantly primitive erythroid cells, macrophages and megakaryocytes within the blood islands in the yolk sac (Palis et al., 1999). Hematopoietic cells, including definitive hematopoietic stem cells (HSCs), with multi-lineage potential and capacity to engraft and reconstitute the blood system, emerge in the definitive wave of hematopoiesis, and originate from different sites within the embryo proper, including the ventral wall of the dorsal aorta within the aorta-gonad-mesonephros region (de Bruijn et al., 2002; Medvinsky and Dzierzak, 1996). Expression of endothelial markers on early hematopoietic cells demonstrates a direct link of hematopoietic and endothelial cells and a common endothelial progenitor (Bertrand et al., 2005; de Bruijn et al., 2002). Lineage-tracing studies provided further evidence for a specialized hemato-endothelial progenitor cell population (Zovein et al., 2009) that was broadly termed hemogenic endothelium (HE). HE originates from mesodermal precursors with functional heterogeneity and distinct primitive and definitive hematopoietic capacities (Choi et al., 2012; D'Souza et al., 2018; Sturgeon et al., 2014). HE gives rise to hematopoietic cells through a process called endothelial-to-hematopoietic transition (EHT) (Eilken et al., 2009). During EHT, endothelial cells successively lose endothelial signature and acquire the hematopoietic phenotype. These processes are strongly controlled by expression of transcription factors (TFs), which direct key stages of hematopoietic development. Among others, members of the ETS (E-twenty-six) TF family are key regulators in the network that governs hematopoiesis. One of the most important members is Ets-related protein 71 (*Er71*; *Etv2*) (Lee et al., 2008), which promotes mesodermal formation (Rasmussen et al., 2011) and hematopoietic development. In addition, *Scl* (*Tal1*) is crucial for development of all hematopoietic lineages in the mouse (Porcher et al., 1996) but also indispensable for the establishment of the HE (Lancrin et al., 2009). However, processes of human embryonic hematopoiesis are still poorly understood and mostly extrapolated from animal models. Thus, the use of human pluripotent stem cells (PSCs) and human induced pluripotent stem cells (hiPSCs), offer a powerful tool for new attractive modeling systems to mimic human ontogenetic processes *in vitro*. Directed differentiation protocols mimic ontogenetic processes by co-cultivation systems and/or addition of morphogens (Choi et al., 2012; Kennedy et al., 2012) at the expense of technically demanding protocols, which can generate multipotent hematopoietic progenitor cells (HPCs) capable of producing myeloid and, to a lesser extent, lymphoid hematopoietic cells. However, *de novo* generation of engraftable hematopoietic stem and progenitor cells (HSPCs) from PSCs produced with these protocols has not

 © 2019 The Author(s).
This is an open access article under the CC BY license (http://creativecommons.org/licenses/by/4.0/).

been convincingly demonstrated. As alternative approaches, several groups overexpressed single or combinations of key TFs in PSC to further improve efficacy and hematopoietic differentiation capacity (Blaser and Zon, 2018). For example, PSCs were directly converted into endothelial cells with a restricted, pan-myeloid or erythro-megakaryocytic, potential (Elcheva et al., 2014), or the hematopoietic capacity of HE to produce HSPCs was improved but only in immunodeficient mice *in vivo* (Sugimura et al., 2017). Although multiple protocols demonstrated the feasibility of hPSC differentiation toward HE and HPCs, hematopoietic *in vitro* differentiation remains challenging. This is likely due to the complexity of hematopoietic ontogeny and, as a consequence, demanding differentiation protocols. Moreover, the challenge to generate HPCs and, especially, HSPCs from iPSCs is already apparent in early stages of hematopoietic differentiation, with the generation of adequate hemato-endothelial progenitor (HEP) cells. HEPs often represent the minority of heterogeneous differentiation cultures and must be purified before hematopoietic specification and yet the yield of HEPs and consequently HPCs limits the use of iPSC technologies for several complex experimental settings.

In this study, we aimed to establish a defined, efficient, and stepwise hemato-endothelial specification protocol starting from iPSCs. This protocol is based on a combinatorial approach of directed differentiation and inducible, TF-mediated forward programming. In a gain-of-function approach, we unraveled the effect of selected key TFs on hemato-endothelial specification. We further utilized the induced TF expression of the best-performing combination (*SCL*, *LMO2*, *GATA2*, and *ETV2*) in a defined time window to produce large numbers of HEPs, which gave rise to HPCs that were able to generate erythro-megakaryocytes, granulocytes, monocytes/macrophages, and natural killer (NK) cells. Interestingly, our robust protocol did not directly convert iPSCs into HEPs but followed key steps of mesodermal specification, endothelial priming, and a gradual EHT. Thus, our differentiation method mirrors main physiologic hematopoietic processes *in vitro* and will be useful to identify further master regulators of early human hematopoiesis. Moreover, the large amount of *in vitro* generated HEPs, HPCs, and mature hematopoietic cells provides an experimental basis to use this system for disease modeling, drug discovery/screening experiments, and identification of gene regulatory networks.

RESULTS

Identification of TF Combinations for Hemato-endothelial Specification of Human iPSCs

Several TFs have been described as master regulators of mesodermal patterning, including both endothelial and hematopoietic development (Batta et al., 2014; Elcheva et al., 2014; Lancrin et al., 2009; Liu et al., 2015; Pereira et al., 2013; Zhou et al., 2019). With the aim to establish an inducible and stepwise differentiation protocol to unravel the individual and combinatorial effects of TFs on early hemato-endothelial specification starting from human iPSCs, we analyzed the capacity of four master regulators, namely *SCL*, *LMO2*, *GATA2*, and *ETV2* (SLGE), to generate HEPs (Figure 1A). The abovementioned TFs were cloned in mono- or bicistronic configurations via 2A-peptide sequences into third-generation doxycycline (Dox)-inducible all-in-one self-inactivating (SIN) lentiviral vectors. The TF cassettes were driven by the improved T11 Tet-responsive promoter element for tightly regulated transgene expression (Heinz et al., 2011) (Figure 1A). The Dox-dependent transactivator rtTA2S-M2 (M2) was constitutively expressed by the human phosphoglycerate kinase promoter. To allow continuous selection against vector/promoter silencing in SLGE-iPSC cultures, M2 was fused via a 2A-peptide sequence to an antibiotic resistance gene (puromycin or zeocin). The resulting vectors (Figure 1A) were packaged into lentiviral particles and titrated on a self-designed HT1080 reporter cell line for this Dox-inducible vector system (Figure S1A). Vector titers ranged from 7×10^7 to 4×10^8 transducing units/mL (Figure S1B) and were used to transduce a previously described human fibroblast-derived iPSC line (H2E6C) (Hoffmann et al., 2017). We generated 15 different, genetically modified, stable iPSC lines harboring an inducible single TF or combinations of two, three, or four TFs, respectively. Positive genetically modified iPSCs were selected based on antibiotic resistance genes, maintained as monolayer cultures under continuous selection pressure, and propagated in the pluripotent state for 3 days before initiation of differentiation (day -3 to day 0). On day 0, phase I (hemato-endothelial forward programming) was started with a change to differentiation medium and an initial mesodermal priming boost by a high GSK3 inhibitor (CHIR990221) dose. After mesodermal priming, induction of ectopic TF expression was initiated by addition of Dox on day 1. Cells were differentiated toward the hemato-endothelial lineage via TF expression and a mixture of supportive hematopoietic and endothelial cytokines (stem cell factor [SCF], thrombopoietin [TPO], interleukin-3 [IL-3], fibroblast growth factor 2 [FGF2], and vascular endothelial growth factor [VEGF]) (Figure 1A). On day 7 (end of phase I), the effect of the different TF/combinations overexpression was assessed based on their potential to induce hemato-endothelial specification. Expression of endothelial markers CD144 and CD73 was used to demarcate angioblast cells, including HEPs ($CD144^+/CD73^-$), from maturating vascular endothelial cells (VECs) ($CD144^+/CD73^+$) that lack hemogenic potential (Choi et al., 2012) (Figures 1B and 1C). Overexpression of *SCL* or *LMO2* alone generated

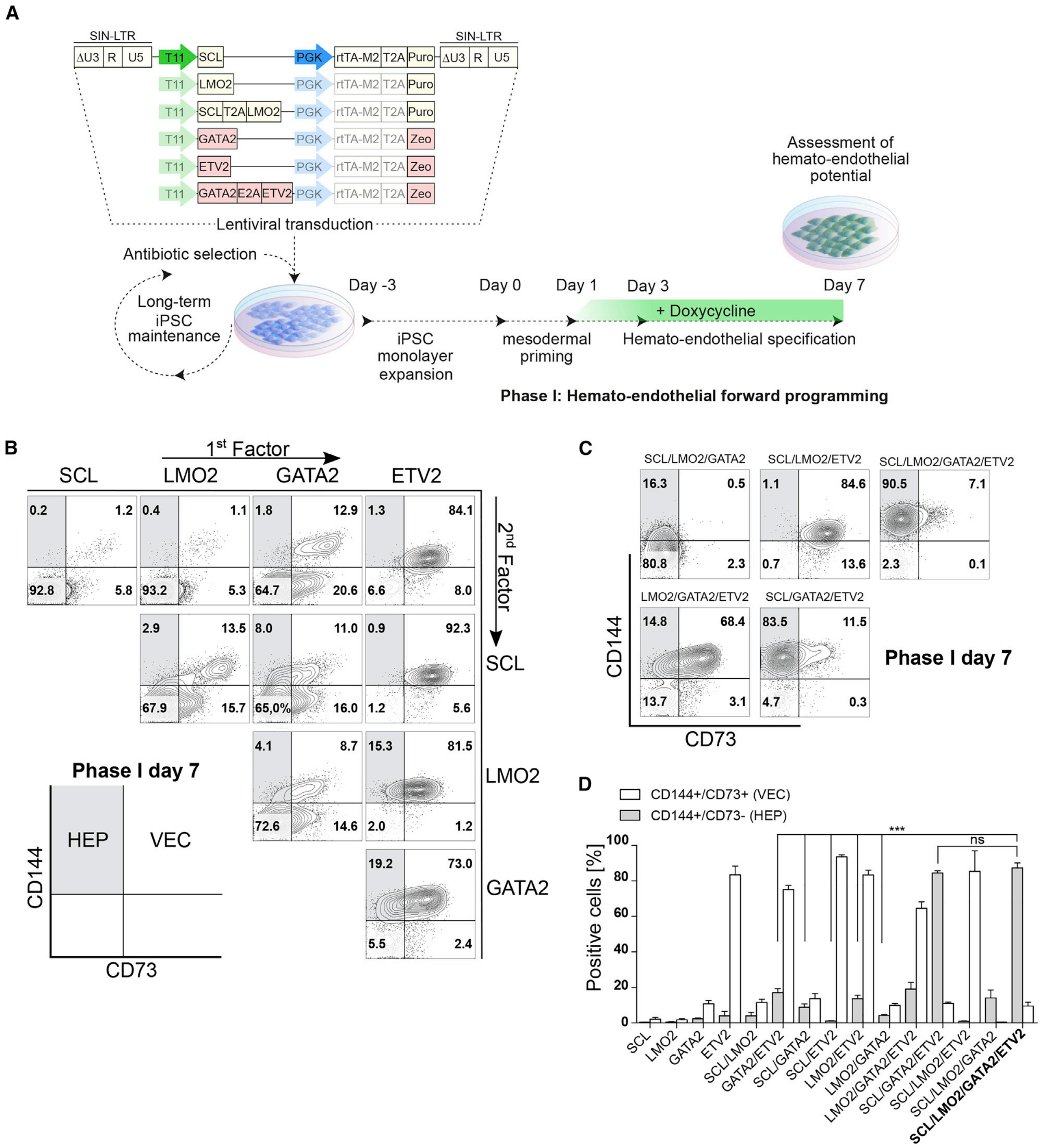

Figure 1. Hemato-endothelial Specification Potential by Induction of Transcriptional Regulators

(A) Schematic vector architecture for ectopic expression of single transcriptional regulators or combinations (*SCL*, *LMO2*, *GATA2*, and *ETV2*) based on a Dox-inducible all-in-one system. These vectors were used to genetically modify and generate stable iPSC lines with subsequent induction of hemato-endothelial specification (phase I).

(B and C) Comparison of individual or combinatorial expression of transcriptional regulators on hemato-endothelial specification potential (phase I day 7). Invididual and two-factor combinations are given in (B) and three-/four-factor combinations in (C). Representative flow cytometric analysis of CD144 and CD73 expression are shown. Samples were gated on fluorescence minus one (FMO) controls.

(D) Statistical analysis of vascular endothelial ($CD144^{+}/CD73^{+}$) or hemato-endothelial ($CD144^{+}/CD73^{-}$) potential of all transcriptional regulators/combinations (n = 3 independent differentiations, error bars represent SD). Significance was calculated by one-way ANOVA with Tukey's post-hoc test (n.s., not significant; $p \geq 0.05$, $^{***}p < 0.001$).

low percentages of cells with VEC or HEP immunophenotypes. *GATA2* overexpression gave rise to an increased population of VECs, but only a low percentage of HEPs. Remarkably, *ETV2* alone was a strong inducer of endothelial specification. However, these endothelial cells displayed almost uniformly a CD144$^+$/CD73$^+$ VEC (84.1%) immunophenotype (Figure 1B). Two-factor combination of *LMO2/SCL* increased the generation of VECs. Furthermore, *LMO2* acted synergistically with *GATA2* and *ETV2* to increase the HEP population from 1.8% (*GATA2* alone) to 4.1% (*LMO2/GATA2*) and 1.3% (*ETV2* alone) to 15.3% (*LMO2/ETV2*) (Figure 1B). The combination of *ETV2/GATA2* increased the percentage of HEPs to 19.2% compared with *ETV2* alone (1.3%) or *GATA2* alone (1.8%). Similarly, *SCL/GATA2* together increased the HEP population from 0.2% (*SCL* alone) or 1.8% (*GATA2* alone) to 8.0%. Given these synergistic effects of the two-factor combinations, we further investigated the additional effect of three- or four-factor combinations (Figure 1C). The addition of *LMO2* (*SCL/LMO2/ETV2* and *LMO2/GATA2/ETV2*) had no substantial change on the immunophenotype compared with the two-factor combinations *SCL/ETV2* or *GATA2/ETV2*, respectively. Interestingly, the combination *SCL/LMO2/GATA2* abrogated formation of VECs and gave rise to a population of HEP immunophenotypic cells with low level CD144 expression. Remarkably, *SCL/GATA2/ETV2* together gave rise to a very high percentage of immunophenotypic HEPs (83.5%). This effect was further increased (90.5%) by addition of *LMO2* (*SCL/LMO2/GATA2/ETV2*) (Figure 1C). These experiments were repeated in three independent differentiations (Figure 1D). Generation of HEPs was significantly increased in the four-factor combination (*SCL*/*LMO2*/*GATA2*/*ETV2*) compared with all two-factor combinations. Although the differences between the best-performing combinations *SCL*/*GATA2*/*ETV2* and *SCL*/*LMO2*/*GATA2*/*ETV2* to generate HEPs were not significant (Figure 1D), we concluded SLGE to be the most potent combination to induce hemato-endothelial specification due to the synergistic effects and the increased generation of HEPs of the two-factor combinations with *LMO2*. Therefore, we used the SLGE-iPSCs as starting material for our forward programming and more detailed analyses.

Generation of Durable Genetically Modified iPSCs with Tightly Regulated Inducible Transgene Expression

To validate the inducibility of the SLGE vector system in detail, we performed qRT-PCR analysis and confirmed a strong increase of exogenous *SCL* (2,393- ± 265-fold), *LMO2* (900- ± 46-fold), *GATA2* (164- ± 3-fold), and *ETV2* (166- ± 12-fold) expression upon Dox treatment. However, low expression levels of all four exogenous TF were also detected in the absence of Dox, likely due to some leakiness of the inducible T11 promoter (Figure S1C). Despite slight expression of TFs in the absence of Dox, SLGE-iPSCs maintained expression of typical pluripotency genes (*OCT4* and *NANOG*) and surface markers (TRA-1-60 and SSEA-4) at levels similar to unmodified H2E6C iPSCs or human embryonic stem cells (Figures S1D and S1E). However, Dox-mediated induction of TF overexpression in SLGE-iPSC maintenance cultures was associated with a dramatic loss of expression of these pluripotency markers (Figures S1D and S1E). Furthermore, non-Dox-induced SLGE-iPSCs retained the full ability to differentiate toward endodermal (lumen-lining epithelium), ectodermal (immature neuroepithelium), and mesodermal (cartilage) cells as visualized in a teratoma formation assay (Figure S1F). Altogether, SLGE-iPSCs could be maintained in a pluripotent state for at least 6 months without spontaneous differentiation (data not shown).

SLGE Robustly Directs Hemato-endothelial Specification and Produces HEP Cells in a Dox-Dependent Manner

To evaluate the effect and potential of SLGE-mediated forward programming, we compared the capacity of SLGE-iPSCs to generate hemato-endothelial cell types with and without ectopic SLGE expression. SLGE-iPSCs were differentiated as described in Figure 1A and analyzed daily by flow cytometry. The differentiation progress of phase I was monitored based on the expression of the common endothelial surface marker CD144 and the VEC marker CD73 under SLGE induction (+Dox, green) or without SLGE induction (–Dox, red) (Figure 2A). CD144 expression was notably increased by day 2 of SLGE induction and the cell population was almost completely CD144$^+$ by day 7. The vast majority of these cells remained negative for CD73, indicating the presence of HEPs. The early endothelial marker CD309 (VEGFR2/KDR) was notably upregulated already on day 2 of SLGE-mediated differentiation (78.4%) compared with day 1 (4.1%) (Figure S2A). Kinetic analysis revealed that CD235a, an erythroid marker that is also expressed on a mesodermal subpopulation fated to primitive hematopoietic progenitors (Sturgeon et al., 2014), was first expressed on day 3 in a subset of cells (40.7%) in the CD144$^+$/CD73$^-$ HEP population (Figure S2B). At this stage, cells were negative for CD43, a marker expressed on emerging hematopoietic cells (Vodyanik et al., 2006). CD43 expression was first detected on day 4 and increased through day 6, especially on CD235a-expressing cells (Figure S2B). The CD235a-expressing subset strongly decreased on day 7 (Figure S2B). Taken together, we obtained a dense, homogeneous monolayer (Figure 2B) with a HEP-like phenotype (~89.0% ± 2.0% CD144$^+$/CD73$^-$) and only a small subpopulation

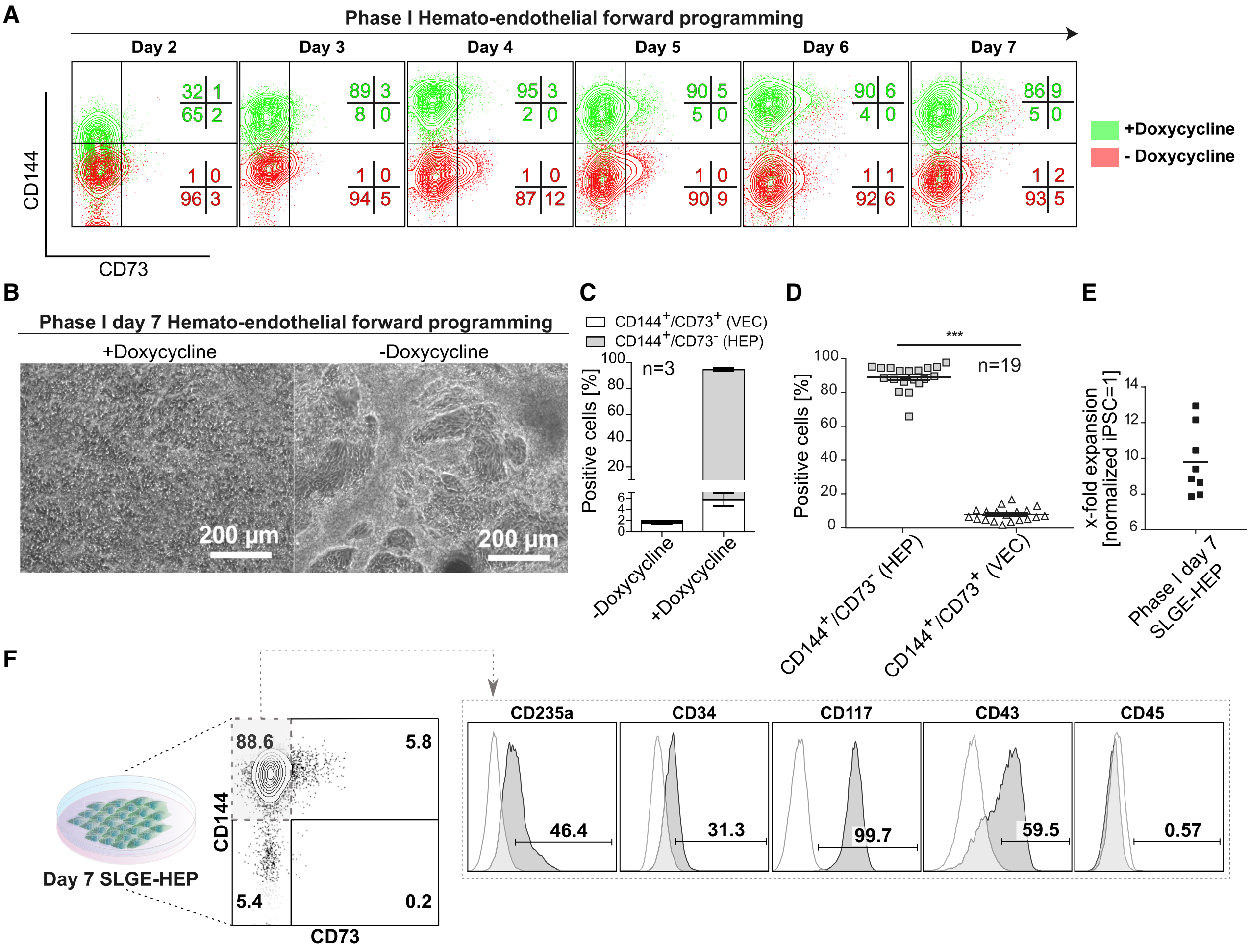

Figure 2. Phase I: Characterization of SLGE-mediated Hemato-endothelial Forward Programming

(A) Kinetic immunophenotype analysis of phase I based on the expression of CD144 and CD73 with and without induction (+/−Dox) of SLGE. Percentages of the individual populations are indicated in the same color as the populations.

(B) Representative microscopic analysis of SLGE-iPSC phase I day7 with and without induction of SLGE overexpression (+/−Dox). Scale bars, 200 μm.

(C) Influence of SLGE induction on HEP ($CD144^{+}/CD73^{-}$) and VEC ($CD144^{+}/CD73^{+}$) production at phase I day7 (+Dox) (n = 3 independent differentiations, error bars represent SD).

(D) Overall yield of Dox-induced SLGE-HEPs ($CD144^{+}/CD73^{-}$) and vascular endothelial cells ($CD144^{+}/CD73^{+}$) (n = 19 individual differentiations). p values were calculated using unpaired t test. Significance is indicated by asterisks: (***$p < 0.001$).

(E) Expansion rate of SLGE-HEPs was calculated with respect to the number of initially seeded iPSCs (n = 8 independent experiments).

(F) Surface marker expression of CD144, CD73, CD235a, CD117, CD43, CD45, and CD34 evaluated by flow cytometry. SLGE-HEPs showed an HEP phenotype of $CD144^{+}/CD73^{-}/CD43^{+/-}/CD235a^{+/-}/CD117^{+}/CD34^{-/+}/CD45^{-}$ (gated on FMO).

with a VEC immunophenotype (~5.0% ± 2.4% $CD144^{+}/CD73^{+}$) on day 7 (Figure 2C). Importantly, the overall potential of endothelial differentiation and HEP generation was highly dependent on the induction of SLGE expression. Without Dox, differentiated cells exhibited a heterogeneous morphology with tubular-like structures (Figure 2B) and no substantial upregulation of CD144 or CD73. Only a small population of these cells exhibited a VEC or HEP immunophenotype on day 7 of differentiation (1.6% ± 0.3% or 0.4% ± 0.1%, respectively) (Figure 2C). Removal of supportive cytokines during phase I increased the VEC population at the expense of HEPs. Mesodermal priming by CHIR, before SLGE induction, was dispensable for efficient HEP generation (Figure S2C). The robustness of the TF-mediated forward programming protocol was shown in 19 independent differentiation experiments

that generated an average level of 89.0% ± 7.5% phenotypic HEPs and 7.7% ± 3.9% VECs (Figure 2D). Phase I day 7 HEPs (SLGE-HEPs) expanded 9.8- ± 2-fold (n = 8) with respect to the initial number of SLGE-iPSCs seeded (Figure 2E). SLGE-HEPs showed an intermediate CD235a expression, moderately expressed CD34 and were positive for CD117 (c-Kit) (Figure 2F), indicating an HE-like phenotype (Ditadi et al., 2016). However, these SLGE-HEPs already expressed the very early hematopoietic marker CD43 but were negative for the pan-hematopoietic marker CD45 (Figure 2F).

SLGE-HEPs Generate HPCs

Although SLGE-HEPs expressed the early hematopoietic marker CD43, they were not fully committed to the hematopoietic fate at this stage as SLGE-HEPs acquired a VEC immunophenotype (CD144$^+$/CD73$^+$/CD34$^+$) within 7 days of cultivation in EGM2 (endothelial growth medium) without Dox-induced TF expression (Figure S3A). These SLGE-VECs were adherent (Figure S3B), able to form tubular-like structures (Figure S3C) and negative for CD45 under endothelial conditions (Figure S3A). More importantly, hematopoietic cells were generated from SLGE-HEPs by cultivation of dissociated SLGE-HEPs in STEMdiff APEL 2 medium supplemented with supportive cytokines (SCF, TPO, FLT3L, IL-3, and FGF2) for hematopoietic development (phase II: generation of HPCs). Noteworthy, phase II was accomplished in the absence of Dox to avoid ectopic SLGE expression and any potential effects on the progression or the phenotype of generated hematopoietic cells. On day 9 of phase II, cells formed compact clusters with adherent cells loosely connected to the surface. The clusters increased in size and generated numerous round-shaped suspension cells within 5 days of phase II (day 11) (Figure 3A). During this progression, HEPs successively lost endothelial characteristics and gained a hematopoietic signature. To more closely investigate this process, we monitored the expression kinetics of endothelial (CD144) and hematopoietic (CD45) markers throughout phase II (days 7–11). Initially, SLGE-HEPs were CD144$^+$ and expressed CD34 at a low level. From day 7 to 8, CD144$^+$ cells showed a notable increase in CD34 expression with subsequent CD144 downregulation, resulting in an almost complete loss of endothelial signature by day 11. In contrast, CD45 expression was strongly upregulated along with CD34 expression by day 8 and peaked on day 10 of phase II. The complete loss of CD144 expression and the gain of CD45 indicated a complete EHT and efficient generation of SLGE-iPSC-derived hematopoietic progenitors (SLGE-HPCs) (Figure 3B). The generation of CD45$^+$/CD34$^+$ SLGE-HPCs was very robust (~60% ± 12%, n = 21) (Figure 3C) concomitant with a proliferative expansion rate of 3.2 ± 0.66-fold (n = 7) respective to initially seeded SLGE-HEPs (Figure 3D), and a total expansion rate of ~30-fold respective to initially seeded SLGE-iPSCs on day −3 of the protocol. Detailed flow cytometric analysis indicated a characteristic HPC phenotype. Noteworthy, these CD45$^+$/CD34$^+$ cells expressed the HSC-like markers CD43, CD90, and CD49f at high levels, CD235a intermediately, and were negative for lineage markers CD38 and CD45RA (Figure 3E). Interestingly, this phenotype CD45$^+$/CD34$^+$/CD38$^-$/CD45RA$^-$/CD90$^+$/CD49f$^+$ of SLGE-HPCs was described to characterize multipotential adult HSPCs (Notta et al., 2015). We validated the potential of SLGE-mediated forward programming with two additional previously described iPSC lines, namely hCD34iPSC16 (Ackermann et al., 2014) and iMSMD-cohet 17 (Neehus et al., 2017), which exhibited similar hemato-endothelial and hematopoietic differentiation potentials (Figure S3D). Importantly, also cultivation of SLGE-iPSCs in fully defined StemMACS iPS-Brew XF medium resulted in efficient production of SLGE-HEPs and SLGE-HPCs (Figure S3E).

SLGE-Directed Hemato-endothelial Forward Programming Is Tightly Regulated and Shows Intermediate Key Stages of Hemato-endothelial Specification

To analyze if distinct intermediate developmental stages and embryonic cell types typical for hematopoiesis can be generated with our forward programming protocol, we dissected the entire differentiation progression into different time points according to expression of indicative target genes via qRT-PCR (Figure 4A). As expected, *OCT4* (black line) expression was highest at the PSC state on day 0 and decreased rapidly until day 3. Expression of the typical mesodermal marker *TBXT* (T; Brachyury; blue, dashed line) peaked on day 1 during mesodermal priming and was quickly downregulated. *KDR* (blue line) expression increased to peak on day 2, was maintained at lower expression levels during phase I, and expression further diminished during phase II. Endothelial gene *CDH5* (CD144; green line) expression was first detected between days 1 and 2 and peaked at the end of phase I on day 7, before rapidly decreasing in phase II. The first hematopoietic marker (*RUNX1*; red line, primer pair specific for all *RUNX1* isoforms) was expressed at lower expression levels already between days 1 and 2 and peaked on day 7 with a second peak in phase II on day 10. Expression of *RUNX1* isoform c (*RUNX1c*, dashed red line), a marker indicating HEP and HPC specification, was strongly upregulated on day 7 of phase I and peaked on day 10 during the EHT process. Thus, expression of major hemato-endothelial markers increased directly after induction of SLGE overexpression, but especially *RUNX1c* expression fully peaked during EHT and hematopoietic specification in the absence

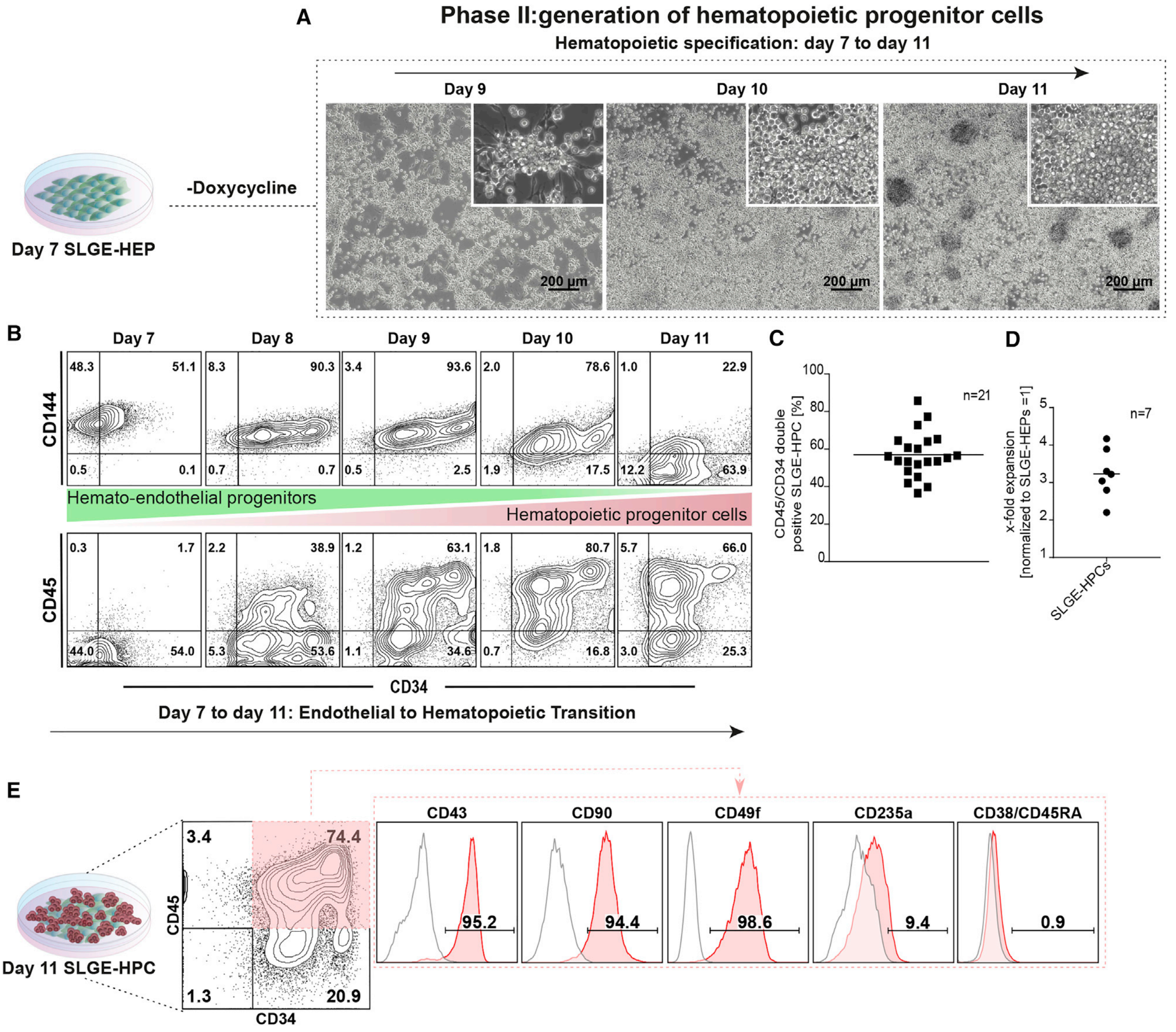

Figure 3. Characterization of SLGE-HPCs

(A) Schematic representation of phase II, generation of SLGE-HPCs. SLGE-HEPs were seeded into medium supplemented with cytokines supportive for hematopoietic specification, without Dox. Morphologic changes are shown by representative microscopy images on days 9–11 of phase II. Scale bars, 200 μM.

(B) Kinetic flow cytometric analysis of endothelial-to-hematopoietic transition phase II (days 7 to 11) for endothelial marker CD144 as well as hematopoietic markers CD45 and CD34.

(C) Overall yield of CD45$^+$/CD34$^+$ SLGE-HPCs (n = 21 independent differentiation experiments).

(D) Expansion rate of SLGE-HPCs as calculated with respect to the number of initially seeded SLGE-HEPs (n = 7 independent differentiation experiments).

(E) Hematopoietic surface marker expression evaluated by flow cytometric analysis (gates were set based upon FMO controls). Day 11 SLGE-HPCs had a CD45$^+$/CD34$^+$/CD235a^{low}/CD43$^+$/CD90$^+$/CD49f$^+$/CD38$^-$/CD45RA$^-$ phenotype.

of Dox-induced TF expression. To further strengthen the observation that our forward programming protocol mimics *in vivo* ontogeny, we performed RNA sequencing analysis during the first differentiation days (Figure 4B). After mesodermal priming (phase I day 1), genes characteristic of early mesodermal stages (*EOMES*, *MESP1*, *MIXL1*, and *T*) were upregulated and subsequently downregulated 1 day later. On day 2 of phase I, 1 day after SLGE induction, upregulation of genes associated with early endothelial/angiohematopoietic specification was detected based on

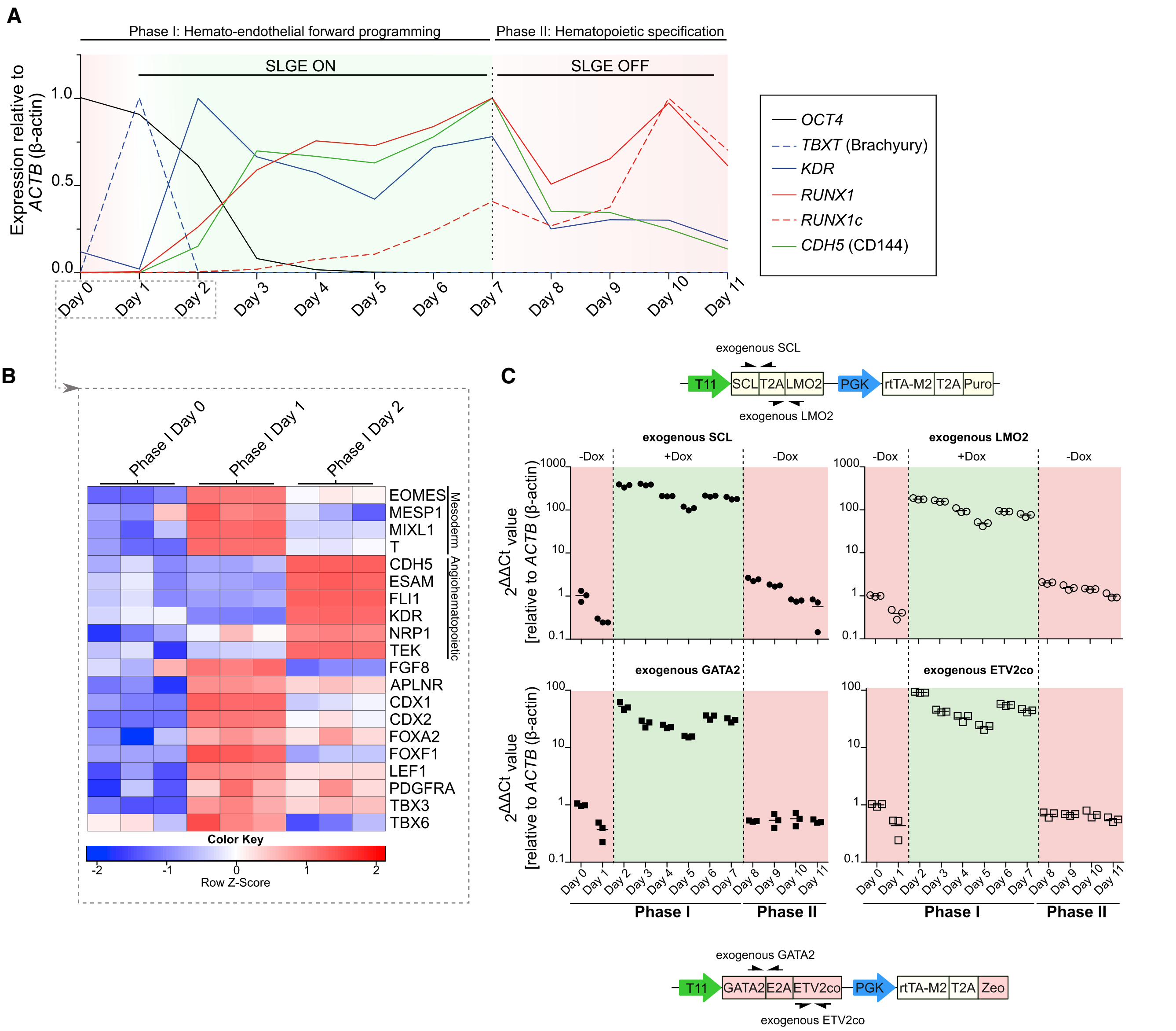

Figure 4. Characterization of Intermediate Developmental Stages and Ectopic SLGE Expression during Hemato-endothelial Forward Programming

(A) qRT-PCR for endogenous mRNA expression levels of target genes indicative for pluripotency (*OCT4*), mesodermal (*TBXT* and *KDR*), endothelial (*CDH5* [CD144]) and hematopoietic (*RUNX1* and *RUNX1c*) stages during the differentiation process. The point of maximum gene expression was set to 1 for each target. Human *ACTB* (β-actin) was used as a housekeeping control and for normalization of target gene expression.

(B) Row-scaled heatmap of characteristic mesodermal- or endothelial-associated genes during early stages of phase I (days 0–2) based on RNA sequencing data. Gene ontology based on the Database for Annotation, Visualization and Integrated Discovery (DAVID) or literature (Choi et al., 2012).

(C) qRT-PCR for Dox-based regulation of exogenous *SCL*, *LMO2*, *GATA2*, and *ETV2* expression during the differentiation process. Expression levels were normalized to basal expression of non-induced day 0 SLGE-iPSCs and relative to *ACTB* (β-actin) housekeeping gene expression in triplicate samples.

RNA sequencing data (e.g., *CDH5*, *ESAM*, *FLI1*, *KDR*, *NRP1*, and *TEK*) (Figure 4B). qRT-PCR confirmed that expression levels of exogenous SLGE were tightly regulated during differentiation by addition or withdrawal of Dox (Figure 4C). Importantly, ectopic SLGE expression levels were constantly high-throughput phase I in the presence of

Dox and abruptly decreased back to basal expression levels within 1 day after Dox withdrawal on day 8 (phase II) at the beginning of EHT and the production of HPCs.

SLGE-HPCs Generate Erythroid, Megakaryocytic, Myeloid, and NK Cells

To demonstrate the hematopoietic potential of SLGE-HPCs, cells were terminally differentiated toward different hematopoietic lineages. SLGE-HPCs produced $CD45^{-}CD41^{+}CD42a^{+}CD61^{+}$ megakaryocytes (Figure 5A) with large and multi-lobulated nuclei upon stimulation with TPO (Figure 5B, red arrows). Differentiation with EPO (erythropoietin) gave rise to $CD45^{-}CD235a^{+}$ erythroid cells (Figure 5A) with normoblast-like cell morphology (Figure 5C). These erythroid cells expressed different globin types (Figure S4A), with embryonic isoforms ζ- and ε-globin, and fetal isoforms γ- and α-globin, as the most prevalent. Adult-type β-globin was only expressed at low levels. Expression of all globin isoforms, including adult globin, was validated by gel electrophoresis of the qRT-PCR products (Figure S4B). In addition, SLGE-HPCs also exhibited myeloid potential. Stimulation with macrophage colony stimulating factor promoted a population of $CD45^{+}CD11b^{+}$ myeloid cells (Figure 5A). This population was divisible into two distinct populations with either classical M2 immunoregulatory macrophage phenotype ($CD14^{+}/CD163^{+}$; marked in green) or M1-like polarized macrophage phenotype ($CD14^{-}/CD163^{-}/CD209^{-}$ and $CD86^{+}$, marked in red). Macrophages showed characteristic cytoplasmatic inclusion and a single, round-shaped nucleus (Figure 5D). $CD45^{+}/CD14^{-/low}$ cells emerged upon differentiation with granulocyte colony stimulating factor (Figure 5A). These cells expressed the granulocyte marker CD66b (~6%) and stained positive for CD15 and CD16 at a low level. Despite the low amount of $CD66b^{+}$ cells, cytospin images revealed that a large number of cells exhibited classical granulocytic morphology at different developmental stages (e.g., myelocytes and mature segmented neutrophils; Figure 5E). For lymphoid differentiation, SLGE-HPCs were co-cultured with OP9-Dll1 stromal cells. This led to generation of a clear population of $CD45^{+}CD56^{+}CD16^{-}$ NK cells upon cultivation in medium containing FLT3L/IL-7/IL-15 (Figure 5A). However, we did not detect any T cells under OP9-Dll1 co-culture differentiation conditions (data not shown). Furthermore, progenitor character of phase II day 11 SLGE-HPCs was confirmed in colony-forming cell assays in which cells proliferated and formed all lineage-specific and mixed colonies (CFU-M, CFU-GEMM, CFU-E, CFU-GM, and CFU-G) (Figure 5F). However, colony-forming potential was biased as most colonies (produced from 1,000 initially seeded cells) were CFU-M (56 ± 9 colonies) and CFU-E (47 ± 19 colonies), whereas CFU-GEMM (10 ± 3 colonies), CFU-GM (7 ± 8 colonies), and CFU-G (8 ± 2 colonies) were less prominent. To investigate SLGE-HPC engraftment function, 2×10^{6} SLGE-HPCs were intravenously transplanted into irradiated NSGS mice, but SLGE-HPCs showed no long-term engraftment or reconstitution potential in contrast to cord blood (CB)-HSPCs (Figure S4C). Presence of human SLGE-HPC-related $CD45^{+}$ cells was detected in peripheral blood up to 2 weeks after transplantation in two of three mice, but not at weeks 4, 6, or 8. In contrast to human CB-transplanted mice, SLGE-iPSC-derived hematopoietic cells were not found in bone marrow or spleen 8 weeks post-transplantation (Figure S4D).

RNA Sequencing Comparison between CB-HSCs and SLGE-HPCs Reveals Similarities but Also Major Differences

The HSC-like surface immunophenotype ($CD45^{+}/CD34^{+}/CD90^{+}/CD49f^{+}/CD38^{-}/CD45RA^{-}$), proliferation potential and ability to generate different blood-lineages (including NK cells) suggested a close developmental correlation between SLGE-HPCs and early HSPCs, whereas the incapacity to differentiate into T cells and to engraft into immunodeficient mice also revealed functional and qualitative differences. To unravel the similarities and underlying differences between SLGE-HPCs and CB-HSPCs, we performed whole transcriptome analysis on distinct populations based on a previously described sorting strategy (Notta et al., 2015). SLGE-HPCs and human umbilical CB-HSCs were sorted for $CD45^{+}/CD34^{+}/CD38^{-}/CD45RA^{-}/CD90^{high}/CD49f^{high}$-expressing cells (Figure S5) and compared with SLGE-iPSCs ($TRA\text{-}1\text{-}60^{+}/SSEA4^{+}$) and SLGE-HEPs ($CD144^{+}/CD73^{-}$). Principal component analysis showed clear separation of SLGE-iPSCs (blue dots), SLGE-HEPs (yellow), SLGE-HPCs (orange dots) and CB-HSCs (red dots) (Figure 6A). Importantly, although SLGE-HPCs formed a separate cluster, they were positioned closest to CB-HSCs, reflecting a gradual differentiation toward HPCs during the protocol. Hierarchical clustering confirmed the overall similarity of SLGE-HPCs and CB-HSC transcriptomes, in contrast to SLGE-iPSCs and SLGE-HEPs (Figure 6B). Moreover, genes involved in hematopoietic development and HSC function were similarly expressed in SLGE-HPCs and CB-HSCs (Figure S6A). Of note, many genes and TFs (Figure S6A, black arrows) previously used in transdifferentiation settings (Batta et al., 2014; Riddell et al., 2014; Sandler et al., 2014; Sugimura et al., 2017; Vo et al., 2018) and forward programming approaches (Elcheva et al., 2014) were expressed at similar levels in SLGE-HPCs and CB-HSCs. Although exogenous SLGE expression was decreased to basal expression levels (Figure 4C), SLGE-HPCs expressed endogenous *SCL*, *LMO2*, and *GATA2* at physiological levels, similar to CB-HSCs. *ETV2* expression was slightly increased compared with CB-HSCs. However, expression of a number

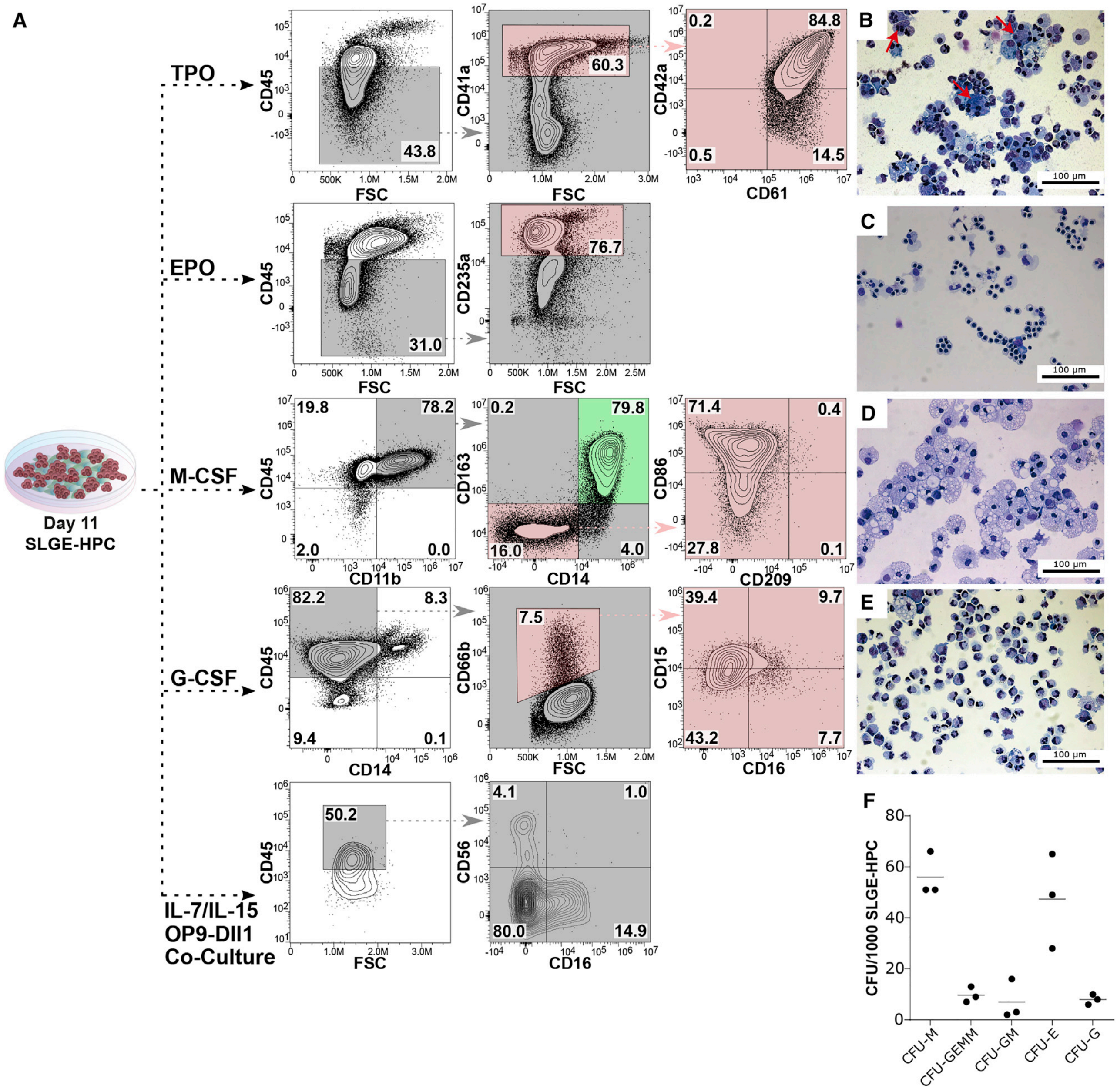

Figure 5. Terminal Differentiation of SLGE-HPCs into Mature Blood Cell Lineages

(A) For terminal differentiation, SLGE-HPCs were stimulated with TPO for 14 days (megakaryocytic specification), with EPO for 7 days (erythroid specification), with macrophage colony-stimulating factor (M-CSF) for 7 days (monocytic/macrophage specification), with granulocyte colony-stimulating factor (G-CSF) for 7 days (granulocyte specification), or with FLT3L/IL-7/IL-15 for 14 days in co-cultivation on OP9-Dll1 cells (NK cell specification). Flow cytometry analysis of terminal differentiation for typical surface marker expression for megakaryocytes ($CD45^-$, $CD41a^+$, $CD42a^+$, and $CD61^+$), erythroid cells ($CD45^-$ and $CD235a^+$), macrophages ($CD45^+$, $CD11b^+$, $CD14^+$ or $CD14^-$, $CD163^+$ or $CD163^-$, $CD86^+$ or $CD86^-$, and $CD209^-$), granulocytes ($CD45^+$, $CD14^{-/low}$, $CD66b^+$, $CD15^+$, and $CD16^+$) (gates were set according to FMO controls) and NK cells ($CD45^+$, $CD56^+$, and $CD16^-$) (gates were set according to unstained control).

(B–E) (B) Cytospins of day 14 differentiated megakaryocytes (red arrows), (C) differentiated erythroid cells, (D) terminally differentiated macrophages, or (E) differentiated granulocytes.

(F) Proliferation and multi-lineage differentiation ability of phase II day 11 SLGE-HPCs confirmed by colony-forming assays. Colonies were counted and characterized 14 days after initiation (n = 3 independent experiments).

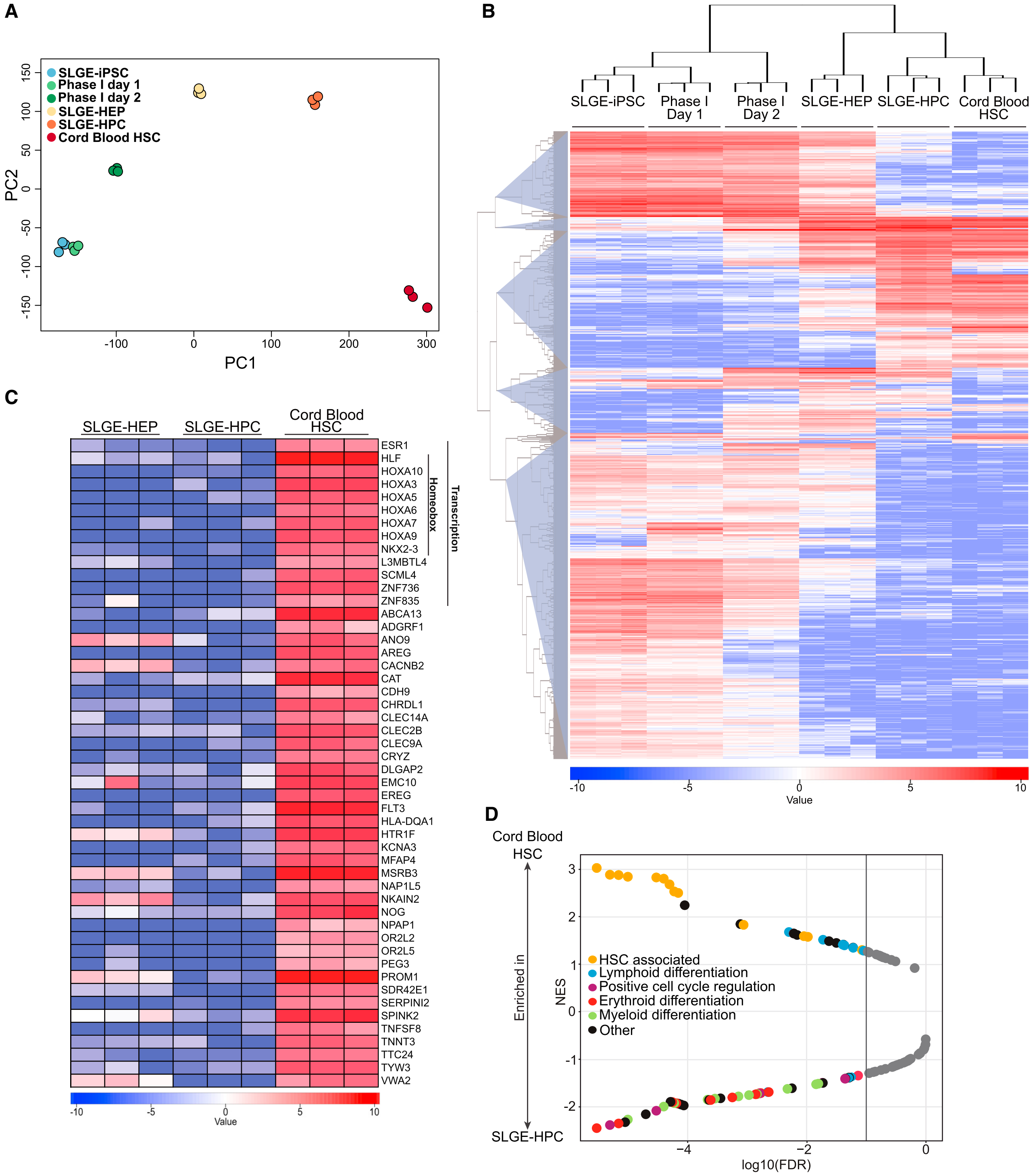

Figure 6. RNA sequencing Comparison between SLGE-iPSC-Derived HEPs/HPCs, and CB-HSCs

(A) Principal-component analysis (PCA) for all 22,214 genes over dimensions 1 and 2 with samples colored by groups.

(B) Unsupervised heatmap of the top 5% of genes (n = 1,111) with the highest variance. Samples are ordered by hierarchical clustering, with the dendrogram above indicating the sample clustering. Similarity between each sample is equal to the positioning within the hierarchical tree. Genes are subdivided into five main clusters by hierarchical clustering based on similar gene expression.

(legend continued on next page)

of HSC-associated genes was lacking in SLGE-HPCs. Especially transcriptional regulators with a crucial role in hematopoietic development or HSC homeostasis, such as *HLF* (Gazit et al., 2013) or homeobox TF of the *HOXA* (Dou et al., 2016; Lawrence et al., 2005) gene cluster or the HSC surface marker *PROM1*/CD133 were expressed at much lower levels or not expressed in SLGE-HPCs (Figure 6C). In addition, failure of SLGE-HPCs to silence genes that are repressed or not expressed in CB-HSCs could be crucial for HSC characteristics and functions (Figure S6B). Other genes involved in self-renewal of PSCs (*NANOG*, *POU5F1*, and *SOX2*) were clearly downregulated in SLGE-HPCs (Figure S6C). Direct, pairwise comparison of gene set enrichment analysis (Schwarzer et al., 2017) (Table S3) indicated that the genes that were downregulated in SLGE-HPCs compared with CB-HSPCs were associated with HSC phenotype (Figure 6D) (yellow dots). Genes enriched in SLGE-HPCs are involved in myeloid (green dots) or erythroid (red dots) differentiation and cell-cycle regulation (purple dots). Particular genes involved in lymphoid differentiation (blue dots) were slightly more enriched in CB-HSCs. The transcriptome analysis supports the *in vitro* lineage potential of SLGE-HPCs and suggests the need for additional up- and/or downregulation of certain TFs or governing regulatory pathways.

DISCUSSION

iPSC technology and the ability to generate pluripotent cells from patients with diverse hematological diseases offers the opportunity to model various diseases and gain precise information about molecular pathways, transcriptional networks, and ontogenetic processes. However, application of iPSC-derived hematopoietic cells is often restricted by demanding protocols to generate large numbers of HSPCs and terminally differentiated hematopoietic cells in a robust manner. In addition, the output of most protocols is quite variable in terms of quantity, quality, and differentiation status. These limitations restrict the use of iPSC-derived HSPCs for large *in vitro* and *in vivo* experiments, drug screens, and especially possible future clinical applications. The challenge to generate HSPCs from iPSCs is already apparent at the beginning of the differentiation process by the difficulty to generate sufficient amounts of HEPs, which often need to be purified from heterogeneous cell populations before hematopoietic specification.

Here, we established a defined, robust, and efficient protocol based on TF-mediated hemato-endothelial forward programming of hiPSCs by temporally controlled overexpression of *SCL*, *LMO2*, *GATA2*, and *ETV2*. Each of these TFs was identified as a critical regulator for mesodermal, endothelial, and/or hematopoietic specification and their functions were evaluated in animal models (Kataoka et al., 2011; Ling et al., 2004; Org et al., 2015; Yamada et al., 2000). Interestingly, SCL- and GATA-binding proteins were identified to act synergistically with LMO2 to form a transcriptional *trans*-activating complex that regulates primitive hematopoietic ontogeny in vertebrates (Mead et al., 2001). Similarly, *GATA2* and *ETV2* are co-expressed and interactively regulate early stages of hematopoietic development (Liu et al., 2015; Shi et al., 2014). In our gain-of-function screening, we unraveled the functions of these key TFs with regard to early hemato-endothelial specification. We identified *ETV2* as a remarkably potent inducer of endothelial differentiation that produces cells with a predominantly VEC phenotype ($CD144^{+}/CD73^{+}$) when expressed alone. However, combination of *ETV2* with *LMO2* or *GATA2* induced the production of HEPs ($CD144^{+}/CD73^{-}$). This effect was even more pronounced by combined overexpression of *SCL/GATA2/ETV2* or *SCL/LMO2/GATA2/ETV2*. Although this study focused on the four-factor combination *SCL*/*LMO2*/*GATA2*/*ETV2*, the differences in induction of HEPs between this combination and the three-factor combination *SCL*/*GATA2*/*ETV2* were not significant (Figure 1D). Thus, the exogenous expression of *LMO2* might not be essential for HEP specification. Endogenous LMO2 might be sufficient to facilitate formation of complexes and protein-protein interactions of the overexpressed TFs (Stanulović et al., 2017). As the role of *LMO2* during the differentiation process is not entirely clear, this should be explored in future studies. However, this screening experiment identified the combination *SCL/LMO2/GATA2/ETV2* to be highly potent for specific and robust angio-hematopoietic specification of iPSCs. The combinatorial effect of SLGE-mediated forward programming and cytokine-based directed differentiation efficiently generated SLGE-HEPs. The cytokines, used during phase I, supported the formation of SLGE-HEPs while preventing VEC specification (Figure S2C). Mesodermal priming by CHIR seemed not to be the most essential component for efficient SLGE-HEP generation (Figure S2C), which might be compensated by SLGE overexpression.

(C) Heatmap of the top 50 protein-coding genes not induced in SLGE-HPCs (mean $\log_2 FC = -10.1$, $p_{adj} < 0.004$). Gene ontology (Transcription and Homeobox) based on *DAVID* otherwise in alphabetical order.

(D) Gene set enrichment analysis of coordinated gene expression changes in 117 hematopoiesis-associated gene sets (Schwarzer et al., 2017) (Table S3) in SLGE-HPCs and CB-HSCs. Normalized enrichment scores (NES) are plotted against the false discovery rate (FDR). Significant enrichment (FDR < 0.1) is indicated by the vertical line.

We hypothesize that the four TFs in our system act synergistically in one regulatory complex and contribute to one gene regulatory network. The combination of murine *Erg* (such as the *ETV2* member of the ETS TF family), *Gata2*, *Lmo2*, *Runx1c*, and *Scl* was previously used to reprogram murine fibroblasts into HPCs with short-term engraftment and multi-lineage potential (Batta et al., 2014). A gain-of-function screen to evaluate TFs identified *GATA2* and *ETV2* as crucial TFs for hematopoietic induction. Constitutive overexpression of *GATA2* and *ETV2* directly induced endothelium with hematopoietic, pan-myeloid potential, but without engraftment (Elcheva et al., 2014). In contrast to these approaches, our SLGE-based forward programming protocol is fully inducible and requires overexpression of TF only for a short period to rapidly generate large numbers of highly pure SLGE-HEPs without effecting the HPC production. Although SLGE-HEPs expressed CD43, an early marker of hematopoietic commitment (Vodyanik et al., 2006), they are not fully committed to hematopoietic lineages. SLGE-HEPs were still capable to mature into adherent cells with VEC phenotype ($CD144^+/CD73^+/CD34^+/CD45^-$) under endothelial conditions. Importantly, SLGE-HEPs undergo EHT and produce hematopoietic cells with an HSC-like phenotype, which proceeds in the absence and without the influence of ectopic SLGE expression. The expression of early mesodermal markers (e.g., *EOMES*, *MESP1*, *MIXL1*, and *T*), differentiation into KDR^+ cells, subsequent endothelial specification (CD144 expression), EHT, and finally generation of $CD45^+/CD34^+$ SLGE-HPCs, suggests that the SLGE-based forward programming recapitulates early stages of embryonic hematopoiesis and mimics important developmental stages *in vitro*. Thus, this protocol—and the large quantity of HEPs and HPCs generated—offers a robust basis to decipher early developmental processes and may help to identify additional gene regulatory networks that directly alter mesodermal specification, HE development, the EHT process, and generation of fully functional HSPCs. Despite all attempts, *de novo* generation of *bona fide* human HSPCs *in vitro* is still challenging and currently relies on genetic modifications of HE cells with up to seven exogenous TFs (Sugimura et al., 2017). However, this procedure requires the generation of large amounts of iPSC-derived HE for genetic modification and modified HE cells gain their HSPC-like potential *in vivo* only after direct injection into the bone marrow of recipient mice. Our SLGE-iPSC-derived HPCs share some HSPC characteristics, but generally exhibit a rather predominant erythro-megakaryocytic and myeloid potential, and a restricted lymphoid lineage potential with a limited proliferative potential at the HPC level and only short-term persistence after transplantation into recipient mice. In addition, the predominant expression of embryonic and fetal hemoglobin and a minor expression of adult globin indicate a primitive hematopoietic phenotype of the majority of SLGE-HPCs. This might be attributed to a rather primitive hematopoietic capacity of generated HEPs with the current protocol, but may also indicate functional heterogeneity of SLGE-HEPs regarding definitive hematopoietic potential. HE heterogeneity can be attributed to early mesodermal specification and depends, among other factors, on Wnt-β-catenin and activin-nodal signaling (Sturgeon et al., 2014). Activation of an arterial program through overexpression of the TF *ETS1* in HE promoted a definitive, arterial hematopoietic program with T- and B-lymphoid potential. *ETS1* and *ETV2* belong to the same ETS TF family and have a highly conserved DNA binding domain (ETS domain). ETS TFs play an important role in regulation of endothelial genes (Lammerts van Bueren and Black, 2012) and later in HSC specification and maintenance (Loughran et al., 2008). Regulation of different endothelial genes and diverse primitive and definitive HE genes might be orchestrated by different ETS transcriptional regulators or a combination of factors, respectively. Ectopic expression of *ETS1* in addition to SLGE or the exchange of *ETV2* for *ETS1* may induce a more arterial/definitive HE type and induce a rather definitive hematopoietic progenitor phenotype. Despite the assumption of the primitive polarization of our SLGE-HEPs, the newly produced SLGE-HPCs exhibited an HSC surface marker signature found in CB-HSCs and adult bone marrow HSCs ($CD45^+/CD34^+/CD38^-/CD45RA^-/CD90^+/CD49f^+$). Interestingly, our RNA sequencing data demonstrated that SLGE-iPSC-derived HEP/HPCs and CB-HSCs share a high degree of overlap in common hematopoietic and especially HSC gene expression. Of note, many TFs used in forced differentiation (Batta et al., 2014; Doulatov et al., 2013; Riddell et al., 2014; Sugimura et al., 2017) and transdifferentiation protocols (Sandler et al., 2014) are expressed in SLGE-iPSC-derived HEP/HPCs. However, major differences were also noted and gene set enrichment analysis revealed enrichment of myeloid and erythroid genes in SLGE-iPSC-derived HPCs. The lack of crucial hematopoietic TFs may explain the absence of long-term engraftment of our SLGE-HPCs. In particular, SLGE-iPSC-derived HPCs exhibited lower expression of the *HOXA* gene cluster, which was described to facilitate HSC function and self-renewal *in vivo* (Lawrence et al., 2005). Defective medial *HOXA* gene activation was previously observed in ESC-derived HSPCs and was described to be a crucial developmental barrier to establish ESC-derived HSCs with self-renewal potential (Dou et al., 2016). The *HOXA* family was also implicated in HSPC specification in other examples of forced differentiation and transdifferentiation. The combinatorial expression of *HOXA9* and other transcriptional regulators converted hPSC-derived myeloid-restricted precursor cells into multi-lineage HSPCs

(Doulatov et al., 2013) or conferred multi-lineage engraftment potential to undirected differentiated HE as one of the abovementioned seven TFs (*ERG*, *HOXA5*, *HOXA9*, *HOXA10*, *LCOR*, *RUNX1* and *SPI1*) (Sugimura et al., 2017). Thus, activation of the *HOXA* gene cluster and/or ectopic overexpression of *HOXA* genes may govern hematopoietic fate determination in the HEP state and confer self-renewal potential to SLGE-HPCs. Despite some differences between SLGE-HPCs and *bona fide* HSCs, our described platform provides a powerful tool to generate sufficient numbers of HEPs for large screening experiments. This will help to identify combinations of synergistically acting TF and signaling pathways that govern early hematopoietic development (e.g., mesoderm specification and arterial endothelial development). Furthermore, our current protocol overcomes the limitation of producing pure fractions and high numbers of HEPs and HPCs as a single 12-well of initially seeded SLGE-iPSCs routinely generated ~4.5 × 10^6 SLGE-HEPs and ~7 × 10^6 ($CD45^+$/$CD34^+$) SLGE-HPCs in only 11 days. Therefore, we propose that this system can also be utilized to establish new hematological disease models and perform drug screening or gene therapy approaches to discover and establish new suitable treatment options.

EXPERIMENTAL PROCEDURES

Design of the Inducible Vector System

Coding sequences for human TF *SCL* (GenBank: M61108.1), *LMO2* (GenBank: BC035607.1), *GATA2* (GenBank: M68891.1), and *ETV2* (*ETV2* codon-optimized) (GenBank: NM_014209.3) were cloned into previously described tet-inducible, third-generation SIN-lentiviral vectors (Heinz et al., 2011), which co-expressed the transactivator rtTA.M2 (M2) in an all-in-one design. TF cassettes were arranged in a monocistronic or bicistronic configuration to generate the vectors as shown in Figure 1A. Expression of TFs was driven and regulated by a tet-inducible (T11) promoter and bicistronic expression was achieved using self-cleaving 2A-peptides (T2A or E2A). To enable positive selection of transduced cells, P2A-puromycin (Puro) or P2A-zeocin (Zeo) selection marker cassettes were inserted in frame, downstream of the M2 transactivator and constitutively expressed by the human PGK promoter (cloning details are available upon request).

Directed Hemato-endothelial Differentiation

Genetically modified human SLGE-iPSCs were seeded at a density of 1 × 10^6 cells per 9-cm^2 culture dish in conditioned iPSC medium (or StemMACS iPS-Brew XF stem cell medium) containing 10 μM Y-27632 on day −3. After 3 days, medium was changed to RPMI medium (PAN Biotech), supplemented with 100 U mL^{-1} penicillin, 100 μg mL^{-1} streptomycin (PAN Biotech), 0.5% non-essential amino acids (Gibco) and 2% B27 supplement without insulin (Gibco) ($RB27^-$). This point of differentiation was considered as day 0 and as initiation of phase I (Figure 1A). Cells were sequentially cultivated with small molecules and recombinant cytokines in the following order: day 0: $RB27^-$ medium containing 8 μM CHIR99021 (Axon Medchem, Groningen, the Netherlands); day 1: $RB27^-$ supplemented with 1 μg mL^{-1} Dox (Sigma-Aldrich) to induce SLGE; days 2–7: StemPro34 SFM (Thermo Fisher Scientific), 100 U mL^{-1} penicillin, 100 μg mL^{-1} streptomycin, 1 μg mL^{-1} Dox, 6 μM SB431542 (Leibniz University Hannover), 100 ng mL^{-1} SCF, 20 ng mL^{-1} FGF2, 50 ng mL^{-1} TPO, 15 ng mL^{-1} VEGF, 25 ng mL^{-1} IL-3 (all Peprotech, Hamburg, Germany). On day 7 of differentiation and the end of phase I, SLGE-HEPs were dissociated with Accutase and seeded for phase II (Figure 3A). Hematopoietic specification was accomplished on 0.1% gelatin (Sigma-Aldrich)-coated cell culture dishes, at a density of 2.5 × 10^6 cells per 9-cm^2 culture dish in STEMdiff APEL 2 (STEMCELL Technologies, Cologne, Germany) supplemented with 100 U mL^{-1} penicillin, 100 μg mL^{-1} streptomycin, 100 ng mL^{-1} SCF, 20 ng mL^{-1} FGF2, 50 ng mL^{-1} TPO, 100 ng mL^{-1} FLT3L, 25 ng mL^{-1} IL-3 (all Peprotech) without Dox for hematopoietic differentiation (start of phase II). Cells were cultivated in this medium for 5 days (day 11 of differentiation) to generate SLGE-HPCs. Additional experimental procedures are provided in Supplemental Experimental Procedures.

ACCESSION NUMBERS

The accession number for the RNA sequencing data reported in this paper is GEO database: GSE140221.

SUPPLEMENTAL INFORMATION

Supplemental Information can be found online at https://doi.org/10.1016/j.stemcr.2019.11.005.

AUTHOR CONTRIBUTIONS

L.L. designed and performed experiments, analyzed data, and wrote the manuscript. D.H. designed and analyzed experiments, discussed the results, and edited the manuscript. T.-C.H., F.P., and D.L. designed, performed, and analyzed experiments. A.Schwarzer designed and analyzed experiments. M.M. analyzed experiments, discussed results, and edited the manuscript. A.Schambach supervised the study, contributed to lentiviral vectors, provided conceptual advice, discussed the results, and edited the manuscript.

ACKNOWLEDGMENTS

We thank M. Ballmaier (Cell Sorting Facility, Hannover Medical School) for scientific support. Oliver Dittrich-Breiholz and Torsten Glomb (Research Core Unit Transcriptomics, Hannover Medical School) for scientific support, design, and help with the transcriptome analysis. We thank Georges Lacaud for helpful suggestions on forward programming. We thank Kerstin Kaufmann (John Dick's Lab, UHN, Toronto, Canada). This work was supported by grants from DFG, Germany (REBIRTH Cluster of Excellence EXC62/2 and SFB738). In addition, this project has received funding from the European Research Council (ERC) under the European Union's Horizon 2020 research and innovation programme (Grant agreement No. 819531).

Received: January 25, 2019
Revised: November 12, 2019
Accepted: November 15, 2019
Published: December 12, 2019

REFERENCES

Ackermann, M., Lachmann, N., Hartung, S., Eggenschwiler, R., Pfaff, N., Happle, C., Mucci, A., Göhring, G., Niemann, H., Hansen, G., et al. (2014). Promoter and lineage independent anti-silencing activity of the A2 ubiquitous chromatin opening element for optimized human pluripotent stem cell-based gene therapy. Biomaterials *35*, 1531–1542.

Batta, K., Florkowska, M., Kouskoff, V., and Lacaud, G. (2014). Direct reprogramming of murine fibroblasts to hematopoietic progenitor cells. Cell Rep. *9*, 1871–1885.

Bertrand, J.Y., Giroux, S., Golub, R., Klaine, M., Jalil, A., Boucontet, L., Godin, I., and Cumano, A. (2005). Characterization of purified intraembryonic hematopoietic stem cells as a tool to define their site of origin. Proc. Natl. Acad. Sci. U S A *102*, 134–139.

Blaser, B.W., and Zon, L.I. (2018). Making HSCs in vitro: don't forget the hemogenic endothelium. Blood *132*, 1372–1378.

de Bruijn, M.F.T.R., Ma, X., Robin, C., Ottersbach, K., Sanchez, M.-J.J., and Dzierzak, E. (2002). Hematopoietic stem cells localize to the endothelial cell layer in the midgestation mouse aorta. Immunity *16*, 673–683.

Choi, K.D., Vodyanik, M.A., Togarrati, P.P., Suknuntha, K., Kumar, A., Samarjeet, F., Probasco, M.D., Tian, S., Stewart, R., Thomson, J.A., et al. (2012). Identification of the hemogenic endothelial progenitor and its direct precursor in human pluripotent stem cell differentiation cultures. Cell Rep. *2*, 553–567.

D'Souza, S.S., Kumar, A., and Slukvin, I.I. (2018). Functional heterogeneity of endothelial cells derived from human pluripotent stem cells. Stem Cells Dev. *27*, 524–533.

Ditadi, A., Sturgeon, C.M., and Keller, G. (2016). A view of human haematopoietic development from the Petri dish. Nat. Rev. Mol. Cell Biol. *18*, 56–67.

Dou, D.R., Calvanese, V., Sierra, M.I., Nguyen, A.T., Minasian, A., Saarikoski, P., Sasidharan, R., Ramirez, C.M., Zack, J.A., Crooks, G.M., et al. (2016). Medial HOXA genes demarcate haematopoietic stem cell fate during human development. Nat. Cell Biol. *18*, 595–606.

Doulatov, S., Vo, L.T.T., Chou, S.S.S., Kim, P.G.G., Arora, N., Li, H., Hadland, B.K.K., Bernstein, I.D.D., Collins, J.J.J., Zon, L.I.I., et al. (2013). Induction of multipotential hematopoietic progenitors from human pluripotent stem cells via respecification of lineage-restricted precursors. Cell Stem Cell *13*, 459–470.

Eilken, H.M., Nishikawa, S.-I., and Schroeder, T. (2009). Continuous single-cell imaging of blood generation from haemogenic endothelium. Nature *457*, 896–900.

Elcheva, I., Brok-Volchanskaya, V., Kumar, A., Liu, P., Lee, J.H., Tong, L., Vodyanik, M., Swanson, S., Stewart, R., Kyba, M., et al. (2014). Direct induction of haematoendothelial programs in human pluripotent stem cells by transcriptional regulators. Nat. Commun. *5*, 1–11.

Gazit, R., Garrison, B.S., Rao, T.N., Shay, T., Costello, J., Ericson, J., Kim, F., Collins, J.J., Regev, A., Wagers, A.J., et al. (2013). Transcriptome analysis identifies regulators of hematopoietic stem and progenitor cells. Stem Cell Reports *1*, 266–280.

Heinz, N., Schambach, A., Galla, M., Maetzig, T., Baum, C., Loew, R., and Schiedlmeier, B. (2011). Retroviral and transposon-based tet-regulated all-in-one vectors with reduced background expression and improved dynamic range. Hum. Gene Ther. *22*, 166–176.

Hoffmann, D., Schott, J.W., Geis, F.K., Lange, L., Müller, F.-J., Lenz, D., Zychlinski, D., Steinemann, D., Morgan, M., Moritz, T., et al. (2017). Detailed comparison of retroviral vectors and promoter configurations for stable and high transgene expression in human induced pluripotent stem cells. Gene Ther. *24*, 298–307.

Kataoka, H., Hayashi, M., Nakagawa, R., Tanaka, Y., Izumi, N., Nishikawa, S., Jakt, M.L., Tarui, H., and Nishikawa, S. (2011). Etv2/ER71 induces vascular mesoderm from Flk1 + PDGFR α + primitive mesoderm. Blood *118*, 6975–6987.

Kennedy, M., Awong, G., Sturgeon, C.M., Ditadi, A., LaMotte-Mohs, R., Zúñiga-Pflücker, J.C., and Keller, G. (2012). T lymphocyte potential marks the emergence of definitive hematopoietic progenitors in human pluripotent stem cell differentiation cultures. Cell Rep. *2*, 1722–1735.

Lammerts van Bueren, K., and Black, B.L. (2012). Regulation of endothelial and hematopoietic development by the ETS transcription factor Etv2. Curr. Opin. Hematol. *19*, 199–205.

Lancrin, C., Sroczynska, P., Stephenson, C., Allen, T., Kouskoff, V., and Lacaud, G. (2009). The haemangioblast generates haematopoietic cells through a haemogenic endothelium stage. Nature *457*, 892–895.

Lawrence, H.J., Christensen, J., Fong, S., Hu, Y.L., Weissman, I., Sauvageau, G., Humphries, R.K., and Largman, C. (2005). Loss of expression of the Hoxa-9 homeobox gene impairs the proliferation and repopulating ability of hematopoietic stem cells. Blood *106*, 3988–3994.

Lee, D., Park, C., Lee, H., Lugus, J.J., Kim, S.H., Arentson, E., Chung, Y.S., Gomez, G., Kyba, M., Lin, S., et al. (2008). ER71 acts downstream of BMP, Notch, and Wnt signaling in blood and vessel progenitor specification. Cell Stem Cell *2*, 497–507.

Ling, K.-W., Ottersbach, K., van Hamburg, J.P., Oziemlak, A., Tsai, F.-Y., Orkin, S.H., Ploemacher, R., Hendriks, R.W., and Dzierzak, E. (2004). GATA-2 plays two functionally distinct roles during the ontogeny of hematopoietic stem cells. J. Exp. Med. *200*, 871–882.

Liu, F., Li, D., Yu, Y.Y.L., Kang, I., Cha, M.-J., Kim, J.Y., Park, C., Watson, D.K., Wang, T., and Choi, K. (2015). Induction of hematopoietic and endothelial cell program orchestrated by ETS transcription factor ER71/ETV2. EMBO Rep. *16*, 654–669.

Loughran, S.J., Kruse, E.A., Hacking, D.F., de Graaf, C.A., Hyland, C.D., Willson, T.A., Henley, K.J., Ellis, S., Voss, A.K., et al. (2008). The transcription factor Erg is essential for definitive hematopoiesis and the function of adult hematopoietic stem cells. Nat. Immunol. *9*, 810–819.

Mead, P.E., Deconinck, A.E., Huber, T.L., Orkin, S.H., and Zon, L.I. (2001). Primitive erythropoiesis in the Xenopus embryo: the

synergistic role of LMO-2, SCL and GATA-binding proteins. Development *128*, 2301–2308.

Medvinsky, A., and Dzierzak, E. (1996). Definitive hematopoiesis is autonomously initiated by the AGM region. Cell *86*, 897–906.

Neehus, A.L., Lam, J., Haake, K., Merkert, S., Schmidt, N., Mucci, A., Ackermann, M., Schubert, M., Happle, C., Kühnel, M.P., et al. (2017). Impaired IFNγ-signaling and mycobacterial clearance in IFNγR1-deficient human iPSC-derived macrophages. Stem Cell Reports *10*, 7–16.

Notta, F., Zandi, S., Takayama, N., Dobson, S., Gan, O.I., Wilson, G., Kaufmann, K.B., Mcleod, J., Laurenti, E., Dunant, C.F., et al. (2015). Distinct routes of lineage development reshape the human blood hierarchy across ontogeny. Science *351*, 1–16.

Org, T., Duan, D., Ferrari, R., Montel-hagen, A., Van Handel, B., Marc, A., Sasidharan, R., Rubbi, L., Fujiwara, Y., Pellegrini, M., et al. (2015). Scl binds to primed enhancers in mesoderm to regulate hematopoietic and cardiac fate divergence. EMBO J. *34*, 759–777.

Palis, J., Robertson, S., Kennedy, M., Wall, C., and Keller, G. (1999). Development of erythroid and myeloid progenitors in the yolk sac and embryo proper of the mouse. Development *126*, 5073–5084.

Pereira, C.F., Chang, B., Qiu, J., Niu, X., Papatsenko, D., Hendry, C.E., Clark, N.R., Nomura-Kitabayashi, A., Kovacic, J.C., Ma'Ayan, A., et al. (2013). Induction of a hemogenic program in mouse fibroblasts. Cell Stem Cell *13*, 205–218.

Porcher, C., Swat, W., Rockwell, K., Fujiwara, Y., Alt, F.W., and Orkin, S.H. (1996). The T cell leukemia oncoprotein SCL/tal-1 is essential for development of all hematopoietic lineages. Cell *86*, 47–57.

Rasmussen, T.L., Kweon, J., Diekmann, M.a, Belema-Bedada, F., Song, Q., Bowlin, K., Shi, X., Ferdous, A., Li, T., Kyba, M., et al. (2011). ER71 directs mesodermal fate decisions during embryogenesis. Development *138*, 4801–4812.

Riddell, J., Gazit, R., Garrison, B.S., Guo, G., Saadatpour, A., Mandal, P.K., Ebina, W., Volchkov, P., Yuan, G.-C., Orkin, S.H., et al. (2014). Reprogramming committed murine blood cells to induced hematopoietic stem cells with defined factors. Cell *157*, 549–564.

Sandler, V.M., Lis, R., Liu, Y., Kedem, A., James, D., Elemento, O., Butler, J.M., Scandura, J.M., and Rafii, S. (2014). Reprogramming human endothelial cells to haematopoietic cells requires vascular induction. Nature *511*, 312–318.

Schwarzer, A., Emmrich, S., Schmidt, F., Beck, D., Ng, M., Reimer, C., Adams, F.F., Grasedieck, S., Witte, D., Käbler, S., et al. (2017). The non-coding RNA landscape of human hematopoiesis and leukemia. Nat. Commun. *8*, 218.

Shi, X., Richard, J., Zirbes, K.M., Gong, W., Lin, G., Kyba, M., Thomson, J.A., Koyano-Nakagawa, N., and Garry, D.J. (2014). Cooperative interaction of Etv2 and Gata2 regulates the development of endothelial and hematopoietic lineages. Dev. Biol. *389*, 208–218.

Stanulović, V.S., Cauchy, P., Assi, S.A., and Hoogenkamp, M. (2017). LMO2 is required for TAL1 DNA binding activity and initiation of definitive haematopoiesis at the haemangioblast stage. Nucleic Acids Res. *45*, 9874–9888.

Sturgeon, C.M., Ditadi, A., Awong, G., Kennedy, M., and Keller, G. (2014). Wnt signaling controls the specification of definitive and primitive hematopoiesis from human pluripotent stem cells. Nat. Biotechnol. *32*, 554–561.

Sugimura, R., Jha, D.K., Han, A., Soria-Valles, C., da Rocha, E.L., Lu, Y.-F., Goettel, J.A., Serrao, E., Rowe, R.G., Malleshaiah, M., et al. (2017). Haematopoietic stem and progenitor cells from human pluripotent stem cells. Nature *545*, 432–438.

Vo, L.T., Kinney, M.A., Liu, X., Zhang, Y., Barragan, J., Sousa, P.M., Jha, D.K., Han, A., Cesana, M., Shao, Z., et al. (2018). Regulation of embryonic haematopoietic multipotency by EZH1. Nature *553*, 506–510.

Vodyanik, M.A., Thomson, J.A., and Slukvin, I.I. (2006). Leukosialin (CD43) defines hematopoietic progenitors in human embryonic stem cell differentiation cultures. Blood *108*, 2095–2105.

Yamada, Y., Pannell, R., Forster, A., and Rabbitts, T.H. (2000). The oncogenic LIM-only transcription factor Lmo2 regulates angiogenesis but not vasculogenesis in mice. Proc. Natl. Acad. Sci. U S A *97*, 320–324.

Zhou, Y., Zhang, Y., Chen, B., Dong, Y., Zhang, Y., Mao, B., Pan, X., Lai, M., Chen, Y., Bian, G., et al. (2019). Overexpression of GATA2 enhances development and maintenance of human embryonic stem cell-derived hematopoietic stem cell-like progenitors. Stem Cell Reports *13*, 31–47.

Zovein, A.C., Hofmann, J.J., Lynch, M., French, W.J., Turlo, K.A., Yang, Y., Becker, M.S., Zanetta, L., Dejana, E., Gasson, J.C., et al. (2009). Fate tracing reveals the endothelial origin of hematopoietic stem cells. Cell Stem Cell *3*, 625–636.

Stem Cell Reports, Volume *14*

Supplemental Information

Inducible Forward Programming of Human Pluripotent Stem Cells to Hemato-endothelial Progenitor Cells with Hematopoietic Progenitor Potential

Lucas Lange, Dirk Hoffmann, Adrian Schwarzer, Teng-Cheong Ha, Friederike Philipp, Daniela Lenz, Michael Morgan, and Axel Schambach

Inventory of Supplemental Information

- Figure S1 Characterization of the Dox-inducible vector-system and SLGE-iPSC lines. Related to Figure 1
- Figure S2 Kinetic analysis of immunophenotype during Phase I of hemato-endothelial forward programming. Related to Figure 2
- Figure S3 Vascular endothelial potential and reproducibility of the forward programming protocol. Related to Figure 3
- Figure S4 Characterization of the HPC potential and function. Related to Figure 5
- Figure S5 Sorting strategy and gating scheme for the RNA-sequencing approach. Related to Figure 6
- Figure S6 RNA-sequencing analysis of SLGE-iPSC derived HEP/HPC and cord blood HSC. Related to Figure 6
- Supplemental experimental procedures
    - Table S1: Flow Cytometry Antibody
    - Table S2: Primer/Oligonucleotide
- Supplemental References
- Table S3: Gene Sets for Gene Set Enrichment Analysis (separate file)

Figure S1 Characterization of the Dox-inducible vector-system and SLGE-iPSC lines. Related to Figure 1

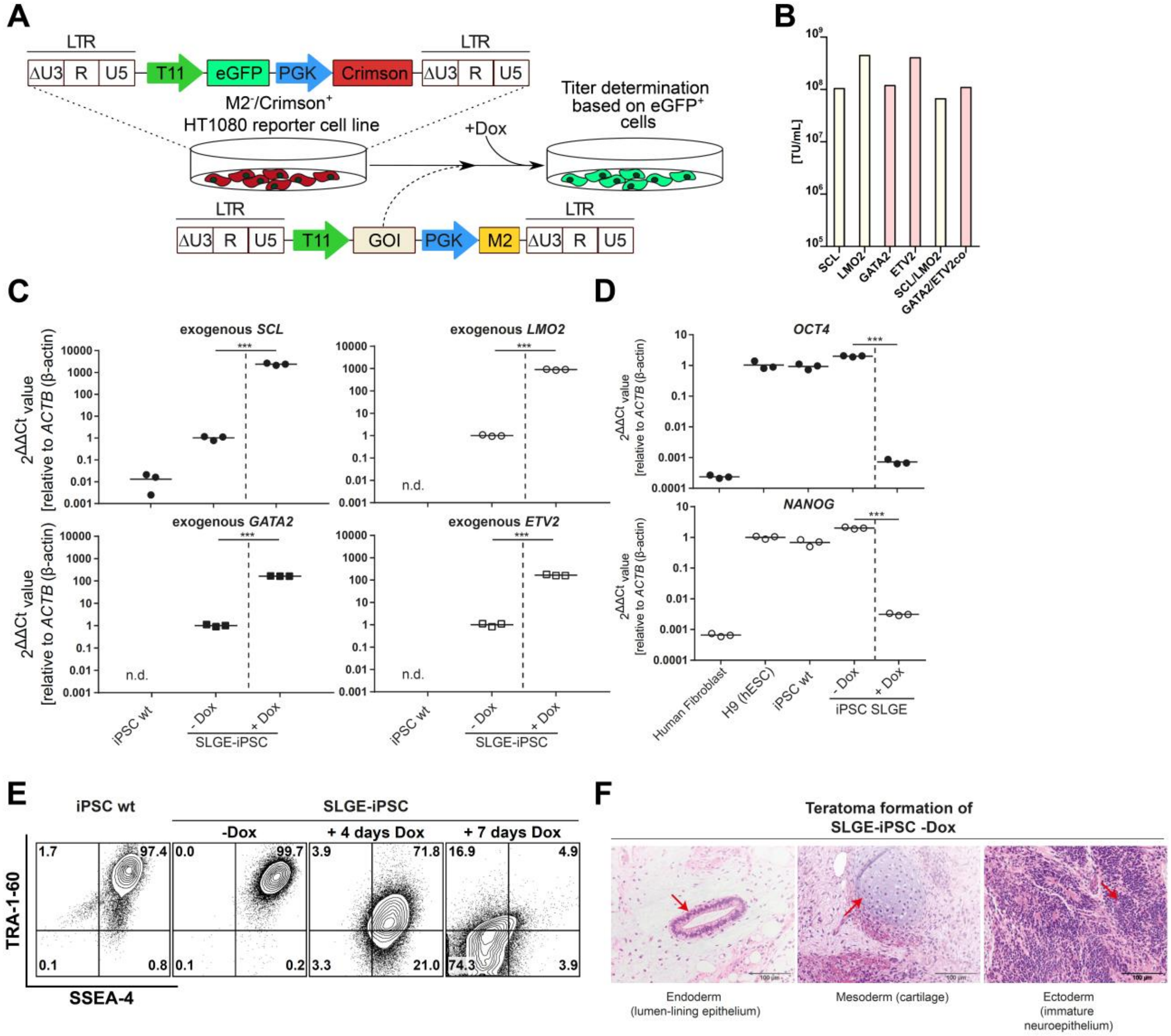

Figure S1 Characterization of the Dox-inducible vector-system and SLGE-iPSC lines. Related to Figure 1. (A) HT1080 reporter cell line for titration of all-in-one Dox-inducible vectors. Reporter contains an eGFP cassette under the control of a Dox-inducible T11 promoter and detects the biological activity of the M2 transactivator of the all-in-one vectors upon successful transduction and transgene expression. **(B)** Titer (Transducing units (TU) per mL) of all-in-one TF expressing vectors. **(C)** Inducibility of ectopic SLGE expression upon Dox stimulation, determined by quantitative RT-PCR. Cells were stimulated for 7 days with Dox (+Dox). Expression was normalized to –Dox control (basal expression), n.d. = not detected; samples were measured in triplicates. P-values were calculated using one-way ANOVA with Bonferroni's Multiple Comparison Test. **(D)** Comparison of endogenous expression level of human pluripotency genes *OCT4* and *NANOG* (determined by quantitative RT PCR) between wild type iPSC (wt) and SLGE-iPSC (-/+ Dox). Human *ACTB* (β-Actin) was used as housekeeping gene, and expression levels were normalized to H9 hESC as a pluripotency control in triplicate samples (mean of replicates). P-values were calculated using one-way ANOVA with Tukey's multiple comparisons test. **(E)** Expression of pluripotency surface markers TRA-1-60 and SSEA-4 on H2E6C wild type iPSC and H2E6C SLGE iPSC (-/+ Dox) in iPSC-maintenance culture (gated on unstained control). **(F)** Representative H&E staining of teratoma showing cells derived from all three germ-layers after injection of SLGE iPSC (Dox) into immunocompromised NSGS mice (scale bar 100 µm).

Figure S2 Kinetic analysis of immunophenotype during Phase I of hemato-endothelial forward programming. Related to Figure 2

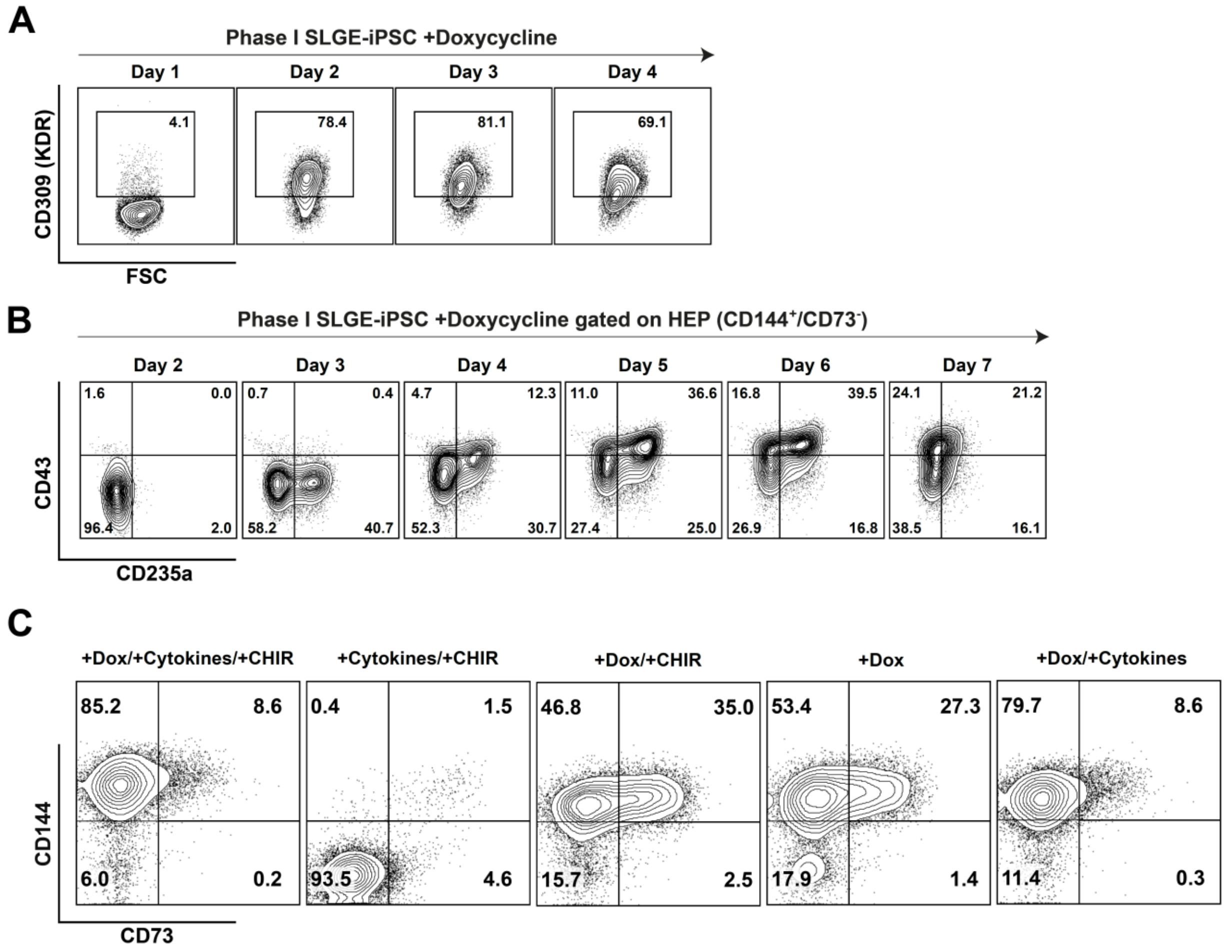

Figure S2 Kinetic analysis of immunophenotype during Phase I of hemato-endothelial forward programming. Related to Figure 2 (A) Kinetic FCM analysis of the mesodermal priming based on the expression of the vascular endothelial growth factor receptor 2 (KDR) during the first four days of Phase I. **(B)** Kinetic analysis of the immunophenotype of CD43/CD235a expression during the seven days of Phase I in CD144$^+$/CD73$^-$ SLGE-HEP (+Dox). **(C)** Influence of SLGE induction (+Dox), Cytokines and small molecule (CHIR) on the hemato-endothelial specification (Phase I day 7, CD144$^+$/CD73$^-$). Cytokines (SCF, FGF2, TPO, VEGF and IL-3) and/or small molecule (CHIR) were removed during Phase I to evaluate the effects of the components on hemato-endothelial specification. Gates were set based upon FMO controls.

Figure S3 Vascular endothelial potential and reproducibility of the forward programming protocol. Related to Figure 3

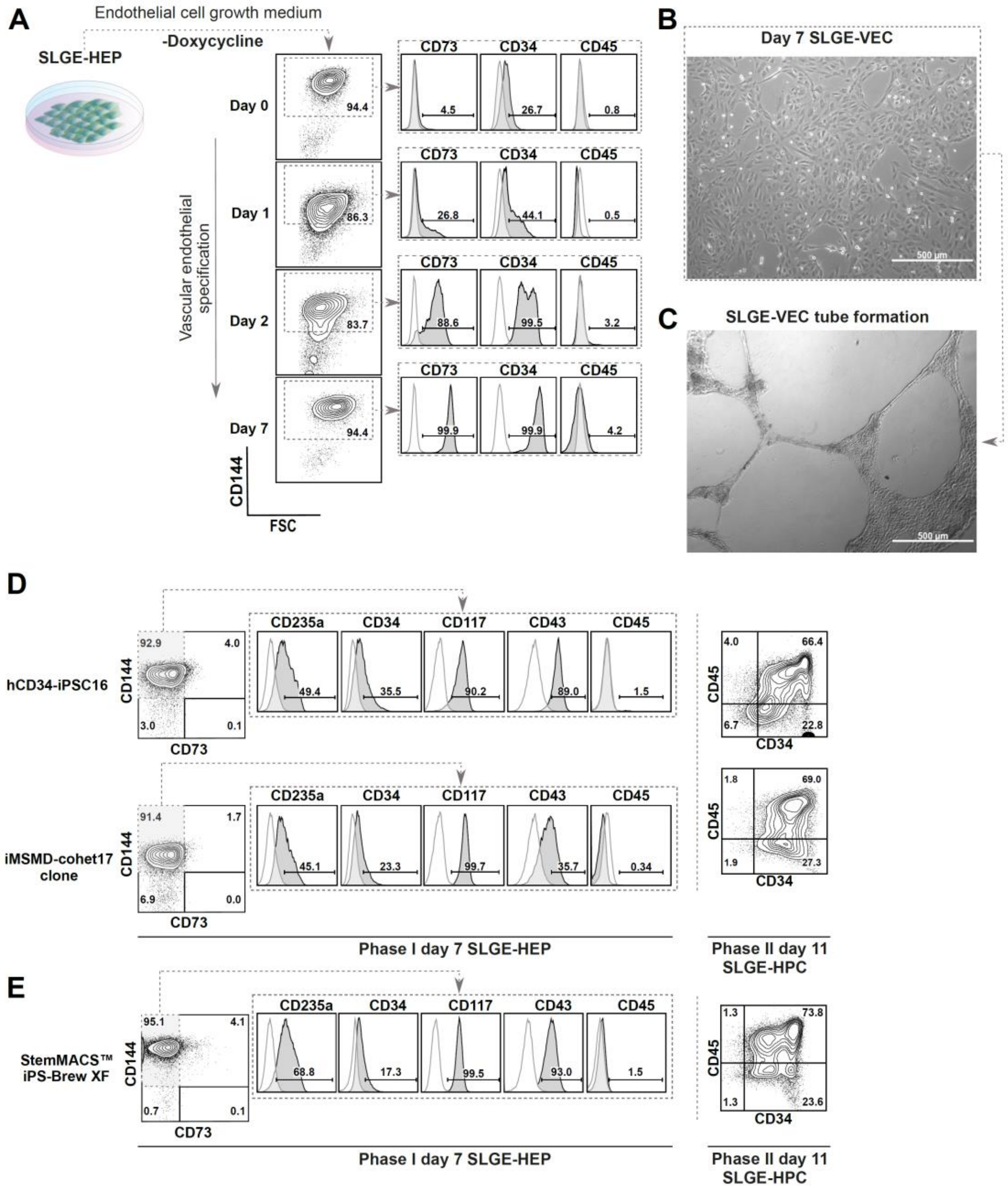

Figure S3 Vascular endothelial potential and reproducibility of the forward programming protocol. Related to Figure 3. (A) Vascular endothelial potential of Phase I day 7 SLGE-HEP. Cells were cultivated for 7 days in EGM2 (endothelial growth medium) without Dox. Kinetic of vascular endothelial specification with upregulation of endothelial-specific surface marker ($CD144^{+}/CD73^{+}/CD34^{+}$) within 7 days (SLGE-VEC) without upregulation of hematopoietic marker CD45. **(B)** Representative microscopy image of SLGE-VEC (7 days in EGM2) (Scale bar 500 µm). **(C)** Tubular-like structures of SLGE-VEC in an *in vitro* angiogenesis assay on gelled basement matrix (Scale bar 500 µm). **(D)** Hemato-endothelial differentiation of different SLGE-iPSC lines. hCD34-iPSC16 iPSC ($CD34^{+}$ cell-derived iPSC) and iMSMD-cohet 17 iPSC (peripheral blood-derived iPSC). Both lines exhibited similar hemato-endothelial differentiation potential and produced SLGE-HEP (Phase I day 7) as well as SLGE-HPC (Phase II day 11) **(E)** Hemato-endothelial differentiation potential of SLGE-iPSC Phase I day 7 SLGE-HEP and Phase II day 11 SLGE-iPSC cultivated in fully defined xeno-free StemMACS iPS-Brew medium.

Figure S4 Characterization of the HPC potential and function. Related to Figure 5

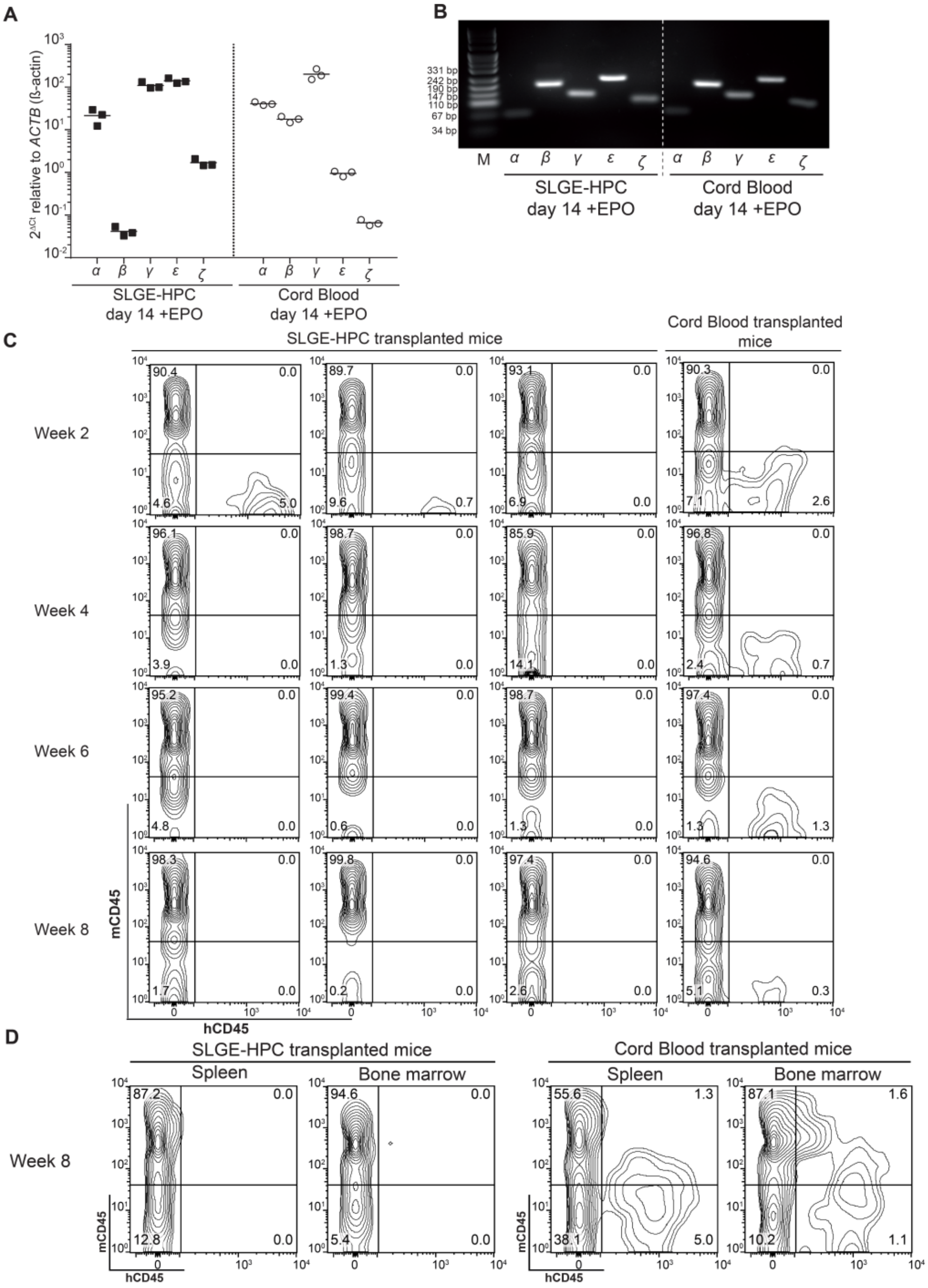

Figure S4 Characterization of the HPC potential and function. Related to Figure 5 (A) Globin Expression profile (embryonic isoform ζ- (*HBZ*) and ε- (*HBE*), the fetal isoform γ- (*HBG*) and α-globin (*HBA*) and the adult type β- (*HBB*)) of SLGE-HPC and cord blood cells (differentiated for 14 day in EPO containing medium) determined by quantitative RT-PCR in triplicate samples. Human *ACTB* (β-actin) was used as housekeeping control and $2^{\Delta CT}$ calculation. **(B)** Gel electrophoresis of the RT-qPCR products of the globin expression profile demonstrated the specificity of the RT-qPCR reactions. **(C)** Analysis of peripheral blood of either cord blood or SLGE-HPC (n=3) transplanted immunodeficient NSGS mice at 2, 4, 6 and 8 weeks post-transplantation for the presence of $hCD45^+$ hematopoietic cells. **(D)** Representative analysis of the spleen or the bone marrow of either cord blood or SLGE-HPC (n=3) transplanted immunodeficient NSGS mice 8 weeks post-transplantation for the presence of $hCD45^+$ cells.

Figure S5 Sorting strategy and gating scheme for the RNA-sequencing approach. Related to Figure 6

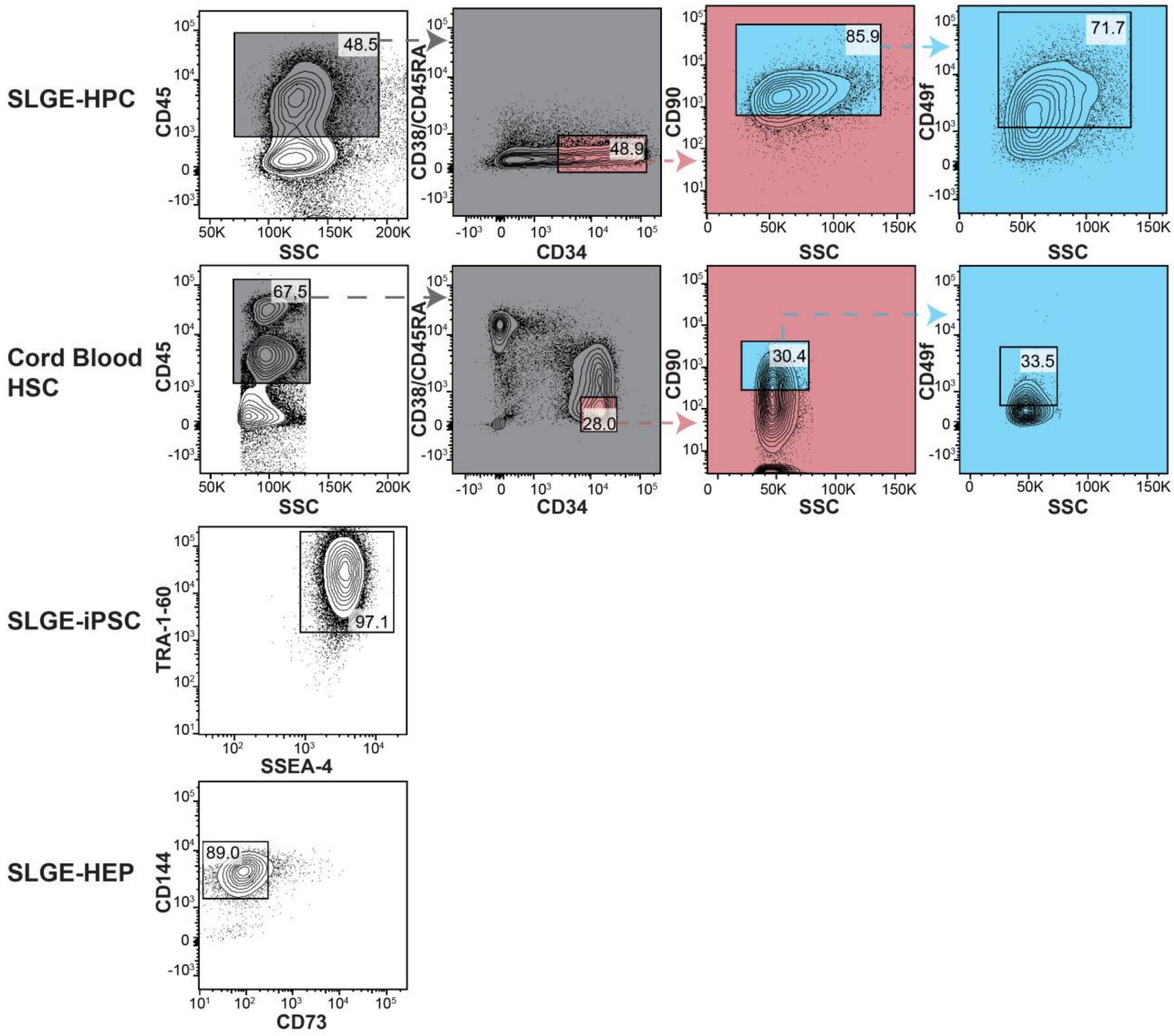

Figure S5 Sorting strategy and gating scheme for the RNA-sequencing approach. Related to Figure 6 Fluorescence activated cell sorting strategy for the RNA-sequencing comparison of SLGE-HPC ($CD45^+/CD38^-/CD45RA^-/CD34^{high}/CD90^{high}/CD49f^{high}$), human umbilical cord blood HSC ($CD45^+/CD38^-/CD45RA^-/CD34^{high}/CD90^{high}/CD49f^{high}$), SLGE-iPSC ($TRA\text{-}1\text{-}60^+/SSEA\text{-}4^+$) and SLGE-HEP ($CD144^+/CD73^-$).

Figure S6 RNA-sequencing analysis of SLGE-iPSC derived HEP/HPC and cord blood HSC. Related to Figure 6

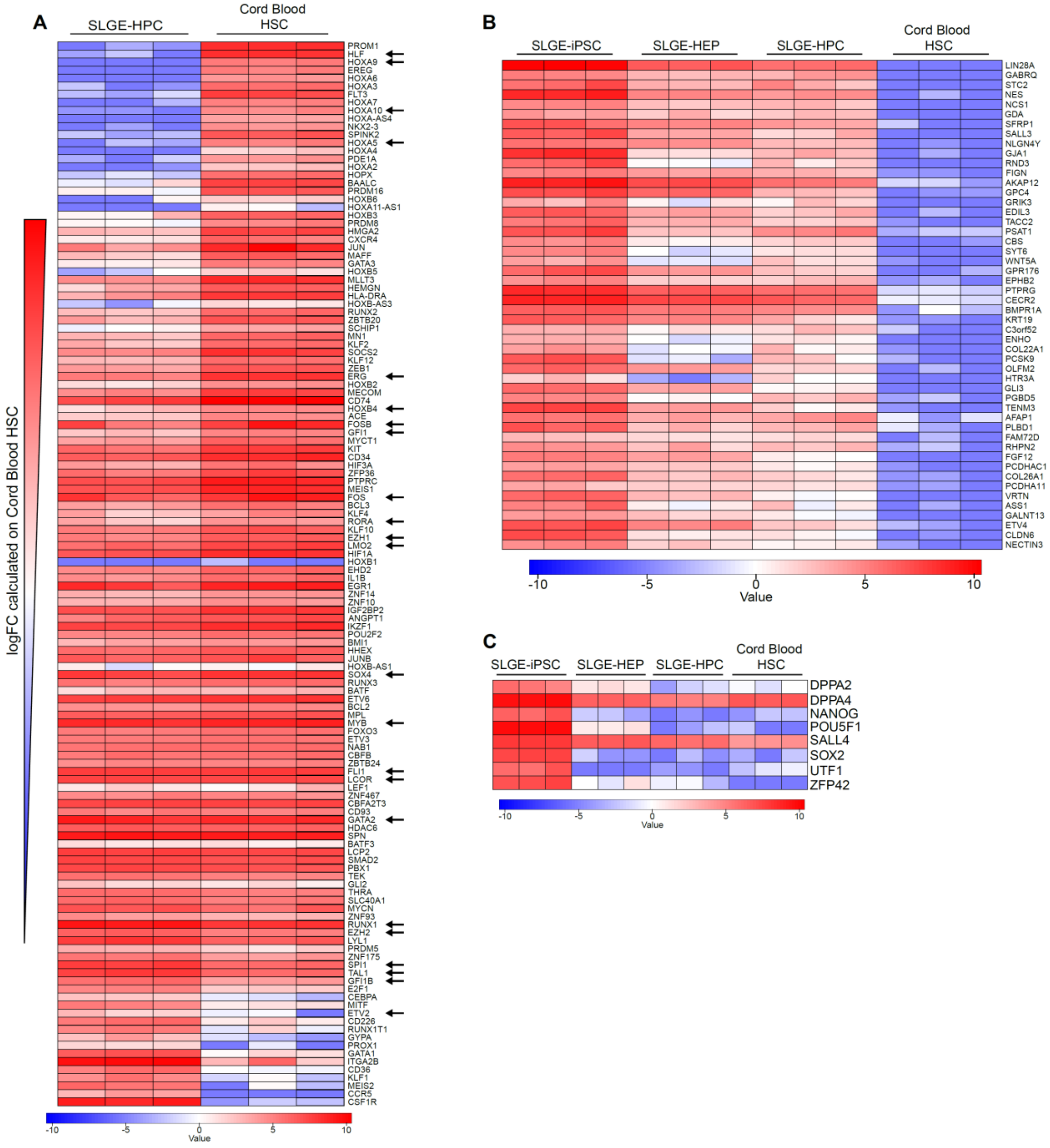

Figure S6 RNA-sequencing analysis of SLGE-iPSC derived HEP/HPC and cord blood HSC. Related to Figure 6 (A) Analysis of differentially or similarly expressed genes associated with hematopoietic development between cord blood HSC and SLGE-HPC. The genes were ranked over the highest logarithmic fold change (logFC) calculated on cord blood HSC. Gene ontology was done using DAVID. Black arrows indicate transcription factors that were previously used in transdifferentiation (Batta et al., 2014; Doulatov et al., 2013; Gomes et al., 2018; Pereira et al., 2013; Riddell et al., 2014; Sandler et al., 2014; Sugimura et al., 2017) and forward programming (Elcheva et al., 2014) approaches. **(B)** Heatmap of log-CPM values for the top 50 of not silenced genes of SLGE-iPSC, SLGE-HEP and SLGE-HPC *(mean $log_2FC=7.6$, $P_{adj} < 0.03$)* during hemato-endothelial differentiation compared to cord blood HSC. **(C)** Heatmap of log-CPM values for genes associated with pluripotency and stemness.

Supplemental Experimental Procedures

Cell culture

Human iPSC were cultivated in conditioned iPSC medium, composed of Dulbecco's modified Eagle's medium/F12 GlutaMAX with 20% knockout serum replacement (both Gibco, Karlsruhe, Germany), 100 U mL^{-1} penicillin, 100 µg mL^{-1} streptomycin (PAN Biotech), 2 mM L-glutamine (Merck Millipore, Darmstadt, Germany), 1% non-essential amino acids (Gibco), 100 µM β-mercaptoethanol (Sigma-Aldrich) (CM; without bFGF cultured for 24h on γ-irradiated C3H mouse embryonic fibroblasts, harvested, filtered and optionally stored at -20°C) as a feeder-free monolayer (ML) using Geltrex (Gibco) coated cell culture dishes. Fresh FGF2 (30 ng mL^{-1}) was supplemented before usage. Long term iPSC cultivation was performed as ML in CM in the presence of puromycin (0.3 ng mL^{-1}) and/or zeocin (0.5 ng mL^{-1}) (both InvivoGen, Toulouse, France) to prevent vector-silenced SLGE-iPSC throughout the cultivation. Alternatively, iPSC were cultivated and maintained in fully defined StemMACS™ iPS-Brew XF stem cell medium (Miltenyi Biotec, Bergisch Gladbach, Germany). Human SLGE-iPSC were harvested with Accutase (Thermo Fisher, Darmstadt, Germany) and split twice a week at a density of 1×10^6 cells per 9 cm² onto new Geltrex-coated plates. ROCK-inhibitor (Y-27632, 10 µM) (kindly provided by T. Scheper, Leibniz University Hannover) was applied to the SLGE-iPSC culture at least 1h before Accutase treatment and into the fresh iPSC medium.

Lentiviral particle production

Lentiviral particles were produced by calcium phosphate transfection, as previously described (Schambach et al., 2006) and vesicular stomatitis virus envelope glycoprotein (VSV-G) pseudotyped. Lentiviral supernatants were harvested 36 and 48 hours post-transfection, filtered, pooled and concentrated (100-fold) via ultracentrifugation for 2 hours at 82 740 × g (rotor SW32Ti, Beckman Coulter, Krefeld, Germany), 4 °C and stored at – 80°C.

Transduction and antibiotic selection of stable induced pluripotent stem cells

Transductions with the two vectors SL and GE were performed sequentially in suspension to establish double-positive pluripotent stem cell lines (SLGE). Single-cell suspensions of 2.5×10^4 cells were transduced with MOI=0.5 for SL and MOI=10 for GE in 100 µL iPSC-medium for 1h at 37°C and 5% CO_2 in the presence of 4 µg mL^{-1} protamine sulfate (Sigma-Aldrich) and 10 µM Y-27632. Cells were kept in suspension by flicking the tube every 10 minutes and seeded in iPSC medium containing Y-27632 on Geltrex-coated cell culture dishes afterward. Medium was exchanged the following day and every other day. Puromycin (0.3 g mL^{-1}) and zeocin (0.5 µg mL^{-1}) (both InvivoGen) were applied 24 h post-transduction.

Vascular endothelial differentiation of SLGE-HEP

Phase I day 7 SLGE-HEP were cultivated for 7 days in endothelial growth medium 2 (EGM2) (Lonza, Visp, Switzerland) on 0.1% gelatin (Sigma Aldrich) coated cell culture dishes. Flow cytometry was used to analyze the maturation stage over time. Flow cytometry was performed on CytoFLEX S Flow Cytometer (Beckman Coulter) using antibodies against CD144, CD73, CD34 and CD45 (Supplementary flow cytometry antibodies list). Tube-formation assay was done on undiluted Matrigel (Corning) according to previously described method (Arnaoutova and Kleinman, 2010).

Terminal differentiation of hematopoietic progenitor cells

Multipotency and proliferative potential of SLGE-HPC was assessed by colony-forming assay and directed lineage differentiation. For colony-forming assays, 5000 SLGE-HPC were seeded in Methocult H4034 (STEMCELL Technologies) according to the manufacturer's protocol. Colonies were counted and determined using standard morphological criteria after 12 to 14 days. Lineage-specific differentiation of SLGE-HPC into granulocytes was performed in IMDM (Biochrom, Berlin, Germany) supplemented with 10% heat-inactivated fetal bovine serum (FBS) (Pan Biotech), 1% bovine serum albumin (PAA, Coelbe, Germany), 0.5% non-essential amino acids, 2 mM L-glutamine, 100 U mL^{-1} penicillin, 100 µg mL^{-1} streptomycin, 100 µM β-mercaptoethanol, 100 ng mL^{-1} SCF, 25 ng mL^{-1} IL-3 and 50 ng mL^{-1} GM-CSF and 100 ng mL^{-1} G-CSF (all Peprotech). RPMI with 10% heat-inactivated FBS, 100 U mL^{-1} penicillin, 100 µg mL^{-1} streptomycin was either supplemented with 50 ng mL^{-1} M-CSF (Peprotech) for monocyte/macrophage differentiation, with 20 ng mL^{-1} SCF, 25 ng mL^{-1} IL-3 and 3 U mL^{-1} EPO for erythroid differentiation or with 50 ng mL^{-1} TPO (all Peprotech) for megakaryocytic differentiation. T- and NK-cell differentiation was performed in co-cultivation on semi-confluent OP9-Dll1 cells in RPMI supplemented with 20%

FBS, 100 U mL^{-1} penicillin, 100 µg mL^{-1} streptomycin, 10 ng mL^{-1} SCF, 5 ng mL^{-1} Flt3L, 5 ng mL^{-1} IL-7 for T-cell progenitor differentiation and additionally 10 ng mL^{-1} IL-15 (all Peprotech) for NK-specification. To assess morphology of SLGE-iPSC-derived hematopoietic lineages, 5×10^{4} cells were spun onto microscope slides using the Cytospin 4 (Thermo Fisher) and stained with May-Grünwald and Giemsa (both Sigma Aldrich), following manufacturer's instructions.

Flow Cytometry and cell sorting

Flow cytometry was performed on BD FACSCalibur (Becton Dickenson, Heidelberg, Germany) or CytoFLEX S Flow Cytometer (Beckman Coulter), sorting was performed on FACSAria Fusion (Becton Dickenson). DAPI (Sigma-Aldrich) was used at a final concentration of 200 ng mL^{-1} as viability dye. Voltage and compensation were adjusted by single stain controls or UltraComp eBeads microspheres (Thermo Fisher). Gating was performed on unstained controls or fluorescence minus one (FMO) as indicated. . All used antibodies can be found in Table S1: Flow Cytometry Antibodies.

Quantitative Reverse-Transcriptase PCR analysis

Total RNA was extracted using Qiagen RNeasy Mini or Micro Kit (Qiagen, Hilden, Germany) according to the manufacturer's protocol. RNase was inhibited using 50 U RiboLock (Thermo Fisher Scientific). cDNA synthesis and removal of genomic DNA were performed using QuantiTect Reverse Transcription Kit (Qiagen) according to the manufacturer's instructions. QuantiTect SYBR® Green RT-PCR Kit (Qiagen) was applied for quantitative PCR using the StepOnePlus Real-Time PCR System (Applied Biosystems, Darmstadt, Germany). To allow the intercomparability of different time points (Figure 4A), the point of maximum gene expression was set to one for each target. All used primers can be found in Table S2: Primer/Oligonucleotides. Expression of target genes was evaluated as ΔΔCt relative to expression of a housekeeper gene (*ACTB* (β-Actin))

Isolation of human CD34^{+} cells from umbilical cord blood

Human cord blood was obtained from the Department of Gynecology and Obstetrics at Hannover Medical School with written informed consent, according to the local ethics committee. Mononuclear cells were isolated with Leucosep separation tubes (Greiner Bio-One, Frickenhausen, Germany) according to the manufacturer's protocol using biocoll as separation solution (Biochrom). CD34^{+} selection was done using the CD34 MicroBead Kit (Miltenyi) according to the manufacturer's instructions. Positively selected cells were either directly sorted or cryostored in StemSpan (STEMCELL Technologies) with 10% DMSO.

Gene expression profiling by RNA-Sequencing

Whole transcriptome analysis was done comparing SLGE-iPSC (TRA-1-60^{+}/SSEA4^{+}), SLGE-HEP (CD144^{+}/CD73^{-}, 3 independent differentiations), SLGE-HPC (CD45^{+}/CD34^{+}/CD90^{+}/CD49f^{+}/CD38^{-}/CD45RA^{-}; 3 independent differentiations) with human umbilical cord blood HSC (CD45^{+}/CD34^{+}/CD90^{+}/CD49f^{+}/CD38^{-}/CD45RA^{-} from 3 individual preparations and sorts). All samples used for RNA-sequencing were FACS-sorted in line with a previously described sorting strategy (Notta et al., 2015) (Figure S5) All used antibodies can be found in the supplemental information in the flow cytometry antibodies list. DAPI (Sigma-Aldrich) was used at a final concentration of 200 ng mL^{-1} as viability dye. Total RNA was isolated using Qiagen RNeasy Micro Kit (Qiagen). RNA quality/integrity was assessed using an Agilent 2100 Bioanalyzer (Agilent, Waldbronn, Germany). Prior to RNA-sequencing, ribosomal RNA was removed using a NEBNext rRNA Depletion Kit (New England BioLabs, Frankfurt am Main, Germany), according to the manufacturer's protocol. 5 ng total RNA were used for library preparation with the 'SMARTer Stranded Total RNA-Seq Kit v2 – Pico Input Mammalian' (#634413; Takara, Saint-Germain-en-Laye, France) according to conditions recommended in user manual #063017. Generated libraries were barcoded by a dual indexing approach and were amplified by 11 PCR cycles. Fragment length distribution of generated libraries was monitored using 'Bioanalyzer High Sensitivity DNA Assay' (5067-4626; Agilent Technologies). Quantification of libraries was performed with the 'Qubit® dsDNA HS Assay Kit' (Q32854; ThermoFisher Scientific). Sequencing runs were performed with equal molar amounts of five (SLGE-HPC and Cord Blood HSC), or six (SLGE-iPSC and SLGE-HEP) pooled libraries for a common sequencing run. Accordingly, each analyzed library constitutes 20% (SLGE-HPC and Cord Blood HSC), or 16.6% (SLGE-iPSC and SLGE-HEP) of overall flowcell capacity. The combined library pools were denatured with sodium hydroxide and were finally diluted to 1.5 pM according to the Denature and Dilute Libraries Guide (Document # 15048776 v02; Illumina, San Diego, USA). 1.3 mL of each denatured pool was loaded on an Illumina NextSeq 550 sequencer using a High Output

Flowcell for 75 bp single reads (#FC-404-2005, Illumina). Raw data were processed by converting the BCL files into FASTQ files using bcl2fastq Conversion Software version v2.20.0.422 (Illumina). The FASTQ files were adapted and quality trimmed using Trim Galore! (Version 0.4.1) with default settings as described in the User Guide except for the setting of the quality cutoff (-q/--quality), which was set to a Phred score of 15. Trim Galore! used Cutadapt (version 1.9.1) as subroutine. Quality control of FASTQ files was performed by FastQC (version 0.11.4) before and after trimming. After trimming, FASTQ files were mapped against a reference genome with the splice-aware aligner STAR (version 2.5.0c) to generate BAM files. The BAM files were built in a 2-pass mapping (--twopassMode Basic) and were finally sorted (--outSAMtype BAM SortedByCoordinate). All other settings were left as default as described in the manual. The genome index files were created by STAR with default settings using *Homo sapiens* sequence and annotation data (UCSC, built hg19) available on Illumina's iGenome site (http://support.illumina.com/sequencing/sequencing_software/igenome.html). The average number of reads entering the mapping process across all analyzed samples was 94.4 million (SLGE-HPC and Cord Blood HSC) or 90.9 million (SLGE-iPSC and SLGE-HEP). The average percentage of uniquely mapped reads was 79.9% (SLGE-HPC and Cord Blood HSC) or 78.7% (SLGE-iPSC and SLGE-HEP). The average percentage of reads mapped to multiple loci was 5.5% (SLGE-HPC and Cord Blood HSC), or 7.1% (SLGE-iPSC and SLGE-HEP).The average percentage of unmapped reads was 14.4% (SLGE-HPC and Cord Blood HSC) or 13.8% (SLGE-iPSC and SLGE-HEP). Read counting was performed by featureCounts as part of the Subread (Liao et al., 2013) software package (version 1.6.1) with default settings except for strandedness (-s), which was set to 2, i.e. reverse, minimum mapping quality per read (-Q) was set to 10, counting primary alignments only (--primary) and counting multi-mapping reads was enabled (-M) together with fractional assignment of reads (--fractional). *Homo sapiens* annotation data (gencode.v28lift37) was used. Raw counts were filtered (counts per million (cpm) >0.6 in more than three samples, 22,214 genes after filtering) and TMM-normalized using edgeR (McCarthy et al., 2012). For visualization (PCA and heatmaps) filtered and normalized counts were transformed into log-cpm values using a prior count of 1. Differential expression analysis was performed using limma-voom (Law et al., 2014). P-values were adjusted for multiple testing using the Benjamini-Hochberg adjustment method in the limma package (Ritchie et al., 2015). Heatmaps were generated with the function heatmap.2 from the gplots package on the log-cpm values on the features/genes indicated in the figure legends. Principal component analysis (PCA, Figure 6A) was performed on log-CPM values for all 22,214 genes. For Gene Set Enrichment analysis gene-lists for the contrasts of interest (CB-HSC vs SLGE-HPC) were generated using the limma "topTable" function. This assigns to each of the 22,214 unique genes a log-FC and a p-value for the given contrast. The gene-lists were sorted by decreasing log-FC, filtered for protein-coding genes using the GenCode V28 annotation and submitted to the Broad GSEA tool (Subramanian et al., 2005) using the GSEA pre-ranked function with the gene set permutations set to 1000. In addition to the gene set collections from MSigDB v6.2 (Subramanian et al., 2005), 130 hematopoiesis-related gene sets were tested (Schwarzer et al., 2017).

Mouse transplantation and teratoma assay

All animal experiments were approved by the animal welfare committee of Lower Saxony and were performed in accordance with institutional guidelines of Hannover Medical School. Mice were bred and housed in pathogen-free environment in the animal facility of Hannover Medical School. Transplantations were conducted by intravenous tail-vein injection into 5 weeks old NSGS mice (NOD.Cg-Prkdcscid Il2rgtm1Wjl Tg(CMV-IL3,CSF2,KITLG)1Eav/MloySzJ). Before transplantation, mice were sublethally irradiated with 1.9 Gy using GammaCell2000 (Cs_{137}) (Nuklear Data, Frankfurt, Germany). Transplantations were conducted either with SLGE-HPC (1.7×10^6 cells/mice in 150 µL total injection volume) or expanded human $CD34^+$ cord blood cells (8×10^5 cells/mice in 150 µL total injection volume) as positive control. Expansion of cord blood-derived $CD34^+$ cells was performed in StemSpan (STEMCELL Technologies), supplemented with 100 ng ml^{-1} SCF, 50 ng ml^{-1} TPO, 100 ng ml^{-1} Flt3L, 35 nM UM171 (ApexBio, Houston, USA), 500 nM StemReginin 1 (STEMCELL Technologies). Blood sampling was performed by puncture of retro-orbital plexus 2, 4, 6 and 8 weeks post-transplantation. Total chimerism was analyzed 8 weeks post-transplantation in peripheral blood, spleen and bone marrow by flow cytometry with antibodies against human CD45-APC (clone: 2D1, Thermo Fisher) and anti-murine-CD45-PE (Clone 30F11, Miltenyi Biotec). For teratoma formation, H2E6C SLGE-iPSC were harvested in the presence of 10 µM Y-27632 by TrypLE Select (Thermo Fisher Scientific) treatment. Cell suspension was mixed 1:1 with undiluted Matrigel (Corning, Kaiserslautern, Germany). 3×10^6 SLGE-iPSC in 200 µL were subcutaneously injected into each flank of adult NSGS mice, as described previously (Philipp et al., 2018). After tumor formation (41 days), teratomas were fixed in 4% formaldehyde (Carl Roth, Karlsruhe, Germany) and embedded in paraffin. 3 µm thick tissue slices were stained with hematoxylin and eosin according to standard protocols. Determination of germ layers was accomplished with an Olympus system (microscope BX51, camera XC50, software Cell^F 3.4).

Statistics

All statistical analyses were done using GraphPad Prism. Two-tailed, unpaired *t*-test (normal distribution assumption) was used for comparison between two groups. One-way ANOVA was used for statistical comparison for more than two groups, as indicated. P-values > 0.05 were considered not significant (ns). P-values ≤ 0.05 (*) were considered significant, p-values ≤ 0.01 (**) were considered very significant, and p-values ≤ 0.001 (***) were considered extremely significant.

Table S1: Flow Cytometry Antibodies

Antibody	Fluorophore	Reactivity	Dilution	Used in Figure	Supplier	Cat.No.
**Anti SSEA-4**	Alexa Fluor 647	Human	1:25	S1E/S5	BD Bioscience	560796
**Anti Tra-1-60**	PE	Human	1:25	S1E/S5	Miltenyi Biotec	130-100-347
**CD117**	PE	Human	1:25	2F/S3D/S3E	Miltenyi Biotec	130-091-734
**CD11b**	PE	Human+Mouse	1:25	5A	Miltenyi Biotec	130-091-240
**CD14**	Alexa Fluor 700	Human	1:25	5A	Biolegend	367114
**CD144**	FITC	Human	1:25	1B/1C/2A/2F/3B/S2C/S3A/S3D/S3E/S5	BD Bioscience	560411
**CD15**	FITC	Human	1:25	5A	Miltenyi Biotec	130-098-013
**CD16**	PE-Vio770	Human	1:25	5A	Miltenyi Biotec	130-096-655
**CD163**	PerCP	Human	1:25	5A	BioLegend	333626
**CD209**	PE-Cy7	Human	1:25	5A	BioLegend	330114
**CD235a**	APC-C7	Human	1:25	2F/S2B/3E/S3D/S3E/5A	Biolegend	349116
**CD309**	PE	Human	1:25	S2A	BD Bioscience	560494
**CD34**	PE-Cy7	Human	1:25	2F/S3A/S3D/S3E	BD Bioscience	348811
**CD34**	APC	Human	1:25	3B/3E/S5	BD Bioscience	345804
**CD38**	FITC	Human	1:25	3E/S5	eBioscience	11-0388-42
**CD41a**	APC	Human	1:25	5A	Miltenyi Biotec	130-105-613
**CD42b**	PE	Human	1:25	5A	Biolegend	303906
**CD43**	PerCp-eFluor710	Human	1:25	2F/S2B/3E/S3D/S3E	eBioscience	46-0438-42
**CD45**	BV650	Human	1:25	2F/5A/3E/S3A/S3D/S3E/S5	BD Bioscience	563717
**CD45**	PE	Human	1:25	3B	Miltenyi Biotec	130-080-201
**CD45**	APC	Human	1:25	S4C	eBioscience	17-9459-42
**CD45**	PE	Mouse	1:25	S4C	Miltenyi Biotec	130-117-348
**CD45RA**	FITC	Human	1:25	3E/S5	Miltenyi Biotec	130-108-713
**CD49f**	PE	Human+Mouse	1:100	3E/S5	BioLegend	313612
**CD56**	PE	Human	1:25	5A	BD Bioscience	555516
**CD61**	PerCP	Human	1:25	5A	BD Bioscience	564173
**CD66b**	PE	Human	1:25	5A	BD Bioscience	561650
**CD73**	APC	Human	1:25	1B/1C/2A/2F/S2C/S3A	Miltenyi Biotec	130-097-945
**CD73**	PE	Human	1:50	S5	Miltenyi Biotec	130-095-182
**CD86**	APC	Human	1:25	5A	Biolegend	305412
**CD90**	PE-Cy7	Human	1:25	3E/S5	Biolegend	328123

Table S2: Primer/Oligonucleotides

Primer ID	Primer sequences 5’ → 3'	Amplicon length	Used in Figure	Reference
OCT4 Forward	CCTCACTTCACTGCACTGTA	164 bp	1B/4A	(Schott et al., 2014)
OCT4 Reverse	CAGGTTTTCTTTCCCTAGCT			
NANOG Forward	TCACACGGAGACTGTCTCTC	169 bp	1B	(Schott et al., 2014)
NANOG Reverse	GAACACAGTTCTGGTCTTCTG			
β-ACTIN Forward	CCTCCCTGGAGAAGAGCTA	109 bp	1B/1D/4A /4B/S4A	(Galla et al., 2011)
β-ACTIN Reverse	TCCATGCCCAGGAAGGAAG			
exo *SCL* Forward	GACAGCTACACGGAGGAGC	126 bp	1D/4B	-
exo *SCL* Reverse	GCCACAGGTCAGCAGAGAG			
exo *LMO2* Forward	GCTCTCTGCTGACCTGTGG	82 bp	1D/4B	-
exo *LMO2* Reverse	GGGTCCAGGCTCTTCCTTTC			
exo *GATA2* Forward	TCCTCCAGCCTCTCCTTCG	91 bp	1D/4B	-
exo *GATA2* Reverse	GCTTCAACAGGGCGTAGTTTGT			
exo *ETV2co* Forward	TGGACCGATATGGCCTGTA	101 bp	1D/4B	-
exo *ETV2co* Reverse	GATCCAGCGGCAGGAATAG			
TBXT Forward	TGAGCCTCGAATCCACATAGTG	121 bp	4A	-
TBXT Reverse	AAGAGCTGTGATCTCCTCGT			
KDR Forward	AGCAGGATGGCAAAGACTAC	116 bp	4A	(Gao et al., 2017)
KDR Reverse	TACTTCCTCCTCCTCCATACAG			
RUNX1 Forward	ACTCGGCTGAGCTGAGAAATG	140 bp	4A	(Challen and Goodell, 2010)
RUNX1 Reverse	GACTTGCGGTGGGTTTGTG			
RUNX1c Forward	TGGTTTTCGCTCCGAAGGT	130 bp	4A	(Challen and Goodell, 2010)
RUNX1c Reverse	CATGAAGCACTGTGGGTACGA			
CDH5 Forward	GCAGCAGCAGGTGCTAACC	203 bp	4A	(Zambidis et al., 2005)
CDH5 Reverse	TTGCCCACATATTCTCCTTTG			
HBB Forward	AGGAGAAGTCTGCCGTTACTG	190 bp	S4A/B	(Dou et al., 2016)
HBB Reverse	CCGAGCACTTTCTTGCCATGA			
HBE Forward	CTTTGGAAACCTGTCGTC	227 bp	S4A/B	(Dou et al., 2016)
HBE Reverse	CTTGCCAAAGTGAGTAGC			
HBG Forward	GGGAGATGCCATAAAGC	135 bp	S4A/B	(Dou et al., 2016)
HBG Reverse	ATTGCCAAAACGGTCAC			
HBZ Forward	GTGTCCATGTGGGCCAAG	108 bp	S4A/B	(Fujita et al., 2016)
HBZ Reverse	GAAGTGCGGGAAGTAGGTCTT			
HBA Forward	TCCCCACCACCAAGACCTA	63bp	S4A/B	(Fujita et al., 2016)
HBA Reverse	CCTTAACCTGGGCAGAGCC			

Supplemental References

Arnaoutova, I., and Kleinman, H.K. (2010). In vitro angiogenesis: endothelial cell tube formation on gelled basement membrane extract. Nat. Protoc. *5*, 628–635.

Batta, K., Florkowska, M., Kouskoff, V., and Lacaud, G. (2014). Direct Reprogramming of Murine Fibroblasts to Hematopoietic Progenitor Cells. Cell Rep. *9*, 1871–1885.

Challen, G.A., and Goodell, M.A. (2010). Runx1 isoforms show differential expression patterns during hematopoietic development but have similar functional effects in adult hematopoietic stem cells. Exp. Hematol. *38*, 403–416.

Dou, D.R., Calvanese, V., Sierra, M.I., Nguyen, A.T., Minasian, A., Saarikoski, P., Sasidharan, R., Ramirez, C.M., Zack, J.A., Crooks, G.M., et al. (2016). Medial HOXA genes demarcate haematopoietic stem cell fate during human development. Nat. Cell Biol. *18*, 595–606.

Doulatov, S., Vo, L.T.T., Chou, S.S.S., Kim, P.G.G., Arora, N., Li, H., Hadland, B.K.K., Bernstein, I.D.D., Collins, J.J.J., Zon, L.I.I., et al. (2013). Induction of Multipotential Hematopoietic Progenitors from Human Pluripotent Stem Cells via Respecification of Lineage-Restricted Precursors. Cell Stem Cell *13*, 459–470.

Elcheva, I., Brok-Volchanskaya, V., Kumar, A., Liu, P., Lee, J.H., Tong, L., Vodyanik, M., Swanson, S., Stewart, R., Kyba, M., et al. (2014). Direct induction of haematoendothelial programs in human pluripotent stem cells by transcriptional regulators. Nat. Commun. *5*, 1–11.

Fujita, A., Uchida, N., Haro-Mora, J.J., Winkler, T., and Tisdale, J. (2016). β-Globin-Expressing Definitive Erythroid Progenitor Cells Generated from Embryonic and Induced Pluripotent Stem Cell-Derived Sacs. Stem Cells *34*, 1541–1552.

Galla, M., Schambach, A., Falk, C.S., Maetzig, T., Kuehle, J., Lange, K., Zychlinski, D., Heinz, N., Brugman, M.H., Göhring, G., et al. (2011). Avoiding cytotoxicity of transposases by dose-controlled mRNA delivery. Nucleic Acids Res. *39*, 7147–7160.

Gao, S., Li, C., Zhu, Y., Wang, Y., Sui, A., Zhong, Y., Xie, B., and Shen, X. (2017). PEDF mediates pathological neovascularization by regulating macrophage recruitment and polarization in the mouse model of oxygen-induced retinopathy. Sci. Rep. *7*, 42846.

Gomes, A.M., Kurochkin, I., Chang, B., Daniel, M., Law, K., Satija, N., Lachmann, A., Wang, Z., Ferreira, L., Ma'ayan, A., et al. (2018). Cooperative Transcription Factor Induction Mediates Hemogenic Reprogramming. Cell Rep. *25*, 2821-2835.e7.

Law, C.W., Chen, Y., Shi, W., and Smyth, G.K. (2014). voom: precision weights unlock linear model analysis tools for RNA-seq read counts.

Liao, Y., Smyth, G.K., and Shi, W. (2013). The Subread aligner: Fast, accurate and scalable read mapping by seed-and-vote. Nucleic Acids Res. *41*.

McCarthy, D.J., Chen, Y., and Smyth, G.K. (2012). Differential expression analysis of multifactor RNA-Seq experiments with respect to biological variation. Nucleic Acids Res. *40*, 4288–4297.

Notta, F., Zandi, S., Takayama, N., Dobson, S., Gan, O.I., Wilson, G., Kaufmann, K.B., Mcleod, J., Laurenti, E., Dunant, C.F., et al. (2015). Distinct routes of lineage development reshape the human blood hierarchy across ontogeny. Science *351*, 1–16.

Pereira, C.F., Chang, B., Qiu, J., Niu, X., Papatsenko, D., Hendry, C.E., Clark, N.R., Nomura-Kitabayashi, A., Kovacic, J.C., Ma'Ayan, A., et al. (2013). Induction of a hemogenic program in mouse fibroblasts. Cell Stem Cell *13*, 205–218.

Philipp, F., Selich, A., Rothe, M., Hoffmann, D., Rittinghausen, S., Morgan, M.A., Klatt, D., Glage, S., Lienenklaus, S., Neuhaus, V., et al. (2018). Human Teratoma-Derived Hematopoiesis Is a Highly Polyclonal Process Supported by Human Umbilical Vein Endothelial Cells. Stem Cell Reports *11*, 1051–1060.

Riddell, J., Gazit, R., Garrison, B.S., Guo, G., Saadatpour, A., Mandal, P.K., Ebina, W., Volchkov, P., Yuan, G.-C., Orkin, S.H., et al. (2014). Reprogramming committed murine blood cells to induced hematopoietic stem cells with defined factors. Cell *157*, 549–564.

Ritchie, M.E., Phipson, B., Wu, D., Hu, Y., Law, C.W., Shi, W., and Smyth, G.K. (2015). limma powers differential expression analyses for RNA-sequencing and microarray studies. Nucleic Acids Res. *43*.

Sandler, V.M., Lis, R., Liu, Y., Kedem, A., James, D., Elemento, O., Butler, J.M., Scandura, J.M., and Rafii, S. (2014). Reprogramming human endothelial cells to haematopoietic cells requires vascular induction. Nature.

Schambach, A., Bohne, J., Chandra, S., Will, E., Margison, G.P., Williams, D.A., and Baum, C. (2006). Equal potency of gammaretroviral and lentiviral SIN vectors for expression of O6-methylguanine-DNA methyltransferase in hematopoietic cells. Mol. Ther. *13*, 391–400.

Schott, J.W., Hoffmann, D., Maetzig, T., Müller, F.J., Steinemann, D., Zychlinski, D., Cantz, T., Baum, C., and Schambach, A. (2014). Improved retroviral episome transfer of transcription factors enables sustained cell fate modification. Gene Ther. *21*, 938–949.

Schwarzer, A., Emmrich, S., Schmidt, F., Beck, D., Ng, M., Reimer, C., Adams, F.F., Grasedieck, S., Witte, D., Käbler, S., et al. (2017). The non-coding RNA landscape of human hematopoiesis and leukemia. Nat. Commun. *8*, 218.

Subramanian, A., Tamayo, P., Mootha, V.K., Mukherjee, S., Ebert, B.L., Gillette, M.A., Paulovich, A., Pomeroy, S.L., Golub, T.R., Lander, E.S., et al. (2005). Gene set enrichment analysis: a knowledge-based approach for interpreting genome-wide expression profiles. Proc. Natl. Acad. Sci. U. S. A. *102*, 15545–15550.

Sugimura, R., Jha, D.K., Han, A., Soria-Valles, C., da Rocha, E.L., Lu, Y.-F., Goettel, J.A., Serrao, E., Rowe, R.G., Malleshaiah, M., et al. (2017). Haematopoietic stem and progenitor cells from human pluripotent stem cells. Nature.

Zambidis, E.T., Peault, B., Park, T.S., Bunz, F., and Civin, C.I. (2005). Hematopoietic differentiation of human embryonic stem cells progresses through sequential hematoendothelial, primitive, and definitive stages resembling human yolk sac development. Blood *106*, 860–870.
